# The cargo adapter protein CLINT1 is phosphorylated by the Numb-associated kinase BIKE and mediates dengue virus infection

**DOI:** 10.1016/j.jbc.2022.101956

**Published:** 2022-04-20

**Authors:** Stanford Schor, Szuyuan Pu, Vlad Nicolaescu, Siavash Azari, Mardo Kõivomägi, Marwah Karim, Patricia Cassonnet, Sirle Saul, Gregory Neveu, Andrew Yueh, Caroline Demeret, Jan M. Skotheim, Yves Jacob, Glenn Randall, Shirit Einav

**Affiliations:** 1 Department of Medicine, Division of Infectious Diseases and Geographic Medicine, and Department of Microbiology and Immunology, Stanford University, California, USA; 2 Department of Microbiology, University of Chicago, Chicago, Illinois, USA; 3 Department of Biology, Stanford University, California, USA; 4 Department of Virology, Molecular Genetics of RNA Virus Genetics (GMVR), Pasteur Institute, National Center for Scientific Research, and Paris Diderot University, Paris, France; 5 Institute of Biotechnology and Pharmaceutical Research, National Health Research Institutes, Zhunan, Taiwan; 6 Chan Zuckerberg Biohub, San Francisco, California, USA

**Keywords:** BIKE, NAK kinase, CLINT1, cell signaling, intracellular trafficking, host–pathogen interaction, dengue virus (DENV), proteomics, virus assembly, AAK1, adapter-associated kinase 1, AP, adapter protein, BIKE, BMP2-inducible kinase, BFG-Y2H, Barcode Fusion Genetics-Yeast 2-Hybrid, CCVs, clathrin-coated vesicles, CLINT1, clathrin-interacting protein 1, DENV, dengue virus, GAK, G-cyclin–associated kinase, GO, gene ontology, HCV, hepatitis C virus, NAK, Numb-associated kinase, NLR, normalized luminescence ratio, NS, nonstructural, NT, nontargeting, ORF, open reading frame, PCA, protein-fragment complementation assay, STK16, serine/threonine kinase 16, TGN, trans-Golgi network, WCL, whole-cell lysate

## Abstract

The signaling pathways and cellular functions regulated by the four Numb-associated kinases are largely unknown. We reported that AAK1 and GAK control intracellular trafficking of RNA viruses and revealed a requirement for BIKE in early and late stages of dengue virus (DENV) infection. However, the downstream targets phosphorylated by BIKE have not yet been identified. Here, to identify BIKE substrates, we conducted a barcode fusion genetics-yeast two-hybrid screen and retrieved publicly available data generated *via* affinity-purification mass spectrometry. We subsequently validated 19 of 47 putative BIKE interactors using mammalian cell–based protein–protein interaction assays. We found that CLINT1, a cargo-specific adapter implicated in bidirectional Golgi-to-endosome trafficking, emerged as a predominant hit in both screens. Our experiments indicated that BIKE catalyzes phosphorylation of a threonine 294 CLINT1 residue both *in vitro* and in cell culture. Our findings revealed that CLINT1 phosphorylation mediates its binding to the DENV nonstructural 3 protein and subsequently promotes DENV assembly and egress. Additionally, using live-cell imaging we revealed that CLINT1 cotraffics with DENV particles and is involved in mediating BIKE’s role in DENV infection. Finally, our data suggest that additional cellular BIKE interactors implicated in the host immune and stress responses and the ubiquitin proteasome system might also be candidate phosphorylation substrates of BIKE. In conclusion, these findings reveal cellular substrates and pathways regulated by the understudied Numb-associated kinase enzyme BIKE, a mechanism for CLINT1 regulation, and control of DENV infection *via* BIKE signaling, with potential implications for cell biology, virology, and host-targeted antiviral design.

It is estimated that approximately 400 million people are infected annually with dengue virus (DENV), a major global health threat (1, 2). Due to climate change and rapid urbanization, the geographical range of dengue infection has been expanding both in developing and developed countries (1). The 10.7-kb genome of DENV, an enveloped, positive single-stranded RNA virus, encodes a single polyprotein, which is proteolytically cleaved into individual proteins (3). While the viral replication machinery is composed of the DENV nonstructural (NS) proteins, virions are formed by the capsid, premembrane (prM), and envelope (E) proteins. DENV enters its target cells *via* clathrin-mediated endocytosis (3). Infectious DENV production is thought to be initiated by assembly of viral particles in ER sites where the E protein is present (4), followed by processing in the *trans*-Golgi network (TGN) where viral particles become infectious, and ultimately exit from the cell *via* the secretory pathway (5). Nevertheless, it remains incompletely characterized what the mechanisms that control trafficking of DENV particles during viral entry and assembly/egress are.

The family of Numb-associated kinases (NAKs) is composed of the Ser/Thr kinases BMP2-inducible kinase (BIKE), adapter-associated kinase 1 (AAK1), G-cyclin–associated kinase (GAK), and serine/threonine kinase 16 (STK16). These diverse kinases share only limited homology in the kinase domain with low homology in other protein regions (6). The structure of BIKE and AAK1 is closely related, whereas STK16 is the most distantly related NAK. Members of the NAK family have been shown to regulate intracellular membrane trafficking (7, 8). AAK1 and GAK were shown to catalyze phosphorylation of the μ subunits of adapter protein (AP) complex 1 (AP1M1) and 2 (AP2M1), secretory and endocytic adapters, respectively, thereby stimulating their binding to cellular cargo (9), and STK16 was shown to regulate secretion in the constitutive secretory pathway at the TGN (10, 11). A subset of clathrin-coated vesicles (CCVs) were found to associate with BIKE, which acted as an accessory protein that bound to the endocytic adapter Numb and phosphorylated a synthetic AP2M1 peptide (6, 12, 13). Yet, no other BIKE substrates have been identified, and therefore, the signaling pathways that it regulates and its functions are largely unknown.

We have previously reported regulation of intracellular trafficking of hepatitis C virus (HCV) and DENV by AAK1 and GAK during both viral entry and assembly/egress partly *via* AP1M1 and AP2M1 phosphorylation (14, 15, 16, 17). Additionally, we have shown a requirement for AAK1 and GAK among other RNA virus families and have validated these kinases as the molecular targets mediating the broad-spectrum antiviral effect of both repurposed and chemically distinct kinase inhibitors that we have been developing (14, 18, 19). More recently, we have demonstrated that BIKE, but not STK16, mediates both an early (postinternalization) stage in the DENV life cycle and a late (assembly/egress) stage (20). We have reported that small molecules that potently inhibit the kinase activity of BIKE suppress replication of DENV infection both *in vitro* and *ex vivo* and demonstrate antiviral activity against a broad range of viruses with a high genetic barrier to resistance. We have also shown that BIKE is involved in mediating the mechanism of antiviral activity of these small molecules (20). Lastly, we have shown that BIKE’s effect is mediated in part by phosphorylation of a threonine 156 (T156) AP2M1 residue (20). Nevertheless, no other substrates of BIKE have been previously identified beyond AP2M1.

Here, to identify additional BIKE substrates, we screened for its interactions with the human proteome in yeast and discovered putative interactors, many of which were then validated *via* mammalian cell–based assays. Clathrin-interacting protein 1 (CLINT1), a cargo-specific adapter (21, 22, 23, 24) previously shown to bind clathrin and AP1 and mediate trafficking of a subset of vesicles between the TGN and endosomes (21, 25, 26, 27, 28), emerged as a predominant hit. We show that BIKE catalyzes phosphorylation of a threonine 294 (T294) CLINT1 residue and that additional BIKE interactors are candidate phosphorylation substrates. Additionally, our data indicate that the phosphorylation of CLINT1 by BIKE mediates binding of CLINT1 to the DENV NS3 protein and subsequently DENV assembly and egress.

## Results

### High-throughput proteomic screens identify BIKE interactors

**Figure 1:**
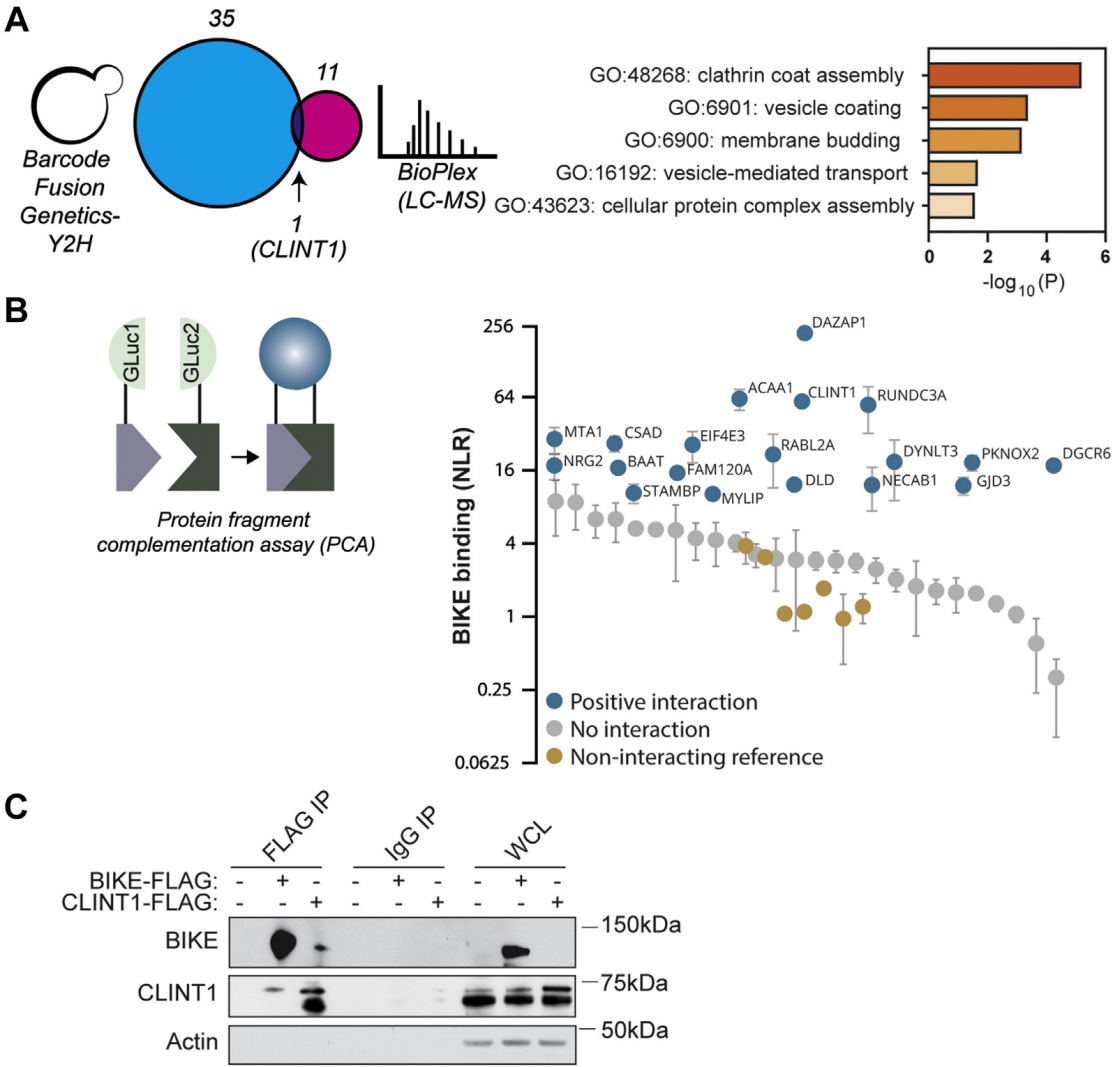
**High-throughput screen identifies BIKE interactors**. *A*, *left*: Barcode Fusion Genetics-Yeast-2 Hybrid (BFG-Y2H) assay was carried out using barcoded baits and preys from the human ORFeome library that were physically linked with a split Cre recombinase following pooling of baits and preys postselection. Hits were defined by stringent cutoffs for the stickiness index and number of observations during sequencing. The Venn diagram shows the number of unique and overlapping hits emerging *via* the BFG-Y2H screen and the publicly available BioPlex dataset generated *via* an affinity purification MS approach. *Right*: Molecular function terms and *p* values derived from Gene Ontology (GO) enrichment analysis of BIKE, CLINT1, and their overlapping interactors following integration of the BIKE interactome data with BioGRID data *via* BiNGO. *B*, *left*: Schematic of the protein-fragment complementation assay (PCA) format. *Right*: BIKE interactions with 45 of the 47 putative interactors measured *via* PCAs in 293T cells. Dots depict the mean normalized luminescence ratio (NLR) and standard deviation (SD) of a representative experiment of 2 conducted with 3 replicates each. *Gold*, a reference set of 7 proteins that do not interact with BIKE. *Gray*, noninteracting proteins among the 47 putative BIKE interactors. Blue, BIKE-interacting proteins as defined by a cutoff of NLR>10 representing greater than two SDs above the mean NLR of the reference set. *C*, IPs with anti-FLAG antibody or IgG control from lysates of Huh7 cells ectopically expressing BIKE-FLAG, CLINT1-FLAG, or Empty-FLAG. Molecular weight markers are indicated on the *right* (kDa). Membranes were blotted with antibodies against BIKE, CLINT1, and actin. BIKE, BMP2-inducible kinase; CLINT1, clathrin-interacting protein 1; IP, immunoprecipitation; WCL, whole-cell lysate.

To identify BIKE interactors and candidate substrates, we screened for its interactions with the human proteome *via* a Barcode Fusion Genetics-Yeast 2-Hybrid (BFG-Y2H) approach (29). This screening pipeline is based on mating haploid yeast of opposite mating type, and it utilizes the human ORFeome collection of full-length open reading frames (ORFs) rather than cDNA libraries for expression (30). This approach overcomes several limitations of classical Y2H systems. First, it avoids enrichment bias for housekeeping proteins by utilizing an arrayed collection of human ORFs. Second, it facilitates high-throughput multiplexed screening using DNA barcodes attached to ORFs encoding baits and preys. Third, it increases coverage by using barcode stitching of the interacting bait and prey generated *via* Cre-recombinase, PCR amplification of barcode tandems, pooling, and next-generation sequencing followed by data deconvolution for extracting interacting pairs (29). Positive interactions (hits) were defined based on a score that incorporated (1): the stickiness of the prey—a proxy for a hit specificity based on the number of baits other than BIKE (out of ∼200 tested) that also bound this prey; and (2) the number of observations—a proxy for robustness and sensitivity, representing the number of times BIKE was identified with this hit upon sequencing. Using stickiness <10% and >3 observations as the criteria, 36 candidate BIKE interactors were identified *via* this screen (Fig. 1*A* and Table S1).

In parallel, we retrieved publicly available data from the biophysical interactions of ORFeome-based complexes network (BioPlex), which was constructed using a high-throughput affinity-purification mass spectrometry approach (31). Twelve putative interactors of BIKE were identified in this dataset. A BIKE interaction network was then generated by combining the hits from the BFG-Y2H and this dataset. CLINT1, a 70-kDa protein known to bind clathrin and AP1 and participate in clathrin-mediated budding from internal compartments (21, 25, 31), emerged in both screens as a top BIKE interactor (Fig. 1*A*). The AP2 complex subunits AP2S1 and AP2M1 were among the BioPlex hits. To further understand the functions of BIKE-related pathways, first neighbors of BIKE (node of degree >2) were extracted from BioGRID, a public repository of interactomics data, and introduced into this BIKE interactome network (32) (Fig. S1). Gene Ontology (GO) enrichment analysis of BIKE, CLINT1, and their overlapping interactors *via* BiNGO showed significant enrichment of “vesicle coating,” “membrane budding,” “vesicle-mediated transport,” and “cellular protein complex assembly” proteins (Fig. 1*A*). These results suggest that BIKE may play an important role in intracellular membrane trafficking, in part *via* its interaction with CLINT1.

### Validation of BIKE-interacting proteins in mammalian cells

To assess the robustness of the BIKE interactome, we used protein-fragment complementation assays (PCAs), an orthogonal platform for detecting protein–protein interactions in mammalian (293T) cells (33) (Fig. 1*B*). This assay provides a high-fidelity means to measure transient and weak interactions and evaluate their apparent affinities (Kds in the μM range (34)) within appropriate subcellular compartments (16, 17, 33, 35). Interaction signals of 45 of the 47 hits were normalized to the average luminescence of each pair member matched with the complementary luciferase fragment to generate a normalized luminescence ratio (NLR). We benchmarked the accuracy and sensitivity of this screen with a random set of 7 proteins not predicted to interact with BIKE (mean NLR of 1.81 ± 1.11) (Table S2). We chose a stringent cutoff of NLR > 10 representing greater than two standard deviations above the mean NLR of this noninteracting control set. Using this cutoff, 19 interactions were validated in this secondary screen, of which CLINT1 demonstrated the third highest apparent affinity (Fig. 1*B*). RABL2A and DYNLT3 were also validated as candidate BIKE interactors, supporting that BIKE controls various aspects of intracellular membrane trafficking (36, 37). Among the other validated hits were proteins involved in the ubiquitin proteasome system (STAMBP and MYLIP (38)), transcription (MTA1, PKNOX2) (39), translation (EIF4E3) (40), male infertility (RABL2A, DAZAP1) (41), and virus-induced oncogenesis (MTA1) (42, 43).

To further confirm the interaction between BIKE and CLINT1, constructs expressing either BIKE-FLAG or CLINT1-FLAG were transfected into Huh7 cells followed by pulldown with anti-FLAG affinity gel. Western blot analysis of these samples revealed effective pulldown of the FLAG-tagged proteins and co-immunoprecipitation (co-IP) of CLINT1 with BIKE-FLAG and reciprocally, BIKE with CLINT1-FLAG (Fig. 1*C*). Notably, the anti-CLINT1 antibody we used detects the three isoforms of CLINT1: two predominant isoforms, 1 and 2 (68 kDa) and a lower intensity band corresponding to isoform 3 (70 kDa) (Fig. 1*C*, whole-cell lysates [WCLs]). Since the CLINT1-FLAG plasmid used in this study encodes isoform 1 of CLINT1, ectopic expression of CLINT1-FLAG enhances the signal of the top band (equivalent to the molecular weight of isoform 1 plus an additional 22-aa linker [∼71 kDa]) (Fig. 1*C*, WCL). An additional lower-molecular-weight band is observed when CLINT1-FLAG is pulled down and blotted with anti-FLAG antibody (Fig. 1*C*). We speculate that there may be a complex formed between CLINT1-FLAG, endogenous BIKE, and endogenous CLINT1 or that CLINT1 may form oligomers.

Collectively, BIKE-CLINT1 binding was demonstrated *via* four orthogonal methods.

### CLINT1 and other BIKE interactors are candidate BIKE substrates

**Figure 2:**
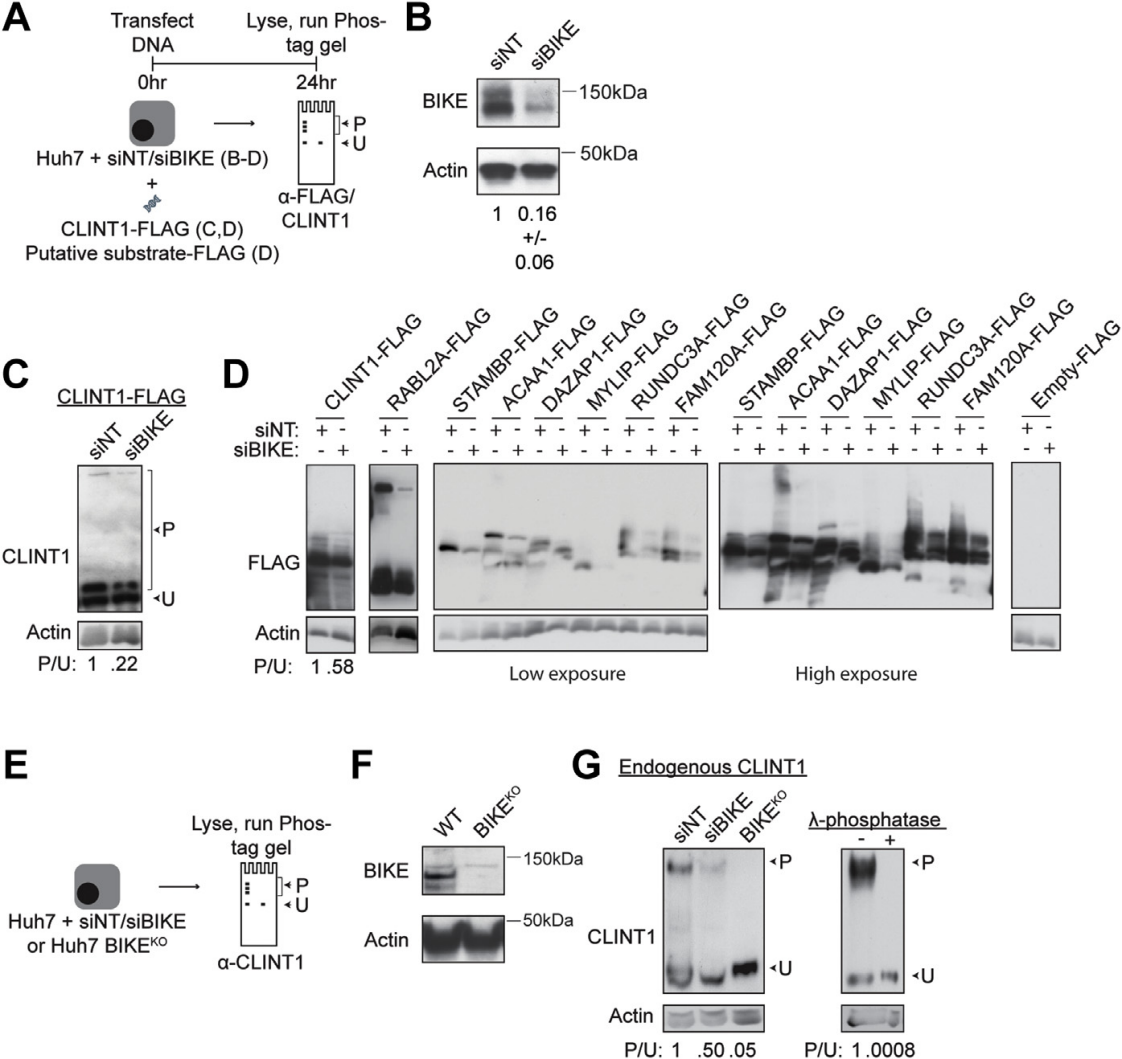
**BIKE phosphorylates CLINT1 and additional candidate substrates in cells**. *A*, schematic of the experiments shown in *B*–*D*. *B*, confirmation of siRNA-mediated protein expression knockdown by Western blot at 24 h post-transfection of Huh7 cells. Quantitative data from two independent experiments relative to siNT control are also shown. *C*, Phos-tag gel analysis in BIKE-depleted and control Huh7 cells expressing CLINT1-FLAG. *D*, Phos-tag gel analysis in BIKE-depleted and control Huh7 cells individually expressing 8 FLAG-tagged candidate substrates emerging in the screens or Empty-FLAG. *Lower* (*left*) and *higher* (*right*) exposures of the larger membrane are shown. *E*, schematic of the experiment shown in *F* and *G*. *F*, BIKE protein expression by Western blot in control Huh7 (WT) cells and a cell line deleted of BIKE (BIKE^KO^) *via* CRISPR/Cas9. *G*, phosphorylation of endogenous CLINT1 by BIKE measured *via* Phos-tag gel analysis in cell lysates derived from Huh7 cells depleted (B) or deleted (F) of BIKE and control cells (*left panel*) or treated with λ-phosphatase or reaction buffer only (*right panel*). Shown in *C*, *D*, and *G* are membranes blotted with anti-CLINT1 (*C* and *G*), anti-FLAG (*D*), and anti-actin (*C*, *D* and *G*) antibodies and quantitative CLINT1 phosphorylation data relative to the corresponding controls representative of at least two experiments. P/U ratio, phosphorylated-to-unphosphorylated band signal ratio quantified *via* ImageJ shown as mean ± SEM, where applicable (calculated from at least 2 independent experiments). BIKE, BMP2-inducible kinase; CLINT1, clathrin-interacting protein 1.

To identify candidate substrates, we ectopically expressed 10 individual FLAG-tagged candidate BIKE interactors in Huh7 cells depleted for BIKE *via* siRNA or expressing a nontargeting (NT) control siRNA (siNT) (Fig. 2, *A* and *B*). We then ran the cell lysates on Phos-tag gels, which separate phosphorylated (P) from unphosphorylated (U) protein forms by binding of phosphorylated residues to divalent Phos-tag molecules (44). Membranes were blotted with anti-FLAG antibody, and P/U ratios were calculated by dividing the intensity of the high by the low molecular bands (Fig. 2*A*). Multiple bands were observed for some of the cellular factors, indicating various phosphorylation states, as typically resolved *via* Phos-tag gel analysis. BIKE depletion reduced the intensity of higher-molecular-weight bands and the P/U ratio to 0.22 and 0.58 in cells expressing CLINT1-FLAG relative to control cells expressing siNT (Fig. 2, *C* and *D*, respectively). Similarly, BIKE depletion caused loss or reduced intensity of higher-molecular-weight bands in cells expressing STAMBP, RABL2A, ACAA1, DAZAP1, MYLIP1, RUNDC3A, and FAM120A (Fig. 2*D*), but not TRAPPC6B and STXBP6 (data not shown) relative to NT controls. These results revealed several proteins with diverse functions as candidate BIKE substrates.

### BIKE phosphorylates endogenous CLINT1 in cultured cells

To further test the hypothesis that CLINT1 is a BIKE substrate, we used Phos-tag gel analysis to analyze the phosphorylation of endogenous CLINT1 by BIKE in Huh7 cells depleted of BIKE by siRNA (Fig. 2*B*) or deleted of BIKE by CRISPR/Cas9 (Fig. 2, *E* and *F*). The P/U ratios of endogenous CLINT1 were reduced by 2- and 20-fold in BIKE-depleted and BIKE-knockout (BIKE^KO^) cells relative to control, respectively (Fig. 2*G*). Upon longer exposure of this membrane, an additional, lower-molecular-weight band was observed representing an additional phosphorylation state of endogenous CLINT1. Interestingly, this band did not entirely disappear in the BIKE^KO^ cells, in contrast to the top band, suggesting that CLINT1 is phosphorylated by an additional kinase beyond BIKE (Fig. S2). Five percent of CLINT1 thus remained phosphorylated in the BIKE^KO^ cells (Figs. S2 and 2*G*), whereas no CLINT1 phosphorylation was essentially detected upon incubation of cell lysate controls with λ-phosphatase validating the Phos-tag experiments (45) (Fig. 2*G*).

### BIKE phosphorylates CLINT1 *in vitro*

**Figure 3:**
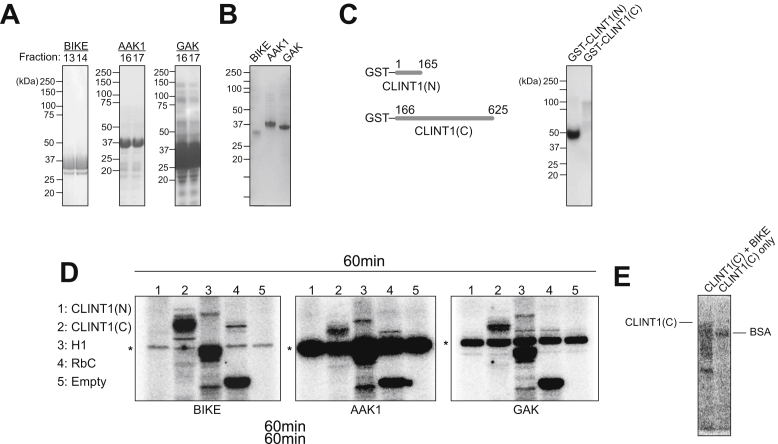
**BIKE phosphorylates CLINT1 *in vitro***. *A*, rBIKE, rAAK1, and rGAK purified by gel filtration. Representative fractions are shown. Membranes were blotted with the respective anti-NAK antibodies. *B*, Coomassie-stained rBIKE, rAAK1, and rGAK ran on an SDS-PAGE gel. *C*, *left*: Schematics showing recombinant GST-tagged N- and C-terminal CLINT1 fragments (CLINT1(N) and CLINT1(C)) purified from bacterial cells. Right: rCLINT1(C) and rCLINT1(N) by GST pulldown following expression in bacterial cells. *D*, autoradiographs from *in vitro* kinase assays carried out by incubating recombinant BIKE, AAK1, or GAK kinases with CLINT1(N), CLINT1(C), H1, RbC, or no substrate. After 60 min, reactions were stopped with SDS sample buffer. *E*, autoradiograph results from *in vitro* kinase assay carried out by incubating rCLINT1(C) in the presence or absence of rBIKE and ATP for 60 min followed by reaction termination *via* addition of SDS sample buffer. Molecular weight markers are indicated on the *left* (kDa). AAK1, adapter-associated kinase 1; BIKE, BMP2-inducible kinase; CLINT1, clathrin-interacting protein 1; GAK, G-cyclin–associated kinase.

To confirm phosphorylation by BIKE, we performed radioactive *in vitro* kinase assays. The recombinant kinase domains of BIKE, AAK1, and GAK and the N- and C-terminal domains of CLINT1 (rCLINT1(N) and rCLINT1(C)) were expressed in bacterial cells and purified (6) (Fig. 3, *A*–*C*). Individual rNAKs were incubated with rCLINT1(N), rCLINT1(C), the universal kinase substrates histone H1 or retinoblastoma protein C-terminal fragment (RbC), or no substrate in the presence of γ-p32 ATP, and stopped reactions were run on a protein gel. To quantitatively determine the phosphorylation rate of different substrates among rNAKs, membranes were exposed to screens and scanned with a phosphoimager. All three kinases comparably phosphorylated histone H1 and RbC, as indicated by the appearance of high-intensity bands (Fig. 3*D*, lanes 3 and 4) that were absent or weaker in the no-substrate control (Fig. 3*D*, lane 5), confirming the kinase activities of these recombinant proteins. A high-intensity band that was absent in the no-substrate control appeared upon incubation of BIKE with CLINT1(C) but not CLINT1(N), indicating that BIKE specifically phosphorylates the C-terminal domain of CLINT1 (Fig. 3*D*, lanes 1 and 2). While a similar size band appeared upon incubation of CLINT1(C) with AAK1 and GAK, its intensity was weaker, suggesting that CLINT1 is likely a BIKE-specific, rather than a pan-NAK, substrate. Notably, the middle band present in all three membranes represents enzyme autophosphorylation, as evidenced by Coomassie staining of the corresponding gels (Fig. S3). No phosphorylation signal appeared in the absence of BIKE (Fig. 3*E*), excluding the possibility of CLINT1 phosphorylation by kinase(s) copurified with it.

These findings provide evidence that BIKE phosphorylates rCLINT1(C) *in vitro*, as well as both ectopically expressed and endogenous CLINT1 in cultured cells.

### BIKE phosphorylates a T294 residue in CLINT1

**Figure 4:**
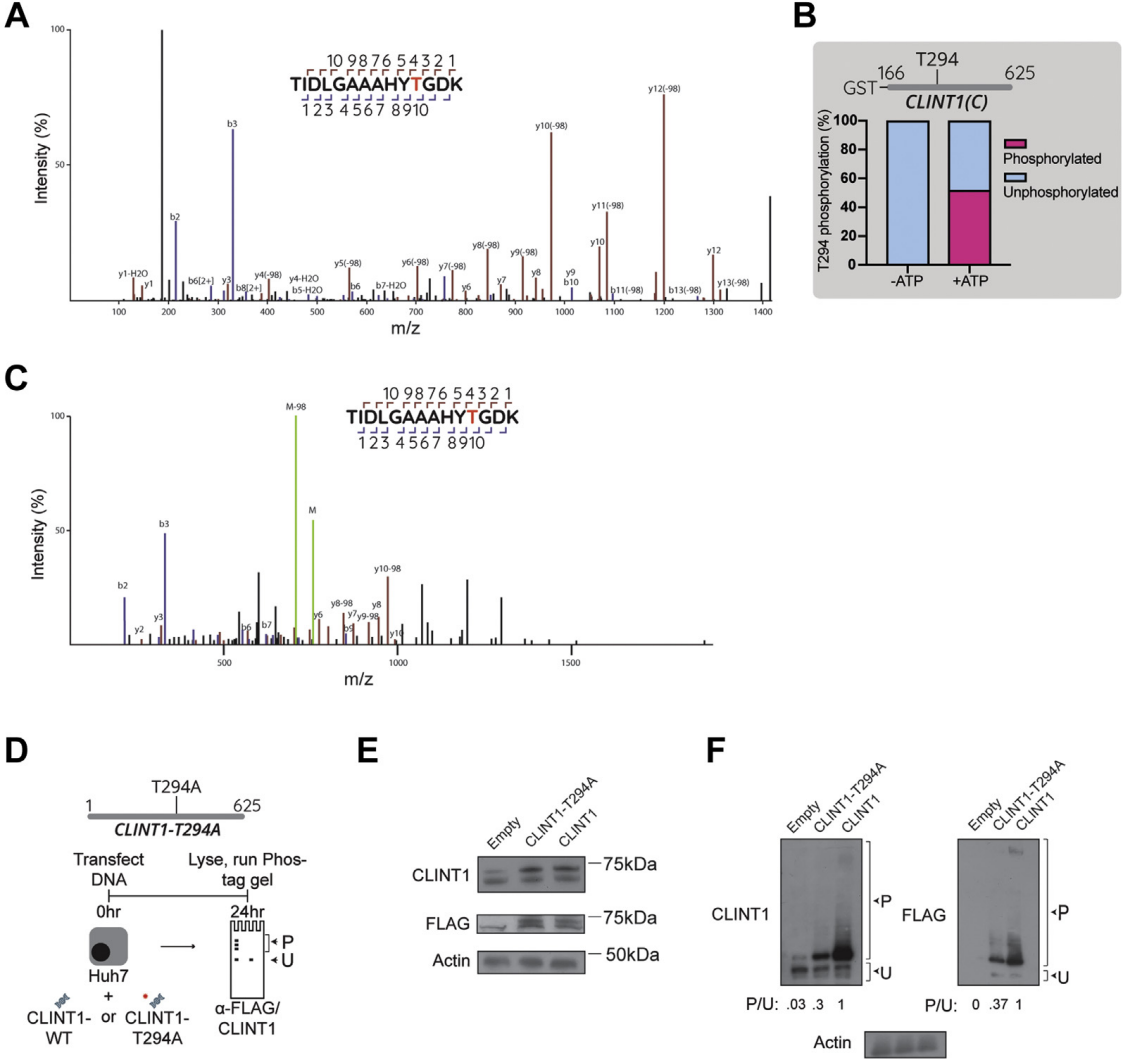
**BIKE phosphorylates CLINT1 at T294**. *A*, higher-energy collisional dissociation MS/MS spectrum of CLINT1(C) phosphopeptide fragments corresponding to residues 284 to 297 following incubation of CLINT1(C) with rBIKE and ATP demonstrating 98-Da loss in many *b* and *y* series fragment ions indicative of T294 phosphorylation. The fragment ion coverage in the spectrum is shown above the plot. *B*, a bar graph showing the proportion of phosphorylated and unphosphorylated T294 residues in the presence or absence of ATP. *C*, MS/MS spectrum of synthetically derived CLINT1 phosphopeptide corresponding to residues 284 to 297 with phosphorylated T294. *D*, schematic of the experiment shown in *E*. *E*, detection of endogenous and exogenous (FLAG-tagged) CLINT1 using anti-CLINT1 and anti-FLAG antibodies. *F*, Phos-tag gel analysis in Huh7 cells ectopically expressing empty control, FLAG-tagged CLINT1-WT or CLINT1-T294A mutant. Shown are membranes blotted with anti-CLINT1, anti-FLAG, and anti-actin antibodies and quantitative CLINT1 phosphorylation data relative to WT protein representative of at least two experiments. P/U ratio, phosphorylated-to-unphosphorylated band signal ratio quantified *via* ImageJ. BIKE, BMP2-inducible kinase; CLINT1, clathrin-interacting protein 1.

To identify the specific residue(s) that BIKE modifies, rCLINT1(C) was incubated with rBIKE in the presence or absence of ATP followed by LC-MS/MS analysis, which detects modified peptides that lose phosphoryl groups (HPO_3_/80 Da and/or H_3_P0_4_/98 Da) during ionization. We identified 11 distinct CLINT1 peptides that harbored a threonine 294 residue (T294) that lost the 98-Da moiety (Fig. 4*A* and Table S5). The fragmentation on both sides of CLINT1 T294 was consistent with localization to this specific residue. Fifty-two percent of the total T294 residues detected in the presence of ATP were phosphorylated, in contrast to 0% in the absence of ATP (Fig. 4*B*). The spectra of a control peptide harboring phosphorylated T294 were identical to those detected with rCLINT1(C) (Fig. 4*C*), confirming that BIKE modified T294 and not an adjacent Y293 residue.

To confirm that T294 is a functionally relevant phosphorylation site, we studied the effect of a T294A point mutation on CLINT1 phosphorylation *via* Phos-tag gel analysis. Huh7 cells expressing FLAG-tagged wildtype (WT) T294A CLINT1 mutant or empty control were lysed, run on standard and Phos-tag gels, and probed for CLINT1 and FLAG (Fig. 4, *D*–*F*). The T294A mutation did not alter the expression of CLINT1 (Fig. 4*E*), yet it reduced CLINT1 P/U ratio by ∼10-fold relative to the empty control and ∼3-fold relative to WT CLINT1 (Fig. 4*F*). These findings reveal CLINT1 T294 as a functional residue modified by BIKE.

### CLINT1 is required for DENV assembly/egress and is involved in mediating BIKE’s role in DENV infection

**Figure 5:**
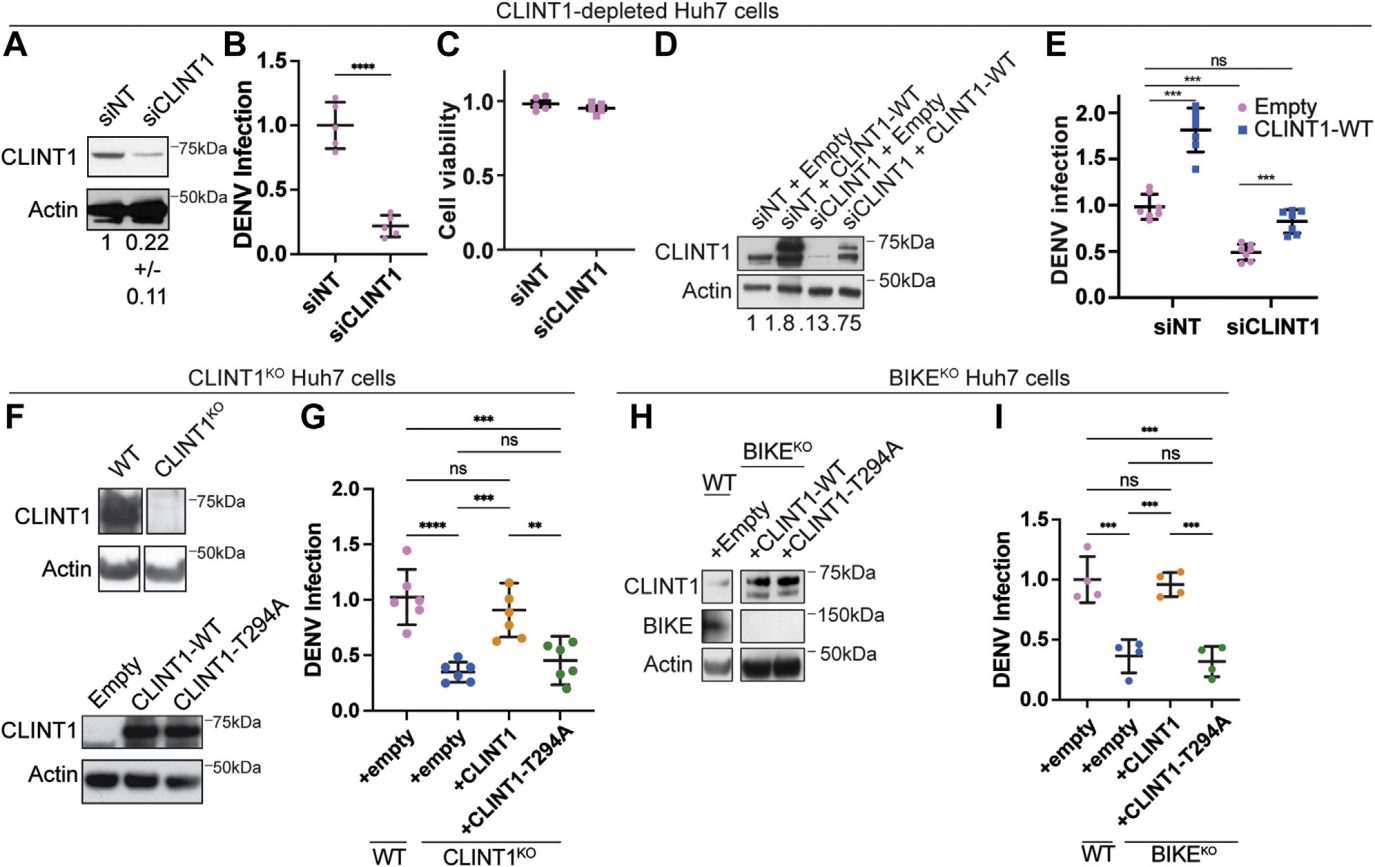
**CLINT1 is required for DENV infection and is involved in mediating the role of BIKE in DENV infection**. *A*–*I*, CLINT1 protein by Western blot in Huh7 cells depleted of CLINT1 *via* siRNA or NT controls (*A* and *D*) or deleted for CLINT1 (*F*) or BIKE (*H*) *via* CRISPR/Cas9 and in these cells upon complementation with FLAG-tagged WT or T294A CLINT1 mutant and an empty plasmid (*D*, *F* and *H*). Representative membranes blotted with anti-CLINT1, anti-BIKE, and anti-actin antibodies and quantitative CLINT1 to actin ratios are shown. Samples were run on the same gels (*F* and *H*), from which several lanes were cut out. DENV infection (*B*, *E*, *G* and *I*) and cell viability (*C*) as measured in the indicated cells by luciferase and alamarBlue assays, respectively, at 48 h postinoculation with luciferase reporter DENV2 (MOI=0.05). Shown in *B*, *C*, and *I* are mean ± SD of results of representative experiments out of at least two conducted, each with 4 or 5 replicates. Shown in *E* and *G* are mean ± SEM of data combined from two independent experiments with 3 or 4 replicates each. ∗*p* < 0.05; ∗∗*p* < 0.01; ∗∗∗*p* < 0.001 relative to corresponding controls by one-way ANOVA with Dunnett’s (*B* and *C*) or Tukey’s (*E*, *G* and *I*) post hoc tests. BIKE, BMP2-inducible kinase; CLINT1, clathrin-interacting protein 1; DENV, dengue virus; MOI, multiplicity of infection; ns, nonsignificant.

To probe the functional relevance of CLINT1 to DENV infection, we examined the effect of silencing CLINT1 expression by a validated siRNA in Huh7 cells (22). siCLINT1 reduced infection with DENV2 (NGC strain) by ∼80% relative to NT control, as measured *via* luciferase assays at 48 h postinfection, with no apparent cytotoxicity (Fig. 5, *A*–*C*). Ectopic expression of CLINT1 increased DENV infection by ∼1.8-fold in control cells, indicating that CLINT1 is rate limiting for DENV infection (Fig. 5, *D* and *E*). Moreover, ectopically expressed CLINT1 reversed the defect in DENV infection induced by siCLINT1, largely excluding the possibility of off-target effects causing the observed phenotype (Fig. 5, *D* and *E*).

Consistent with these results, CLINT1 deletion in a CLINT1^KO^ Huh7 cell line reduced DENV infection by ∼10-fold relative to WT cells (Fig. 5, *F* and *G*). Notably, this defect was completely reversed upon ectopic expression of WT CLINT1 (Fig. 5, *F* and *G*). In contrast, ectopically expressed CLINT1-T294A mutant did not reverse the observed defect in DENV infection in CLINT1^KO^ cells, despite comparable expression level to WT CLINT1, supporting that phosphorylation of T294 in CLINT1 is required for DENV infection (Fig. 5, *F* and *G*).

Additionally, ectopic expression of WT but not the CLINT1-T294A mutant restored DENV infection in the BIKE^KO^ cell line (Fig. 5,*H* and *I*), yet it did not increase DENV infection as measured in the presence of BIKE in WT cells (Fig. 5*E*). These findings suggest that CLINT1 is involved in mediating BIKE’s role in DENV infection and that T294 may undergo phosphorylation by another kinase beyond BIKE and/or mediate an additional, phosphorylation-independent role in DENV infection, yet it is required for DENV infection (see Discussion).

**Figure 6:**
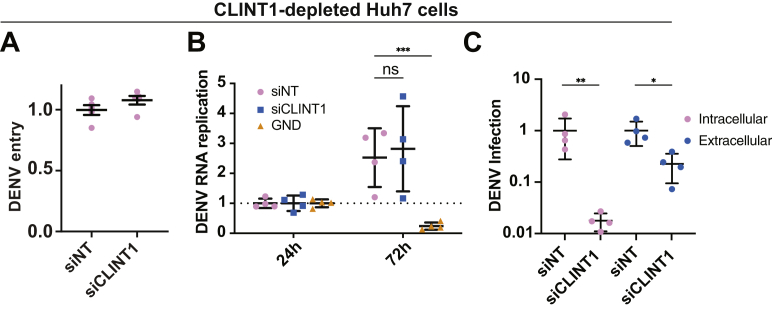
**CLINT1 mediates a late stage of DENV infection**. *A*, DENV entry measured *via* luciferase assay 6 h postinoculation of Huh7 cells transfected with the indicated siRNAs (Fig. 5*A*) with luciferase reporter DENV2 (MOI=5). *B*, DENV RNA replication measured in Huh7 cells by luciferase assays 24 and 72 h following cotransfection with both a Tet-inducible DNA-launched DENV replicon and a TET-ON plasmid and 24-h induction by doxycycline (GND is a replication-defective DENV). Data are normalized to signal at 24 h following Tet induction. *C*, DENV infectivity measured by luciferase assays following inoculation of naïve cells with lysates (intracellular) and supernatants (extracellular) harvested from Huh7 cells transfected with *in vitro* transcribed DENV2 RNA 48 h post-transfection. Data in (*C*) are normalized to siNT controls and shown as log_10_. (*A*–*C*) Shown are means ± SD of results of representative experiments out of two conducted each with four replicates. ∗*p* < 0.05; ∗∗*p* < 0.01; ∗∗∗*p* < 0.001 relative to corresponding controls by one-way (*A* and *C*) or two-way (*B*) ANOVA with Dunnett’s post hoc tests. CLINT1, clathrin-interacting protein 1; DENV, dengue virus; MOI, multiplicity of infection; ns, nonsignificant.

To monitor distinct stages in the DENV life cycle, next, we used Huh7 cells depleted of CLINT1 by an siRNA. To define the effect of CLINT1 depletion on early stages of the DENV life cycle, we first conducted luciferase assays 6 h postinfection with a DENV2 expressing a luciferase reporter (46). Suppression of CLINT1 expression did not alter DENV entry *via* this assay relative to cells transfected with the siNT control (Fig. 6*A*). To determine a potential role of CLINT1 in viral translation and RNA replication, we conducted luciferase assays at 24 and 72 h following induction of DENV subgenomic replicon (pCMV-DV2Rep) expression in Huh7 cells (47). Suppression of CLINT1 expression did not alter DENV translation or DENV RNA replication (Fig. 6*B*). To probe for an effect in the latest stages of the viral life cycle, we measured intracellular and extracellular infectivity in Huh7 cells depleted of CLINT1. Briefly, full-length DENV RNA was transfected into Huh7 cells, and clarified cell lysates and culture supernatants harvested at 48 h post-transfection were used to inoculate naïve cells followed by luciferase assays at 48 h postinoculation. Suppression of CLINT1 expression reduced both intracellular and extracellular infectivity by 50- and 5-fold relative to control cells, respectively (Fig. 6*C*), suggesting a defect in DENV assembly and egress. Low transfection efficiency of DENV RNA hindered our attempts to measure infectious virus production in the CLINT1^KO^ cell lines.

Collectively, these results indicate that CLINT1 is required for infectious DENV production and that T294 phosphorylation is involved in mediating the role of BIKE in DENV infection.

### T294 mediates CLINT1 binding to DENV NS3 protein

**Figure 7:**
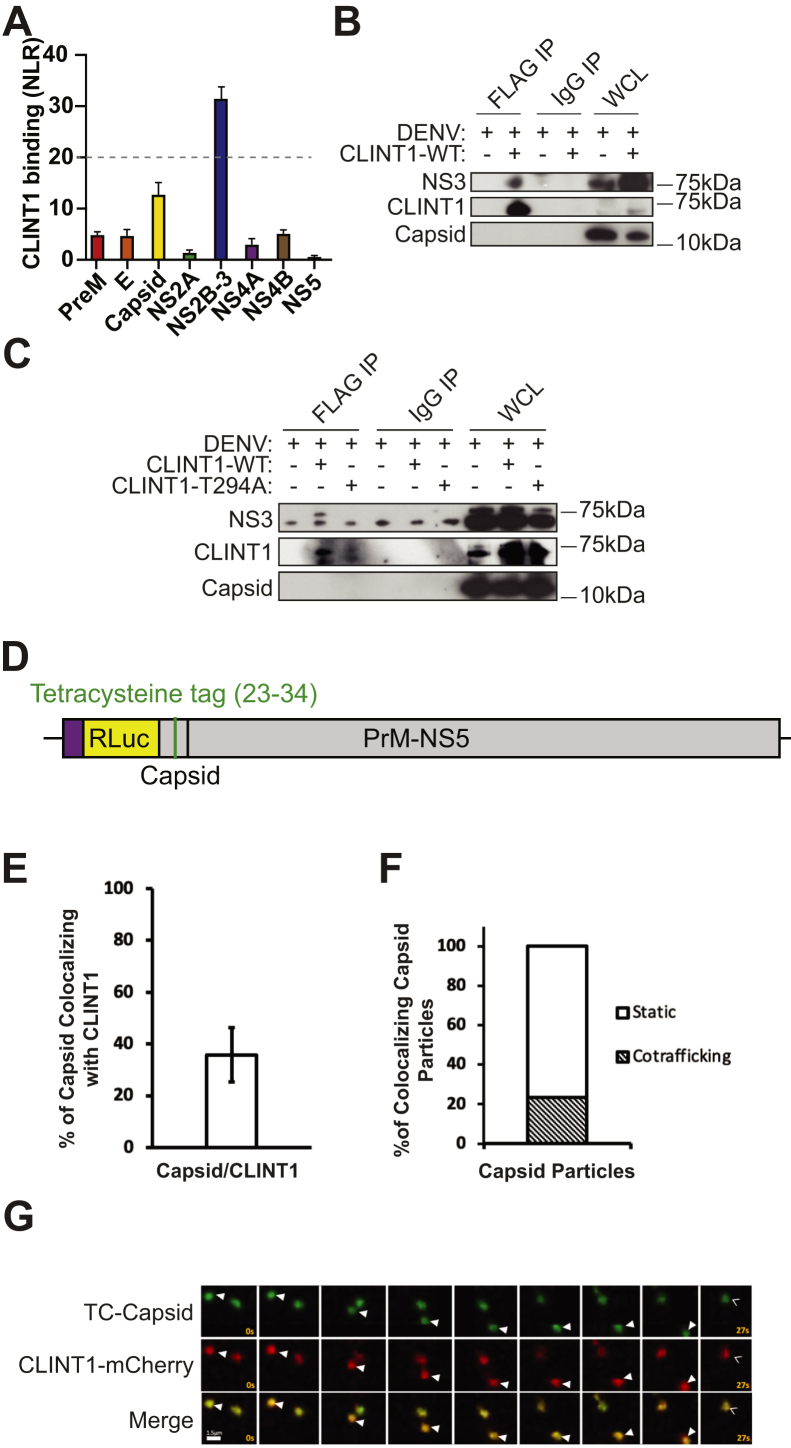
**CLINT1 binds DENV NS3 and mediates intracellular trafficking of DENV particles.***A*, CLINT1 binding to individual DENV proteins *via* PCAs in 293T cells. The *dotted line* represents the cutoff used for positive interactions. *B* and *C*, IPs with anti-FLAG antibody or IgG control from lysates of DENV-infected Huh7 cells ectopically expressing FLAG-tagged WT (*B* and *C*) or CLINT1-T294A mutant (*C*) or empty plasmid. Molecular weight markers are indicated on the *right* (kDa). Membranes were blotted with antibodies against NS3, CLINT1, and capsid. *D*, schematic of the TC-capsid construct. *E* and *F*, Quantitative data of the percentage of DENV capsid colocalizing with CLINT1 (*E*) and of the static and cotrafficking capsid/CLINT1 colocalized particles (*F*). The data were obtained from 3 fields of view (equivalent to 3 independent movies). Moreover, an average of 61 total DENV-capsid, 22 total colocalizing, 6 total cotrafficking, and 16 total static particles were counted across the three movies. *G*, representative live cell fluorescence microscopy montages of TC-capsid DENV (*green*) cotrafficking with CLINT1-mCherry (*red*) over 27 s. Distance traveled (μm) during video acquisition is indicated. *Solid arrow heads* indicate cotrafficking capsid/CLINT1 particles; open *arrow heads* indicate static capsid/CLINT1 colocalizing particles. CLINT1, clathrin-interacting protein 1; DENV, dengue virus; IP, immunoprecipitation; NLR, normalized luminescence ratio; PCA, protein-fragment complementation assay; WCL, whole-cell lysate.

Next, we screened for CLINT1 interactions with the DENV proteome (excluding NS1, which is in the ER lumen) *via* PCAs. Plasmids encoding GLuc1-CLINT1 and individual GLuc2-tagged DENV proteins derived from DENV2 (16681 strain) (48) were transfected pairwise into 293T cells followed by luciferase assays. When screening for NS3–CLINT1 interactions, a plasmid encoding FLAG-tagged NS2B was added to facilitate membrane binding of the NS3 protein (49). Expression of the viral proteins was confirmed by Western blotting (Fig. S4). A cutoff value of >2.2 SDs (corresponding to an NLR of >20) relative to a random reference set composed of 53 noninteracting human protein pairs (35) was chosen as the threshold to define positive interactions. CLINT1 bound the DENV NS3 protein, whereas its coexpression with PrM, E, capsid, NS2A, NS4A, NS4B, and NS5 yielded background-level signals (Fig. 7*A*). No correlation was detected between the level of expression of viral proteins and their interaction with CLINT1.

To validate CLINT1–NS3 binding in the context of DENV infection, we conducted co-IPs in DENV2-infected Huh7 cells following transfection with CLINT1-FLAG constructs (48). Anti-FLAG affinity gel effectively pulled down CLINT1-FLAG (∼71 kDa), with which a ∼70-kDa protein corresponding to NS3 was co-immunoprecipitated (Fig. 7*B*). The capsid protein was not co-immunoprecipitated with CLINT1-FLAG, and no signal was observed in samples incubated with IgG control. Ectopic expression of the T294A CLINT1-FLAG mutant in DENV-infected cells completely disrupted NS3 pulldown (Fig. 7*C*).

These results indicate that T294 is involved in mediating CLINT1–NS3 binding and suggest that T294 phosphorylation by BIKE may be regulating this interaction.

### DENV particles cotraffic with CLINT1

Since CLINT1 sorts in TGN-endosome pathways, we tested the hypothesis that it shuttles DENV particle intracellularly. To do so, we developed a live-cell imaging approach to monitor the cotrafficking of DENV capsid with CLINT1, similarly to our prior work utilizing tetracysteine (TC) harboring HCV particles (TC-core) to monitor HCV cotrafficking with cellular secretory pathway factors (17, 50). We constructed DENV TC-capsid in the pACYC-Rluc2A-NGC backbone (46) by replacing residues 23 to 34, a capsid site whose alteration did not impair viral replication *via* alanine scanning mutagenesis analysis, with a TC (FLNCCPGCCMEP) tag (Figs. 7*D* and S5). We confirmed the presence of the TC-tag by sequencing viral cDNA from cells 5 days post-transfection with the TC-capsid construct. Analysis of TC-capsid puncta stained with the biarsenical dye FlAsH *via* live-cell imaging revealed that 35.8% ± 10.53% (n = 3, ±SEM) of TC-capsid puncta (n = 184) were colocalized with CLINT1-mCherry (Figs. 7, *E* and *G* and S6). Additionally, 23.42% ± 6.8% (n = 3, ±SEM) of these colocalized puncta moved more than 1 μm over 3 frames, whereas the remaining 76.58% ± 6.8 (n = 3, ±SEM) colocalizing capsid particles were static (Figs. 7, *F* and *G*, S6 and Movie S1). These results indicate that a fraction of CLINT1 and TC-capsid cotraffics.

## Discussion

We have previously reported that the intracellular trafficking of various RNA viruses is regulated by AAK1- and GAK-mediated phosphorylation of AP complexes and that these kinases are validated targets for broad-spectrum antivirals (14, 15, 17, 18, 19). More recently, we revealed a requirement for BIKE, previously implicated in osteoblast differentiation, myopia, and cancer (12, 51, 52, 53), in an early stage of DENV infection as well as assembly/egress (20). We showed that inhibition of BIKE can be potentially used as a strategy to suppress infections by a wide range of RNA viruses, and we identified BIKE as a molecular target mediating the antiviral activity (20). Yet, the signaling pathways that the NAK kinases, and particularly BIKE, regulate and their roles in healthy and disease states are largely unknown. Here, we address this knowledge gap by integrating proteomics, biochemistry, molecular virology, genomics, genetics, and advanced imaging approaches. We discover BIKE substrates and show that phosphorylation of CLINT1 by BIKE is required for DENV assembly/egress and is involved in mediating BIKE’s role in DENV infection. These findings shed light on the signaling pathways and functions regulated by an understudied kinase and provide insights into the virus–host determinants that regulate DENV infection and the mechanisms underlying the antiviral effect of BIKE inhibitors.

We have recently demonstrated that AP2M1 is a substrate that is involved in mediating BIKE’s role in DENV infection (20). Nevertheless, no other functionally proven substrates of BIKE have been discovered. To better understand its role in DENV infection and cell signaling, we therefore sought to first identify its interactors and substrates. Mapping the interaction network of BIKE with the human proteome *via* BFG-Y2H screen and retrieving publicly available affinity purification mass spectrometry data (31) revealed 36 and 12 candidate BIKE interactors, respectively. CLINT1 emerged as a top hit in both screens. This degree of overlap is consistent with prior reports comparing hits generated by orthogonal platforms (54). CLINT1 and 21 other hits were further validated as BIKE interactors in mammalian cells, attesting to the high specificity of the screens.

Our results indicate that CLINT1 binds to and is phosphorylated by BIKE, is required for DENV assembly/egress, and is involved in mediating BIKE’s role in DENV infection. CLINT1 is a ∼68-kDa protein localized to the TGN and endosomal membranes (21, 26, 27). It is enriched in a subset of CCVs (26) and binds to PtdIns4*P* (21, 25), clathrin, AP1, and GGA2 (21, 27, 55). CLINT1 has been implicated in bidirectional vesicle trafficking in the retrograde (endosomes-to-TGN) (28, 56) and anterograde (TGN-to-endosomes) (21, 22) pathways. Nevertheless, the cellular mechanisms that regulate CLINT1 have not been reported. The discovery that BIKE phosphorylates CLINT1 provides insight into CLINT1 regulation. Moreover, we reveal a requirement for CLINT1 in a disease state, in which it was not previously implicated, beyond psychiatric disorders (57). Future studies will determine which role(s) of CLINT1 in cell biology and these other diseases is controlled by BIKE.

A large body of evidence supports that CLINT1 functions as a cargo-specific adapter rather than a general CCV adapter. First, CLINT1 has been shown to specifically interact with and sort several SNAREs implicated in membrane fusion, including vti1b (21, 22, 23), and syntaxin 7 and 8 (24) into CCVs. Furthermore, knockdown of CLINT1 (with the same siRNA used in this study) demonstrated no effect on the TGN localization of mannose-6-phosphate and CD8-furin, two cargo proteins that rely on CCVs to traffic between the TGN and endosomes (22). We provide evidence that CLINT1 is not required for DENV entry (which is *via* clathrin-mediated endocytosis) and that it specifically interacts with the DENV NS3 protein and cotraffics with individual viral particles. Combined, these data support that the role of CLINT1 in DENV infection does not result from a global effect on CCV production and/or trafficking.

Our mass spectrometry data indicate that CLINT1 undergoes phosphorylation by BIKE at T294, which is within an unstructured region of the protein that is accessible for interactions with cytoplasmic proteins (21). We provide evidence that this modification is biologically relevant *via* cell-based Phos-tag gel analysis showing a ∼3-fold decrease in the phosphorylation of CLINT1 T294 mutant relative to WT CLINT1. We, however, cannot exclude the possibility that additional CLINT1 residues are phosphorylated. Indeed, we identified three other putative phosphorylation residues (S205, S210, and S215), albeit under lower stringency analysis conditions and at a lower frequency (<1%).

We provide evidence that CLINT1 and its T294 residue are involved in mediating BIKE’s role in DENV infection. Ectopic expression of CLINT1 WT in the BIKE^KO^ cells restores DENV infection (Fig. 5*I*), yet it does not increase DENV infection as measured in the presence of BIKE in WT cells (Fig. 5*E*). This finding functionally links the roles of BIKE and CLINT1 in DENV infection and suggests that CLINT1’s function in DENV infection is, at least in part, regulated by BIKE. Yet, this finding also suggests that in the absence of BIKE, some function of CLINT1 is independent of BIKE regulation. The latter may be compensated for by another kinase and is in agreement with the detection of minor, yet residual, CLINT1 phosphorylation in the BIKE^KO^ cell line (Fig. S2). Moreover, while the T294 CLINT1 residue may be phosphorylated by another kinase in the absence of BIKE or mediate a phosphorylation-independent role, it is absolutely required to mediate the function of CLINT1 in DENV infection, as indicated by the failure of the CLINT1 T294A mutant to reverse the effect of CLINT1 (Fig. 5*G*) or BIKE (Fig. 5*I*) deletion on DENV infection.

The discovery that BIKE phosphorylates CLINT1 transforms our understanding of the mechanism that BIKE mediates in intracellular membrane trafficking: we reveal regulation at the TGN/endosomal pathway, beyond BIKE’s predicted role in clathrin-mediated endocytosis (6, 12, 13). This mechanism is further supported by the finding that BIKE is required for late stages of the DENV life cycle, in addition to viral entry. We previously showed that AP2M1 is another substrate whose phosphorylation is involved in mediating BIKE’s role in DENV infection and that AP2M1 is required for DENV entry (in addition to infectious virus production) (14, 15, 16, 20). Collectively, our findings thus suggest that BIKE mediates DENV entry in part *via* AP2M1 phosphorylation and DENV assembly/egress *via* CLINT1 and AP2M1 phosphorylation.

By demonstrating control of DENV assembly/egress by BIKE signaling in part *via* CLINT1 phosphorylation and CLINT1 cotrafficking with individual viral particles in live cells, we reveal a direct role for CLINT1 in DENV infection. We provide evidence that CLINT1 binds DENV NS3 and mediates DENV assembly/egress. Beyond its roles in DENV RNA replication and evasion of immune responses (58, 59), NS3 was shown to be required for infectious particle production and was proposed as a platform to recruit cellular factors required for assembly (60). A requirement for NS3 in assembly was similarly reported in yellow fever *Flavivirus* infection (60, 61). It is possible that CLINT1 is recruited from endosomes or TGN by NS3 to sites of assembly, which are predicted to be on ER-derived membranes (4). Alternatively, this may suggest presence of endosomal components in assembly sites. Contacts between endosomes and ER membranes may help facilitate CLINT1 recruitment to ER membranes (62). The findings that phosphorylated CLINT1 T294 is required for NS3 binding and for mediating BIKE’s role in DENV infection reveals one mechanism by which BIKE may regulate CLINT1’s function in DENV infection. Probing the role of T294 in CLINT1 cotrafficking with DENV particles is an interesting topic for future research. It is possible that DENV hijacks CLINT1 to enrich viral assembly sites with a subset of CCVs. Alternatively, CLINT1 may sort NS3 to assembly sites similarly to sorting its cellular cargo proteins, SNAREs. It is tempting to speculate that CLINT1 binding to the relevant SNAREs is also regulated *via* BIKE-mediated T294 phosphorylation.

We developed an advanced imaging approach to monitor DENV capsid trafficking in live cells. Prior studies in DENV have focused only on monitoring entry of DIL (1,1'-Dioctadecyl-3,3,3',3'-Tetramethylindocarbocyanine Perchlorate)- or DID (1,1-Dioctadecyl-3,3,3,3-tetramethylindodicarbocyanine)-labeled viral particles or individual viral proteins *via* live-cell imaging approaches (63, 64). To date, the tracking of individual DENV capsid puncta at late stages of the viral life cycle has not been possible. The establishment of this system thus provides a useful resource for the study of intracellular DENV trafficking in general. Using this approach, we show that CLINT1 cotraffics with DENV capsid. These findings exclude a theory whereby CLINT1 contributes to viral infection solely by recruiting or mediating intracellular traffic of host cargo components essential for the DENV life cycle. Given CLINT1’s roles in TGN/endosomal trafficking (21, 26, 27) and DENV assembly/egress, we predict that TC-capsid cotraffics with CLINT1 in post-TGN pathways. Other TGN/endosomal trafficking pathway components including RAB7L1 and GNB2L1 were previously implicated in DENV assembly/egress (26, 65). We have previously shown that AP1 is involved in mediating DENV infectious virus production. Since CLINT1 binds AP1 (21) and its budding was suggested to be AP1/clathrin-dependent (21), it is possible that both adapters are present in vesicles that transport virions. Nevertheless, CLINT1 has also been identified on more peripheral vesicles that are not stained for AP1 or clathrin (26), proposing that it may remain associated with the vesicles longer than these factors. Alternatively, CLINT1 may mediate trafficking in another pathway independently of AP1 and clathrin. For example, retrograde sorting in early/recycling endosomes, in which CLINT1 is implicated, was shown to be AP1 independent (28).

Our proteomic screens revealed additional hits implicated in intracellular membrane trafficking. These include proteins involved in endocytosis such as RABL2A (37), the two subunits of the AP2 complex, and REPS1 (66). Factors involved in trafficking in other pathways were also discovered including STAMBP (AMSH) that is involved in lysosomal trafficking of transmembrane receptors (67, 68), DYNLT3 that mediates apical transport in polarized epithelia (36), RUNDC3A that binds AP1M1 and may be involved in cytoskeletal rearrangements (69), and PICALM that is colocalized with and enhances TGN clathrin accumulation (70). It remains to be determined whether REPS1 and PICALM bind BIKE weakly or transiently like AP subunits (71) or represent nonspecific hits since these interactions did not reach the threshold for positivity in our PCA experiments. Regardless, these collective findings support regulation of various trafficking pathways and functions by BIKE.

Among the validated BIKE interactors were also proteins involved in host immune responses (ACAA1 (72) and RABL2A (73)), host stress response (FAM120A (74) and DAZAP1 (74)), the ubiquitin proteasome system (STAMBP and MYLIP (38)), transcription (MTA1 (39), PKNOX2 (75)), translation (EIF4E3 (26, 40)), virus-induced oncogenesis (MTA1 (43)), metabolic pathways (ACAA1 (76)), gene regulation (DAZAP1 (41), FAM120A (77)), cell proliferation and differentiation (DAZAP1 (78)), and male infertility (RABL2A, DAZAP1 (41)). Our phosphoproteomics analysis on a subset of these interactors revealed RABL2A, ACAA1, DAZAP1, MYLIP1, RUNDC3A, and FAM120A as putative substrates of BIKE beyond CLINT1 and AP2M1. Future work is required to validate these factors as BIKE substrates and identify the modification sites. Discovering these downstream effectors of BIKE fills a gap in our understanding of kinase biology and provides insight into the mechanisms by which BIKE regulates both cellular processes, including trafficking (12, 13) and osteoblast differentiation (12), and disease states (12, 51, 52, 53) that it was previously implicated in. Moreover, these findings propose signaling in pathways not previously reported for BIKE. For example, FAM120A and DAZAP1 are components of stress granules (74), suggesting that BIKE may be involved in oxidative stress–induced survival signaling.

These findings propose additional substrates beyond CLINT1 and AP2M1 that may mediate BIKE’s role in viral infections, some of which have been previously linked to viral infections. RABL2A was implicated in endocytosis of rotavirus (37). FAM120A is a scaffold protein required for activation of PI3-kinase in the PI3K/Akt (77) pathway, which has been shown to promote DENV infection (79). Since some of the putative substrates are understudied, their role in BIKE-regulated DENV infection may relate to heretofore undiscovered functions. We cannot exclude involvement of additional, yet to be identified, BIKE substrates, *e.g.*, those involved in autophagy, another cellular process required for DENV infection in which BIKE is implicated (80). The model we propose is that by phosphorylating various substrates that function as proviral factors, BIKE regulates temporally distinct step(s) in the DENV life cycle. Moreover, compounds with potent anti-BIKE activity, which we have recently shown to effectively suppress replication of DENV and other RNA viruses, likely elicit their antiviral effect *via* inhibition of several such BIKE substrates (20).

In summary, these findings validate virus–host interactions involved in mediating DENV entry and/or assembly/release and reveal cellular substrates of BIKE and their roles in DENV infection. These findings have implications for the roles of BIKE and its substrate CLINT1 in cellular processes and other disease states and for the design of host-targeted antiviral therapeutics.

## Experimental procedures

### Cell lines

Huh7, Huh7.5 (Apath LLC), 293T BHK-21 (ATCC), and Vero (ATCC) cells were grown in Dulbecco’s modified Eagle’s medium (DMEM) (Mediatech) supplemented with 10% fetal bovine serum (Omega Scientific), nonessential amino acids, 1% l-glutamine, and 1% penicillin–streptomycin (Gibco) and maintained in a humidified incubator with 5% CO_2_ at 37 °C.

Plasmids, antibodies, RNAi, and primers are summarized in Tables S3 and S4 in the supplemental material.

### Plasmids and virus constructs

ORFs encoding host proteins identified by the UHTY2H and BioPlex screens were selected from the Human ORFeome library of cDNA clones (Open biosystems) (81) and recombined into pGLuc, pFLAG, or pCherry vectors by Gateway technology (Invitrogen). DENV2 (New Guinea C strain) TSV01 Renilla reporter plasmid (pACYC NGC FL) was a gift from Pei-Yong Shi (University of Texas Medical Branch) (46), and DENV 16681 plasmid (pD2IC-30P-NBX) was a gift from Claire Huang (Centers for Disease Control and Prevention, Public Health Service, US Department of Health and Human Services) (48). pCMV-DV2Rep was a gift from Andrew Yueh (Institute of Biotechnology and Pharmaceutical Research) (47). Plasmids encoding the kinase domains of NAKs with N-terminal tobacco etch virus–cleavable His_6_ tags were a gift from Dr Stefan Knapp at the University of Oxford (6). Plasmids encoding the CLINT1-C (pTrcHisA-epsinR (1–165)) and CLINT1-N (pTrcHisA-epsinR (165–625)) fragments were a gift from Dr Jennifer Hirst (the University of Cambridge) (25). Mutations were introduced by site-directed mutagenesis using the QuikChange kit (Stratagene). Primer sequences not included in Table S4 will be provided upon request.

### TC-capsid construction

The TC-containing fragment was first synthesized by overlapping PCR using primer pairs PY-D2-1/F and PY+FLN-TC_163/R, as well as PY+FLN-TC_198/F and PY_LucDen2/SphI/R (Table S4). Backbone was prepared by linearizing pACYC-DENV2-NGC with SalI and SphI (NEB). TC-tagged DENV2 was assembled through yeast homologous recombination by cotransforming the TC-containing fragment and linearized backbone into yeast cells as described previously (82). The resulting infectious clone was purified from yeast cell and propagated in OverExpress C41 (DE3) chemically competent cells (Sigma-Aldrich).

### Western blotting and antibodies

Cells were lysed in M-Per protein extraction reagent (Thermo Fisher Scientific). Clarified protein lysates were run on 4%–12% Bis-Tris gels (Invitrogen) and transferred onto polyvinylidene fluoride membranes (Bio-Rad). Blots were blocked and blotted with anti-FLAG (Sigma, F7425), anti-CLINT1 (Bethyl, catalog A301–926A), anti-BIKE (Santa Cruz biotechnology, catalog sc-134284), anti-GLuc (New England BioLabs, catalog E8023S), and anti–β-actin (Sigma-Aldrich, catalog A3854) antibodies. Signal was detected with horseradish peroxidase–conjugated secondary antibodies. Band intensity was quantified with ImageJ software (NIH).

### RNA interfering

siGENOME Human BMP2K (55589) siRNA SMART Pool, siGENOME Non-Targeting siRNA Pool #1 (D-001206–13–05), CLINT1 (EpsinR) siRNA (ONtarget siRNA, QTE-2568052G), and ONtarget NT siRNA (D-001810–10–05) were purchased from Dharmacon. siRNAs (1 pmole) were transfected into Huh7 cells using RNAiMax (ThermoFisher Scientific) 72 h before infection (see Table S4 for gene and siRNA sequence details).

### Generation of BIKE and CLINT1 KO cell lines using CRISPR/Cas9

Guide RNA (gRNA) sequences were designed using the CRISPR design tool (http://chopchop.cbu.uib.no/). BIKE (GGTGGCGGCCGACCGCGAAC) and CLINT1(GACAAA GCGTGAGTATCGGGGGG) gRNAs were synthesized and cloned into the pX458 gRNA plasmid gifted by Dr Feng Zhang (Addgene plasmid # 48138), according to previously described protocols (83). Single clonal knockouts of Huh7 cells were obtained using the PX458 vector that expressed Cas9 and gRNAs against either BIKE or CLINT1. Green fluorescent protein (GFP)–positive single cells were sorted into 96-well plates 24 h post-transfection using a BD InFlux Cell Sorter. Finally, the cells were screened for knockout *via* Western blot, as described before (83).

### Barcoded Fusion Genetics-Yeast 2-Hybrid

BFG-Y2H was conducted as reported previously (29). Briefly, barcoded BIKE and individual prey ORFs were first constructed *via* assembly in yeast. Y2H was carried out by mating yeast cells containing barcoded BIKE plasmids with yeast cells containing barcoded prey ORF plasmids on YPAD plates, followed by selection of positive clones in synthetic complete media. Positive cells were then pooled, and Cre recombinase expression was induced by doxycycline to promote recombination between barcodes on BIKE and prey plasmids. Barcode regions on the plasmids purified from selected yeast cells were amplified by PCR and subject to next-generation sequencing. Protein–protein interactions were determined by analyzing linkages between barcodes from the BIKE and prey plasmids.

### Protein-fragment complementation assays

As described, combinations of plasmids encoding prey (A) and bait (B) proteins, each fused to a fragment of the *Gaussia* luciferase protein (GLuc1 and GLuc2) or control vectors, were cotransfected into 293T cells plated in 96-well plates in triplicate (33). At 24 h post-transfection, cells were lysed and subjected to luciferase assays (Promega). Results were expressed as NLRs calculated as follows: the average signal in cells transfected with GLuc1-A and GLuc2-B was divided by the average signal in wells transfected with GLuc1-A and an empty GLuc2 vector and those transfected with GLuc2-B and an empty GLuc1 vector.

### Phos-tag gel

Samples were prepared by lysing Huh7 cells in EDTA-free RIPA buffer (150 mM NaCl, 50 mM Tris, pH 8, 1% NP-40, 0.5% sodium deoxycholate, 0.1% SDS in dH_2_O) with 1× HALT protease inhibitor for 30 minutes in 4 °C. Samples were then centrifuged for 10 min at 12,000*g*, and the supernatant was transferred to a clean tube. Cell lysates were mixed with 4× EDTA-free SDS/PAGE sample buffer (BioRad) and 10× Bolt sample reducing agent. Ten to twenty micrograms of samples was added to each well of a Phos-tag gel (SuperSep Phos-tag [50 μmol/l], 7.5%, 17-well, 100 × 100 × 6.6 mm) and electrophoresed at 150 V for 2 h. For immunoblot analysis, gels were washed for 20 min in 1× BioRad transfer buffer containing 20% (v/v) methanol and 10 mM EDTA three times, followed by one 10-min wash in the transfer buffer containing 20% (v/v) methanol. The signals of the phosphorylated (P) and unphosphorylated (U) bands were quantified using ImageJ.

### Protein expression and purification

Plasmids encoding N-terminally His_6_-tagged kinase domains of individual NAKs or GST-tagged CLINT1(N), CLINT1(C) were electroporated into Rosetta strain BL21 *E. coli* cells using Gene Pulser Xcell Electroporation Systems (Bio-Rad) with 1800V. Protein expression was induced with 0.5 mM IPTG at 20 °C overnight. Cells were then harvested and resuspended in lysis buffer composed of 50 mM Hepes (pH 7.5), 500 mM NaCl, 5 mM imidazole, 5% glycerol, and 0.5 mM TCEP [tris-(2-carboxyethyl) phosphine]. Following sonication and spinning at 48,400*g* at 4 °C for 60 min, the supernatant was harvested. NAKs were purified *via* Ni-affinity followed by tobacco etch virus protease digestion to remove the HIS_6_ tag. NAKs were further purified by size-exclusion chromatography *via* Superdex S75 10/60 column on AKTA pure (GE Lifesciences). Proteins were concentrated *via* 10-kDa Amicon centrifugal filters (Merck) and suspended in storage buffer composed of 10 mM Hepes (pH 7.5), 300 mM NaCl, 5% glycerol, and 0.5 mM TCEP at −80 °C. Clint1(N) and Clint1(C) were purified by glutathione sepharose beads and eluted with 10 mM glutathione. His_6_-tagged recombinant RbC (aa 771-928) was purified by cobalt–sepharose affinity chromatography (GE Sepharose cat#17-0575-01) and eluted with 200 mM imidazole. Histone H1 protein was from EMD Millipore (cat#14-155).

### *In vitro* kinase assay

For radioactive assays, 1 to 5 μM recombinant substrate was mixed with 100 nM kinase, 50 mM Hepes (pH7.4), 150 mM NaCl, 5 mM MgCl_2_, 20 mM imidazole (with RbC only), 0.2 mg/ml BSA, and 500 μM ATP (with 2 μCi of [γ-32P] ATP [PerkinElmer cat# BLU502Z250UC]). Aliquots were collected at 30 and 60 min, and the reaction was stopped with SDS-PAGE sample buffer. Phosphorylation was visualized using autoradiography (Typhoon instrument; GE Healthcare) (84). The same mixture was used for LC-MS/MS analysis, with or without the addition of ATP to the reaction. Phosphorylation of AP2M1 peptide (SQITSQVTGQIGWRREG, Genscript) was determined as described previously (6). For phosphorylation site determination, 1 mM rCLINT1(C) was incubated with 1 μM rBIKE in assay buffer with or without 500 μM ATP for 60 min. Reaction was stopped by adding 10 volumes of cold acetone and freezing at –80 °C before trypsin and chymotrypsin digestion.

### Co-immunoprecipitations

Huh7 cells were transfected with plasmids encoding FLAG-tagged proteins and lysed after 48 h using 50 mM Tris HCl, pH 7.4, with 150 mM NaCl, 1 mM EDTA, and 1% TRITON X-100. HALT protease inhibitor cocktails (100×) were added, and samples were incubated for 30 min at 4 °C on a shaker. Samples were then spun down for 10 min at 12,000*g*. Two hundred microliters of each supernatant in duplicate was then transferred to a tube containing Mouse IgG Agarose (Sigma) prewashed with Tris-buffered saline with 0.1% Tween 20 (TBST), and incubated at 4 °C for at least 2 h. One duplicate of each sample was spun down, and the supernatant was transferred to a tube containing prewashed Anti-FLAG affinity gel (Bimake), while the second duplicate remained in Mouse IgG Agarose as a negative control. All samples were incubated overnight at 4 °C, washed, and recovered in protein sample buffer.

### Virus production

DENV RNA was transcribed *in vitro* from pACYC-DENV2-NGC plasmid using mMessage/mMachine (Ambion) kits. The RNAs were electroporated into BHK-21 cells. The supernatant was harvested at day 10 and titrated by plaque assays on BHK-21 cells or was used to inoculate C6/36 cells to amplify the virus for production of virus stocks.

### Infection assays

Huh7 cells were seeded at 10^4^ cells/well of 96-well plates. Twenty-four hours later, the cells were infected with DENV expressing a luciferase reporter in 3 to 10 replicates at an multiplicity of infection of 0.05. Overall infection was measured at 48 or 72 h postinfection using a *Renilla* luciferase substrate. Luminescence was read immediately by an InfiniteM1000 plate reader (Tecan) or a SpectraMax 340PC.

### Entry assays

As described earlier, Huh7 cells were infected with DENV2 expressing a luciferase reporter and the activity of *Renilla* luciferase was measured 6 h postinfection.

### Viability assays

Viability was assessed using alamarBlue reagent (Invitrogen) or CellTiter-Glo reagent (Promega) assay according to the manufacturer’s protocol. Fluorescence was detected at 560 nm on an InfiniteM1000 plate reader.

### DENV RNA replication

As described (14), Huh7 were transfected with DNA-launched DENV2 replicon (pCMV-DV2Rep) and TET-ON plasmids. Eighteen hours post-transfection, viral RNA transcription was induced by doxycycline and inhibited by changing to doxycycline-free medium 24 h later. Replication was monitored daily by luciferase activity for 3 days.

### Extracellular and intracellular infectivity

Huh7 cells were seeded in 24-well plates and were transfected with 200 ng of DENV RNA and incubated for 48 h. To measure extracellular infectivity, cell culture supernatants were collected and used to infect naïve CLINT1-depleted or control Huh7 cells for 48 h. To measure intracellular DENV infectivity, lysates of transfected cells were subjected to three rounds of freeze-thawing using ethanol/dry ice and clarified at 5000*g*, as previously described (14). Luciferase or standard plaque assays were used to quantify titers of the infectious virus in the naïve cells.

### Gain-of-function assays

WT, T294A CLINT1, or empty vector controls were transfected into Huh7 cells. Twenty-four hours post-transfection, cells were infected with luciferase reporter DENV (Multiplicity of infection = 0.05) and incubated for 48 h prior to luciferase and viability assays as described earlier.

### Alanine scanning mutagenesis of DENV2 (16681) capsid

Alanine scanning of DENV2 (16681) capsid was conducted as previously described (Wu *et al*. 2015). Briefly, pTight-DENV2 was first digested with SacI and SphI. DNA fragments containing mutated capsid were synthesized by overlapping PCR with primer pair pTight/F and 16681/1434/R as well as corresponding overlapping mutagenesis primers (Table S4). DENV2 clones were assembled by cotransforming SacI/SphI digested pTight-DENV2 with individual capsid mutant fragments into yeast cells. The plasmids were propagated in *E. coli* (OverExpress C41 [DE3] chemically competent cells, Sigma Aldrich). To measure their infectivity, DNA of individual DENV capsid mutants and TET-OFF plasmids were cotransfected into HEK293 followed by immunofluorescence staining of the DENV envelope protein with anti-E antibody (2H2, ATCC).

### Mass spectrometry

Mass spectrometry was performed using an Orbitrap Q Exactive HF-X mass spectrometer (Thermo Scientific) with liquid chromatography performed on a Nanoacquity UPLC (Waters Corporation). For a typical LCMS experiment, a flow rate of 450 nl/min was used, where mobile phase A was 0.2% formic acid in water and mobile phase B was 0.2% formic acid in acetonitrile. Analytical columns were prepared in-house with an I.D. of 100 microns packed with Magic 1.8 micron C18 stationary phase (NanoLCMS Solutions) to a length of ∼25 cm. Peptides were directly injected onto the analytical column using a gradient (2–45% B, followed by a high-B wash) of 80 min. The mass spectrometer was operated in a data-dependent fashion using higher-energy C-trap dissociation fragmentation for MS/MS spectra generation.

For data analysis, the .RAW data files were checked using Preview (Protein Metrics) to verify success of the injection and sample quality. They were then processed using Byonic v3.3.11 (Protein Metrics) to identify peptides. Spectra were validated by hand using Byologic (Protein Metrics) for interpretation of MS1, MS2, and chromatographic XICs. Proteolysis with Trypsin/LysC was assumed to be fully specific with up to two missed cleavage sites. Precursor mass accuracies were held within 12 ppm with fragment ions also held within 12 ppm.

### Live-cell imaging

Huh7 cells were cotransfected with DENV TC-Capsid RNA and CLINT1-mCherry expression plasmid using Lipofectamine 2000. Seventy-two hours post-transfection, the cells were FlAsH stained using the Invitrogen TC-FlAsH II Kit (T34561). Cells were imaged on a Leica SP-5 Confocal Microscope over 58 frames across 3 min. The images were analyzed using the TrackMate plugin for Image J to identify discrete particles in each frame no more than 1.2 μm in diameter and then assign these particles into tracks across all the frames. Each track thus represents one particle of either CLINT1 or TC-Capsid moving across the cell over time. Static particles are those deemed to be the ones that moved less than 1 μm over at least 3 frames, and trafficking particles are those that moved more than 1 μm over at least 3 frames. Colocalization between TC-Capsid and CLINT1 was determined by comparing the coordinates of their respective particles. Particles that were one particle radius (0.6 μm or 2.5 pixels) or less distant from each other in the same frame were categorized as colocalizing, whereas those further apart or in different frames were deemed to not be colocalizing. Cotrafficking particles were those that colocalized together over at least 3 frames and moved over 1 μm during their period of colocalization.

### Quantification and statistical analyses

All data were analyzed with GraphPad Prism software (version 9.3.3). Half maximal effective concentrations (EC_50_) were measured by fitting of data to a 3-parameter logistic curve. *p* values were calculated by 1- or 2-way ANOVA with either Dunnett’s or Tukey’s multiple comparisons tests as specified in each figure legend. All software and programs used in this paper are available freely and are discussed in detail.

## Data availability

The raw mass spectrometry data were deposited under MSV000088630 in MassIVE database: https://massive.ucsd.edu/

## Supporting information

This article contains supporting information.

## Conflict of interest

The authors declare that they have no conflicts of interest with the contents of this article.
