# Supplementary material for: The cargo adapter protein CLINT1 is phosphorylated by the Numb-associated kinase BIKE and mediates dengue virus infection
Source: J Biol Chem. 2022 Apr 20;298(6):101956. doi: 10.1016/j.jbc.2022.101956 (PMC9133654; doi:10.1016/j.jbc.2022.101956)
Supplement: Supplementary Table S1 [file mmc2.docx]

| **Gene Symbol** |  |  |
| --- | --- | --- |
| **Prey** | **# of observations** | **Stickiness index %** |
| Total | 5631 |  |
| FHL3 | 2033 | 93 |
| PPP2R5A | 556 | 68 |
| PRKCH | 132 | 28 |
| PPP2R5B | 131 | 36 |
| SMYD1 | 120 | 21 |
| CPEB4 | 104 | 85 |
| PPP2R5E | 104 | 40 |
| CLINT1 | 100 | 9 |
| ZRANB3 | 82 | 13 |
| CNTFR | 76 | 44 |
| GBX2 | 69 | 79 |
| DAZAP1 | 54 | 9 |
| OR5B12 | 37 | 45 |
| UQCRB | 37 | 51 |
| DDX19A | 36 | 44 |
| TMEM87A | 34 | 33 |
| MYL6 | 33 | 17 |
| HAGH | 30 | 25 |
| BROX | 29 | 19 |
| CKAP2 | 27 | 23 |
| FEM1A | 26 | 76 |
| CCL27 | 16 | 13 |
| ZNF366 | 16 | 13 |
| ANKMY2 | 15 | 11 |
| PRIM1 | 15 | 15 |
| CPZ | 13 | 27 |
| PFN3 | 13 | 19 |
| TTC40 | 13 | 28 |
| WDR77 | 13 | 15 |
| CUL1 | 13 | 23 |
| DNASE1L1 | 12 | 21 |
| NYX | 12 | 27 |
| BAAT | 12 | 9 |
| HDHD2 | 12 | 19 |
| KCNJ10 | 12 | 29 |
| PCGF2 | 11 | 31 |
| RASSF2 | 11 | 23 |
| NOP10 | 11 | 35 |
| PKNOX2 | 11 | 9 |
| TMEM74B | 11 | 12 |
| STXBP6 | 11 | 5 |
| PCYT1B | 10 | 39 |
| GJD3 | 10 | 9 |
| IRGC | 10 | 13 |
| STEAP4 | 10 | 35 |
| CXorf56 | 10 | 39 |
| SRP9 | 10 | 23 |
| EPS8L1 | 10 | 32 |
| CXorf27 | 10 | 24 |
| KIAA0930 | 10 | 19 |
| MAN1C1 | 10 | 5 |
| CCM2 | 9 | 33 |
| UBE2M | 9 | 21 |
| SCGB1C1 | 9 | 9 |
| NRG2 | 9 | 4 |
| ACAA1 | 8 | 9 |
| C22orf15 | 8 | 13 |
| SNX20 | 8 | 51 |
| LAGE3 | 8 | 15 |
| ZNF175 | 8 | 28 |
| AKT2 | 8 | 31 |
| IFNA13 | 8 | 25 |
| TRAPPC6B | 8 | 9 |
| TRPV5 | 8 | 25 |
| HLA-DRA | 8 | 11 |
| NKX6-3 | 7 | 13 |
| SPINK13 | 7 | 11 |
| CIB1 | 7 | 12 |
| FAM192A | 7 | 47 |
| CX3CL1 | 7 | 37 |
| DPPA4 | 7 | 17 |
| LYSMD3 | 7 | 8 |
| CCDC42 | 7 | 11 |
| RAB17 | 7 | 17 |
| NAA15 | 7 | 9 |
| HMGB4 | 7 | 15 |
| RABIF | 7 | 15 |
| ITGB3BP | 7 | 29 |
| PAX9 | 7 | 25 |
| NPPA | 7 | 12 |
| CSAD | 7 | 7 |
| ANKRD2 | 6 | 11 |
| RPL13A | 6 | 32 |
| LIMCH1 | 6 | 11 |
| MAEL | 6 | 25 |
| CRCP | 6 | 11 |
| EIF1 | 6 | 19 |
| ZNRF3 | 6 | 65 |
| CNN2 | 6 | 56 |
| FAM125A | 6 | 44 |
| TAF12 | 6 | 16 |
| TTLL3 | 6 | 15 |
| C6 | 6 | 17 |
| MYLIP | 6 | 9 |
| SIGLEC7 | 6 | 9 |
| ASB14 | 6 | 15 |
| HIP1R | 6 | 15 |
| PMF1 | 6 | 35 |
| NFKBIL1 | 6 | 4 |
| SHISA3 | 6 | 9 |
| CRCT1 | 6 | 36 |
| USP32 | 5 | 20 |
| TAF8 | 5 | 37 |
| HHLA2 | 5 | 17 |
| ZNF148 | 5 | 23 |
| ING5 | 5 | 17 |
| ZNF576 | 5 | 13 |
| RGPD5 | 5 | 11 |
| GLT25D2 | 5 | 23 |
| TIAL1 | 5 | 36 |
| C1orf63 | 5 | 9 |
| RABL2A | 5 | 9 |
| GBP5 | 5 | 21 |
| NAA11 | 5 | 20 |
| TLR8 | 5 | 27 |
| MRPL34 | 5 | 36 |
| MRPL47 | 5 | 25 |
| BMP3 | 5 | 9 |
| ATM | 5 | 12 |
| FBXL14 | 4 | 15 |
| CIDEB | 4 | 43 |
| TMEM52 | 4 | 72 |
| GRP | 4 | 33 |
| C5orf55 | 4 | 20 |
| C19orf48 | 4 | 11 |
| MRPL53 | 4 | 11 |
| MAP2 | 4 | 48 |
| GLYR1 | 4 | 77 |
| PIN4 | 4 | 31 |
| C20orf96 | 4 | 19 |
| EIF4E2 | 4 | 32 |
| RBM6 | 4 | 37 |
| ZNF677 | 4 | 13 |
| PDYN | 4 | 15 |
| MLN | 4 | 24 |
| SIRT2 | 4 | 9 |
| SH2D4A | 4 | 9 |
| C16orf91 | 4 | 12 |
| DLD | 4 | 9 |
| LSM2 | 4 | 16 |
| DNAJC22 | 4 | 17 |
| YIPF3 | 4 | 15 |
| FAM171B | 4 | 19 |
| VGLL1 | 4 | 27 |
| EPO | 4 | 41 |
| ZDHHC22 | 4 | 5 |
| CXorf40A | 4 | 16 |
| NCKAP5L | 4 | 17 |
| KCNK18 | 4 | 13 |
| ZER1 | 4 | 3 |
| LYZL4 | 4 | 7 |
| SYF2 | 4 | 11 |
| KNCN | 3 | 20 |
| CDRT15 | 3 | 33 |
| C1orf189 | 3 | 16 |
| CCDC102B | 3 | 63 |
| ADRB3 | 3 | 20 |
| CDC42SE1 | 3 | 52 |
| RAP1GAP | 3 | 31 |
| RNASE3 | 3 | 24 |
| IMPACT | 3 | 11 |
| CDKN2AIPNL | 3 | 19 |
| PCDHA12 | 3 | 37 |
| TUFT1 | 3 | 31 |
| RGS4 | 3 | 43 |
| DCAF10 | 3 | 19 |
| KIAA1324L | 3 | 28 |
| CSNK2A2 | 3 | 16 |
| IGF1R | 3 | 29 |
| PCDH10 | 3 | 35 |
| RASGEF1A | 3 | 20 |
| ARPC3 | 3 | 11 |
| MEF2BNB | 3 | 27 |
| PPP1R27 | 3 | 49 |
| FAM92B | 3 | 16 |
| DUSP15 | 3 | 31 |
| ARL13B | 3 | 20 |
| EIF4E3 | 3 | 9 |
| MCF2 | 3 | 59 |
| NECAB1 | 3 | 8 |
| SUGT1 | 3 | 13 |
| ST6GAL1 | 3 | 9 |
| RHOQ | 3 | 24 |
| HRAS | 3 | 23 |
| CHTF8 | 3 | 20 |
| NKAP | 3 | 15 |
| SPRY4 | 3 | 15 |
| ATP5F1 | 3 | 32 |
| FAM120A | 3 | 9 |
| TGFBI | 3 | 12 |
| RSPO4 | 3 | 17 |
| STMN4 | 3 | 21 |
| HUNK | 3 | 49 |
| GPR162 | 3 | 11 |
| COL20A1 | 3 | 15 |
| FAIM | 3 | 16 |
| CYCS | 3 | 24 |
| PPIL3 | 3 | 17 |
| CYBA | 3 | 20 |
| HBB | 3 | 7 |
| P2RY8 | 3 | 39 |
| SYDE1 | 3 | 11 |
| CD244 | 3 | 23 |
| H2AFB3 | 3 | 28 |
| TCHHL1 | 3 | 27 |
| DYNLT3 | 3 | 7 |
| GNA12 | 3 | 24 |
| MTA1 | 3 | 5 |
| DGCR6 | 3 | 1 |
| ZNF468 | 3 | 8 |
| PRSS50 | 2 | 11 |
| FGF3 | 2 | 41 |
| FGFRL1 | 2 | 25 |
| ARMC8 | 2 | 12 |
| PPP1R32 | 2 | 28 |
| MDM1 | 2 | 25 |
| CTSL1 | 2 | 11 |
| ODF4 | 2 | 24 |
| NECAP1 | 2 | 21 |
| SNRPN | 2 | 20 |
| RABL5 | 2 | 21 |
| C21orf91 | 2 | 13 |
| COBRA1 | 2 | 15 |
| SYPL1 | 2 | 21 |
| PCDHB3 | 2 | 39 |
| RPS14 | 2 | 36 |
| CCL14 | 2 | 24 |
| CHPF | 2 | 32 |
| C17orf51 | 2 | 9 |
| RAB26 | 2 | 19 |
| LCE1D | 2 | 25 |
| ERCC3 | 2 | 11 |
| IQSEC2 | 2 | 36 |
| ADAM17 | 2 | 9 |
| SH2D1A | 2 | 15 |
| KRT1 | 2 | 19 |
| PPARA | 2 | 71 |
| H2AFB2 | 2 | 60 |
| DNAL4 | 2 | 9 |
| GNG12 | 2 | 17 |
| HIST1H4L | 2 | 65 |
| ZNF192 | 2 | 12 |
| C1QB | 2 | 12 |
| MTHFS | 2 | 19 |
| ZNF670 | 2 | 23 |
| IL20 | 2 | 11 |
| OR2L2 | 2 | 16 |
| CD3D | 2 | 31 |
| MSRB2 | 2 | 16 |
| AEBP2 | 2 | 60 |
| WFDC6 | 2 | 8 |
| GSTA2 | 2 | 16 |
| KATNAL2 | 2 | 9 |
| SRSF3 | 2 | 15 |
| CKS2 | 2 | 20 |
| ING2 | 2 | 9 |
| OR9G1 | 2 | 9 |
| SUGP1 | 2 | 11 |
| ERVFRD-1 | 2 | 8 |
| LZIC | 2 | 17 |
| SMC6 | 2 | 21 |
| MYBPHL | 2 | 29 |
| TMEM147 | 2 | 9 |
| KCNN1 | 2 | 17 |
| HIST1H2AL | 2 | 11 |
| ELL3 | 2 | 17 |
| RAB9A | 2 | 9 |
| C10orf53 | 2 | 8 |
| PPP1R21 | 2 | 28 |
| HAPLN2 | 2 | 16 |
| YWHAG | 2 | 19 |
| ZNF213 | 2 | 35 |
| FGF7 | 2 | 23 |
| PPP4R4 | 2 | 33 |
| GPR37 | 2 | 9 |
| DSCR6 | 2 | 23 |
| ZCCHC14 | 2 | 24 |
| XRCC2 | 2 | 13 |
| DBF4 | 2 | 40 |
| PIK3R5 | 2 | 25 |
| DIS3L2 | 2 | 33 |
| LSM6 | 2 | 15 |
| MIS12 | 2 | 13 |
| TNNI2 | 2 | 25 |
| TMOD3 | 2 | 17 |
| LMX1A | 2 | 31 |
| GRXCR1 | 2 | 43 |
| TTC19 | 2 | 12 |
| ATG14 | 2 | 16 |
| ARNTL | 2 | 11 |
| MBIP | 2 | 11 |
| NPW | 2 | 17 |
| SCML4 | 2 | 21 |
| VAV1 | 2 | 21 |
| SMOX | 2 | 9 |
| CDC42EP3 | 2 | 15 |
| NPDC1 | 2 | 20 |
| KLHL31 | 2 | 9 |
| CHD2 | 2 | 13 |
| LY6D | 2 | 16 |
| ERVW-1 | 2 | 17 |
| LINGO2 | 2 | 21 |
| ZC3H11A | 2 | 73 |
| POLR2A | 2 | 9 |
| PPM1K | 2 | 37 |
| SENP2 | 2 | 24 |
| PTDSS1 | 2 | 8 |
| UBE2E2 | 2 | 21 |
| SENP3 | 2 | 29 |
| ANXA9 | 2 | 8 |
| OR5L2 | 2 | 8 |
| FAM83A | 2 | 9 |
| HIST1H2BD | 2 | 11 |
| HIST2H2BF | 2 | 19 |
| DNAJB13 | 2 | 25 |
| TTLL7 | 2 | 19 |
| BPY2B | 2 | 17 |
| YBEY | 2 | 7 |
| TRMT112 | 2 | 8 |
| SPDYE2 | 2 | 13 |
| ARL16 | 2 | 16 |
| TTC30B | 2 | 19 |
| NLN | 2 | 19 |
| DLX3 | 2 | 19 |
| KIAA1967 | 2 | 12 |
| NDUFA10 | 2 | 5 |
| IGFLR1 | 2 | 24 |
| SCNN1B | 2 | 7 |
| ECI2 | 2 | 12 |
| C10orf120 | 2 | 11 |
| RPS18 | 2 | 12 |
| ALKBH1 | 2 | 19 |
| FIGF | 2 | 9 |
| FYTTD1 | 2 | 9 |
| KRTAP19-2 | 2 | 19 |
| KLK5 | 2 | 24 |
| CCL25 | 2 | 15 |
| FAM104B | 2 | 16 |
| HSPA14 | 2 | 7 |
| C20orf195 | 2 | 13 |
| MYCL1 | 2 | 15 |
| WNT8B | 1 | 12 |
| C11orf67 | 1 | 29 |
| SERTAD4 | 1 | 57 |
| EML1 | 1 | 13 |
| PLAC9 | 1 | 13 |
| ADAMTS17 | 1 | 19 |
| C3orf36 | 1 | 9 |
| AQP7 | 1 | 23 |
| SLC27A3 | 1 | 32 |
| CCDC84 | 1 | 23 |
| FAM204A | 1 | 23 |
| C17orf82 | 1 | 17 |
| FAM46B | 1 | 29 |
| MRM1 | 1 | 9 |
| HDAC9 | 1 | 9 |
| POP4 | 1 | 43 |
| HADHB | 1 | 25 |
| DBX2 | 1 | 11 |
| CCNG1 | 1 | 44 |
| COMMD1 | 1 | 13 |
| COMMD10 | 1 | 16 |
| BAGE5 | 1 | 17 |
| PRUNE2 | 1 | 20 |
| LYZL2 | 1 | 19 |
| FBXO38 | 1 | 12 |
| SCGB3A1 | 1 | 32 |
| LGI2 | 1 | 11 |
| DEFB116 | 1 | 19 |
| SLC9A3R1 | 1 | 11 |
| VAX1 | 1 | 13 |
| ZNF169 | 1 | 27 |
| TRIM56 | 1 | 8 |
| CDC20 | 1 | 27 |
| KDELR2 | 1 | 16 |
| VPS29 | 1 | 16 |
| ARL6 | 1 | 33 |
| GLRX2 | 1 | 8 |
| TRPV6 | 1 | 20 |
| PNOC | 1 | 25 |
| TKT | 1 | 40 |
| SGPP2 | 1 | 19 |
| PGK1 | 1 | 12 |
| LIN37 | 1 | 43 |
| ARL11 | 1 | 17 |
| MZT2B | 1 | 23 |
| POLR2L | 1 | 21 |
| LCE3A | 1 | 28 |
| TACSTD2 | 1 | 8 |
| TXNL4B | 1 | 15 |
| TSEN15 | 1 | 9 |
| VILL | 1 | 24 |
| PRPS2 | 1 | 16 |
| C2orf48 | 1 | 23 |
| TSPY3 | 1 | 29 |
| FBXO34 | 1 | 8 |
| CLCF1 | 1 | 11 |
| PDHX | 1 | 12 |
| ANGEL2 | 1 | 21 |
| KRT79 | 1 | 12 |
| CIR1 | 1 | 29 |
| MYF6 | 1 | 48 |
| ZNF257 | 1 | 15 |
| PIN1 | 1 | 33 |
| FAM107A | 1 | 53 |
| PCNP | 1 | 9 |
| SHOX | 1 | 61 |
| DEFB103A | 1 | 8 |
| RPS7 | 1 | 24 |
| RPUSD1 | 1 | 29 |
| RDM1 | 1 | 49 |
| BAD | 1 | 15 |
| CLLU1OS | 1 | 23 |
| OR1J1 | 1 | 24 |
| BBS10 | 1 | 40 |
| CHAF1B | 1 | 9 |
| GTSF1L | 1 | 61 |
| PDCD5 | 1 | 9 |
| C10orf25 | 1 | 19 |
| LEFTY2 | 1 | 20 |
| CAPZB | 1 | 13 |
| PHLDA3 | 1 | 16 |
| SIGLEC11 | 1 | 35 |
| CDADC1 | 1 | 19 |
| RARB | 1 | 51 |
| FBXL3 | 1 | 9 |
| ASB2 | 1 | 23 |
| GAGE12E | 1 | 31 |
| RASD1 | 1 | 15 |
| TBC1D3F | 1 | 19 |
| LHB | 1 | 24 |
| FAM64A | 1 | 37 |
| IER3IP1 | 1 | 41 |
| GP1BB | 1 | 49 |
| HSF1 | 1 | 8 |
| KLK14 | 1 | 9 |
| DDX47 | 1 | 11 |
| C9orf91 | 1 | 28 |
| POM121C | 1 | 39 |
| CDC14A | 1 | 13 |
| KIF1C | 1 | 11 |
| TSKS | 1 | 16 |
| DEDD2 | 1 | 20 |
| POU6F1 | 1 | 12 |
| NODAL | 1 | 21 |
| TLR10 | 1 | 11 |
| RD3 | 1 | 23 |
| NIP7 | 1 | 11 |
| POLI | 1 | 12 |
| EIF3K | 1 | 23 |
| AIFM1 | 1 | 28 |
| AP3D1 | 1 | 9 |
| REP15 | 1 | 12 |
| FDXR | 1 | 20 |
| BARX1 | 1 | 23 |
| HAL | 1 | 28 |
| ZNF193 | 1 | 33 |
| HIST1H2AH | 1 | 37 |
| GEM | 1 | 15 |
| CD59 | 1 | 24 |
| KISS1 | 1 | 25 |
| TMSB4Y | 1 | 31 |
| COX4I2 | 1 | 9 |
| PITRM1 | 1 | 12 |
| ENSA | 1 | 21 |
| ALG12 | 1 | 24 |
| IL10 | 1 | 40 |
| TEX26 | 1 | 5 |
| ZNF431 | 1 | 16 |
| PRDM5 | 1 | 19 |
| KRT6A | 1 | 20 |
| ZDHHC7 | 1 | 29 |
| B3GNT3 | 1 | 9 |
| HINT2 | 1 | 11 |
| ICK | 1 | 11 |
| LRRFIP1 | 1 | 13 |
| MYO19 | 1 | 15 |
| IL12RB1 | 1 | 9 |
| RPUSD4 | 1 | 11 |
| GDPD5 | 1 | 16 |
| TCEAL2 | 1 | 25 |
| MLKL | 1 | 28 |
| BEX2 | 1 | 29 |
| THEMIS | 1 | 16 |
| GOLGA7 | 1 | 21 |
| ZNF434 | 1 | 25 |
| FDX1 | 1 | 43 |
| NAT14 | 1 | 8 |
| SCTR | 1 | 9 |
| B3GALT2 | 1 | 12 |
| COX17 | 1 | 19 |
| MB | 1 | 23 |
| KIAA1279 | 1 | 45 |
| FAM126A | 1 | 56 |
| C9orf40 | 1 | 9 |
| KIAA1598 | 1 | 9 |
| GPRIN3 | 1 | 11 |
| DCUN1D4 | 1 | 13 |
| KREMEN2 | 1 | 16 |
| ANGPT1 | 1 | 23 |
| FAU | 1 | 37 |
| FAM90A1 | 1 | 39 |
| SPSB2 | 1 | 9 |
| XAGE1A | 1 | 15 |
| EFCAB11 | 1 | 16 |
| MLF1IP | 1 | 16 |
| ZC3H14 | 1 | 17 |
| APOBEC1 | 1 | 37 |
| DHX40 | 1 | 8 |
| ESRRB | 1 | 13 |
| R3HCC1L | 1 | 16 |
| ZNF324B | 1 | 16 |
| CNTN5 | 1 | 19 |
| SPATS1 | 1 | 33 |
| CDC25A | 1 | 9 |
| EIF2AK4 | 1 | 9 |
| KCNH7 | 1 | 11 |
| PGC | 1 | 13 |
| NADKD1 | 1 | 19 |
| XRCC6BP1 | 1 | 39 |
| SMYD4 | 1 | 13 |
| XDH | 1 | 15 |
| FBL | 1 | 20 |
| RBM10 | 1 | 20 |
| PKD2L2 | 1 | 27 |
| GABRR2 | 1 | 7 |
| ZNF816 | 1 | 9 |
| ELL2 | 1 | 12 |
| APOA1BP | 1 | 16 |
| CHRM5 | 1 | 17 |
| QTRT1 | 1 | 17 |
| EDA2R | 1 | 21 |
| DUX5 | 1 | 28 |
| KRR1 | 1 | 32 |
| SUPT4H1 | 1 | 7 |
| DAZL | 1 | 15 |
| ADAM7 | 1 | 9 |
| GPR158 | 1 | 13 |
| CHSY3 | 1 | 15 |
| MYLK2 | 1 | 20 |
| OR6C65 | 1 | 20 |
| ASB7 | 1 | 31 |
| B4GALT3 | 1 | 35 |
| TGS1 | 1 | 8 |
| TONSL | 1 | 9 |
| ARL17B | 1 | 20 |
| LCE2C | 1 | 24 |
| MTX1 | 1 | 8 |
| GEMIN6 | 1 | 9 |
| LITAF | 1 | 9 |
| NAAA | 1 | 9 |
| EHD3 | 1 | 12 |
| SLC6A19 | 1 | 16 |
| CST5 | 1 | 17 |
| C20orf197 | 1 | 19 |
| FAM195A | 1 | 25 |
| DHRS7 | 1 | 28 |
| ERGIC3 | 1 | 35 |
| PSMD12 | 1 | 8 |
| NAT16 | 1 | 11 |
| ZNF843 | 1 | 13 |
| ACO2 | 1 | 16 |
| GTSE1 | 1 | 21 |
| CCDC112 | 1 | 25 |
| ATP5J | 1 | 28 |
| DDX60 | 1 | 7 |
| HLA-DQA1 | 1 | 9 |
| CEACAM3 | 1 | 12 |
| CALCB | 1 | 15 |
| OR10K1 | 1 | 17 |
| TOM1L2 | 1 | 31 |
| ASCC3 | 1 | 7 |
| TUBE1 | 1 | 9 |
| SNCA | 1 | 23 |
| NEUROD4 | 1 | 24 |
| CNOT4 | 1 | 28 |
| DZIP1L | 1 | 39 |
| HIRA | 1 | 4 |
| DHDDS | 1 | 8 |
| PCDH9 | 1 | 12 |
| TAS2R20 | 1 | 12 |
| DESI1 | 1 | 13 |
| IL23A | 1 | 13 |
| NET1 | 1 | 21 |
| SPSB3 | 1 | 27 |
| CDC20B | 1 | 49 |
| MAFG | 1 | 53 |
| CHTF18 | 1 | 7 |
| NDUFA7 | 1 | 7 |
| BCL2L12 | 1 | 8 |
| RAB39A | 1 | 8 |
| CST9L | 1 | 9 |
| ITPRIPL2 | 1 | 9 |
| SLC25A25 | 1 | 9 |
| CASP5 | 1 | 11 |
| FEN1 | 1 | 16 |
| ARFGAP1 | 1 | 17 |
| NPTN | 1 | 21 |
| TXNDC15 | 1 | 24 |
| HESX1 | 1 | 63 |
| MECOM | 1 | 8 |
| BST1 | 1 | 9 |
| COLQ | 1 | 11 |
| HINT3 | 1 | 11 |
| EXOC4 | 1 | 15 |
| NPHP4 | 1 | 17 |
| IL20RB | 1 | 19 |
| PTGER3 | 1 | 27 |
| TAS2R4 | 1 | 32 |
| MYLK4 | 1 | 7 |
| DHRS3 | 1 | 11 |
| SFRP4 | 1 | 16 |
| TMEM129 | 1 | 17 |
| C17orf80 | 1 | 23 |
| MRPL28 | 1 | 24 |
| IFNG | 1 | 25 |
| SPHK1 | 1 | 4 |
| BOLA2 | 1 | 5 |
| B4GALT7 | 1 | 7 |
| GABRA6 | 1 | 11 |
| ACSL6 | 1 | 16 |
| MFI2 | 1 | 17 |
| S100A1 | 1 | 19 |
| ATP5S | 1 | 21 |
| DTWD1 | 1 | 21 |
| HMGN4 | 1 | 23 |
| DLEU7 | 1 | 7 |
| PAG1 | 1 | 8 |
| ST6GALNAC6 | 1 | 9 |
| DRG2 | 1 | 11 |
| VWA3B | 1 | 12 |
| FSTL5 | 1 | 13 |
| NKAIN4 | 1 | 15 |
| RAC2 | 1 | 16 |
| CRP | 1 | 20 |
| VCX2 | 1 | 83 |
| KANSL1 | 1 | 8 |
| S100A16 | 1 | 9 |
| NLGN4Y | 1 | 11 |
| OR10G8 | 1 | 11 |
| GSTK1 | 1 | 12 |
| LTB4R2 | 1 | 12 |
| LIPH | 1 | 19 |
| CLSTN3 | 1 | 24 |
| CYP2A6 | 1 | 27 |
| DUX3 | 1 | 9 |
| B3GNT2 | 1 | 12 |
| OR4F29 | 1 | 12 |
| TBC1D5 | 1 | 12 |
| APOC3 | 1 | 13 |
| MAGEA1 | 1 | 23 |
| SMAGP | 1 | 31 |
| WNT10B | 1 | 39 |
| CD200R1 | 1 | 7 |
| KCNJ3 | 1 | 7 |
| SH3PXD2A | 1 | 9 |
| P2RY6 | 1 | 12 |
| METTL9 | 1 | 17 |
| THYN1 | 1 | 17 |
| PRIMA1 | 1 | 24 |
| AGTPBP1 | 1 | 5 |
| NEUROG3 | 1 | 9 |
| RNF213 | 1 | 9 |
| RTN4R | 1 | 11 |
| MKNK2 | 1 | 16 |
| NCAPD3 | 1 | 19 |
| GNB2 | 1 | 21 |
| DPF2 | 1 | 23 |
| TICAM2 | 1 | 23 |
| CDKN2D | 1 | 25 |
| DPF1 | 1 | 25 |
| C17orf101 | 1 | 7 |
| HVCN1 | 1 | 7 |
| C22orf40 | 1 | 9 |
| HPGDS | 1 | 9 |
| AK4 | 1 | 11 |
| KIAA0101 | 1 | 11 |
| RDH11 | 1 | 19 |
| GLT8D2 | 1 | 31 |
| C2orf68 | 1 | 40 |
| ALOX15B | 1 | 3 |
| KLHDC2 | 1 | 7 |
| DEFB110 | 1 | 8 |
| CALR3 | 1 | 9 |
| OR52I1 | 1 | 9 |
| ZADH2 | 1 | 9 |
| PWP1 | 1 | 15 |
| DYRK2 | 1 | 16 |
| GDF2 | 1 | 16 |
| GTDC2 | 1 | 19 |
| OR4C6 | 1 | 19 |
| PRSS2 | 1 | 19 |
| BAGE3 | 1 | 7 |
| IGHMBP2 | 1 | 9 |
| KCNE1 | 1 | 9 |
| OS9 | 1 | 13 |
| MAFK | 1 | 17 |
| HOXD1 | 1 | 23 |
| PSRC1 | 1 | 27 |
| NT5C3 | 1 | 33 |
| GCK | 1 | 4 |
| VTCN1 | 1 | 7 |
| BTBD9 | 1 | 9 |
| GTF3C2 | 1 | 9 |
| TBC1D10B | 1 | 9 |
| CTAGE6P | 1 | 11 |
| DRAM2 | 1 | 12 |
| KRT23 | 1 | 31 |
| NDUFV3 | 1 | 44 |
| CSNK1G2 | 1 | 3 |
| COMMD2 | 1 | 7 |
| KIAA1217 | 1 | 7 |
| RLN2 | 1 | 7 |
| C18orf54 | 1 | 9 |
| SSTR4 | 1 | 9 |
| ANAPC13 | 1 | 13 |
| SIRPD | 1 | 15 |
| U2AF1L4 | 1 | 19 |
| AIF1 | 1 | 27 |
| FGFBP1 | 1 | 29 |
| NMS | 1 | 4 |
| PCOLCE2 | 1 | 4 |
| FKBP2 | 1 | 5 |
| GFOD2 | 1 | 5 |
| EPM2AIP1 | 1 | 7 |
| C8orf4 | 1 | 8 |
| ELSPBP1 | 1 | 8 |
| GPR156 | 1 | 8 |
| RIBC1 | 1 | 8 |
| BTN2A1 | 1 | 11 |
| DUS2L | 1 | 11 |
| GHRHR | 1 | 11 |
| LIG4 | 1 | 12 |
| C4BPB | 1 | 13 |
| LYPD2 | 1 | 13 |
| MATN1 | 1 | 13 |
| SERPINF1 | 1 | 13 |
| ANKLE2 | 1 | 15 |
| INS | 1 | 17 |
| NCLN | 1 | 17 |
| CH25H | 1 | 19 |
| DIS3 | 1 | 19 |
| SH3GL3 | 1 | 35 |
| KRTAP23-1 | 1 | 47 |
| NXT1 | 1 | 1 |
| PITPNB | 1 | 1 |
| EVI5 | 1 | 3 |
| HADH | 1 | 3 |
| RELT | 1 | 4 |
| STAM | 1 | 4 |
| DNAJB5 | 1 | 5 |
| MALSU1 | 1 | 5 |
| ODF2L | 1 | 7 |
| TIGD7 | 1 | 7 |
| MEI1 | 1 | 8 |
| CHCHD10 | 1 | 9 |
| TNFRSF11B | 1 | 11 |
| MLLT10 | 1 | 13 |
| FAM13A | 1 | 15 |
| NSMCE4A | 1 | 20 |
| TESPA1 | 1 | 20 |
| C1orf43 | 1 | 23 |
| ESCO2 | 1 | 25 |
| SMPD1 |  | 20 |
| LHPP |  | 9 |
| IL13 |  | 35 |
| CBFB |  | 19 |
| CRH |  | 8 |
| PARPBP |  | 8 |
| FAM176B |  | 20 |
| ZNF680 |  | 11 |
| MRPS27 |  | 37 |
| SH2D1B |  | 21 |
| GTF2H5 |  | 15 |
| CPNE3 |  | 19 |
| ADCK4 |  | 5 |
| BOLA1 |  | 21 |
| NRN1 |  | 8 |
| COX7B |  | 25 |
| PLA2G1B |  | 12 |
| MUM1 |  | 27 |
| CXorf58 |  | 17 |
| GCAT |  | 9 |
| HAVCR2 |  | 27 |
| TSFM |  | 7 |
| EMC2 |  | 15 |
| H1FNT |  | 35 |
| RGS13 |  | 32 |
| SPPL2B |  | 8 |
| CCNC |  | 36 |
| ZNF611 |  | 13 |
| RGS21 |  | 9 |
| NDUFA1 |  | 23 |
| C1orf31 |  | 9 |
| CHMP3 |  | 12 |
| FAM109A |  | 27 |
| RAB2B |  | 19 |
| TRIM31 |  | 35 |
| SYT3 |  | 25 |
| TNFRSF11A |  | 7 |
| HYAL3 |  | 23 |
| SH3BGRL2 |  | 13 |
| PLA2G12B |  | 27 |
| COPS2 |  | 9 |
| MOB3C |  | 12 |
| FAM175A |  | 29 |
| HES7 |  | 7 |
| UBL4B |  | 21 |
| RINL |  | 29 |
| GCDH |  | 11 |
| MED29 |  | 15 |
| ARHGEF7 |  | 29 |
| IL18RAP |  | 8 |
| GPR68 |  | 21 |
| DEF6 |  | 11 |
| NDUFA12 |  | 32 |
| RGS6 |  | 7 |
| PRSS22 |  | 16 |
| LCN9 |  | 21 |
| PAQR3 |  | 23 |
| EPS15L1 |  | 15 |
| DEFA6 |  | 27 |
| FBXL22 |  | 8 |
| STAU2 |  | 20 |
| YIPF7 |  | 23 |
| GLOD4 |  | 7 |
| MED10 |  | 24 |
| CCNG2 |  | 28 |
| TLE3 |  | 16 |
| RAB34 |  | 43 |
| PRSS42 |  | 7 |
| FAM126B |  | 12 |
| TNFRSF10D |  | 20 |
| ADAMTS4 |  | 37 |
| PCDHA4 |  | 9 |
| RPP21 |  | 12 |
| MORN2 |  | 37 |
| CTXN1 |  | 5 |
| COPS7B |  | 9 |
| CDKN3 |  | 19 |
| XG |  | 19 |
| TMEM95 |  | 20 |
| CEP19 |  | 80 |
| OR5AP2 |  | 28 |
| RALB |  | 20 |
| GSTA4 |  | 9 |
| MAP1LC3B2 |  | 11 |
| NUAK2 |  | 17 |
| CCL8 |  | 23 |
| GHRH |  | 16 |
| UBL4A |  | 16 |
| PCDHGC3 |  | 21 |
| CTSK |  | 15 |
| HES2 |  | 13 |
| EEPD1 |  | 16 |
| CDNF |  | 29 |
| RNF24 |  | 7 |
| ANKRD65 |  | 25 |
| SNPH |  | 12 |
| CHCHD5 |  | 17 |
| TK2 |  | 8 |
| TRAM2 |  | 11 |
| TROVE2 |  | 5 |
| UBE2W |  | 24 |
| CMC2 |  | 27 |
| NSRP1 |  | 13 |
| PRY |  | 24 |
| IL29 |  | 7 |
| VMO1 |  | 17 |
| CGB1 |  | 24 |
| C20orf85 |  | 33 |
| DEFB1 |  | 16 |
| GPN3 |  | 19 |
| GPR171 |  | 41 |
| PSPN |  | 12 |
| RHOA |  | 20 |
| TP53I11 |  | 28 |
| INTS12 |  | 11 |
| FXYD1 |  | 37 |
| HIST1H3G |  | 5 |
| DDX11 |  | 8 |
| SRPK2 |  | 49 |
| COX7B2 |  | 5 |
| TCEB3B |  | 7 |
| ARRB2 |  | 8 |
| MLPH |  | 17 |
| CTRB2 |  | 21 |
| STX17 |  | 29 |
| RAB7B |  | 33 |
| POLR2J2 |  | 7 |
| MAP3K13 |  | 9 |
| DDX1 |  | 12 |
| ING3 |  | 12 |
| PCDHAC1 |  | 12 |
| MAP3K3 |  | 16 |
| TCF7 |  | 28 |
| WDR3 |  | 43 |
| PYCRL |  | 5 |
| CXCL2 |  | 8 |
| RCCD1 |  | 17 |
| FGL2 |  | 25 |
| ACAD10 |  | 29 |
| BIRC5 |  | 8 |
| RASGRF1 |  | 12 |
| C19orf73 |  | 19 |
| DHX37 |  | 28 |
| IGIP |  | 12 |
| ZC3H7A |  | 12 |
| OTUD3 |  | 23 |
| SNX11 |  | 24 |
| C19orf44 |  | 25 |
| C2orf83 |  | 11 |
| C6orf221 |  | 37 |
| ICAM2 |  | 5 |
| RNF212 |  | 7 |
| ASH2L |  | 9 |
| MED11 |  | 15 |
| MFSD5 |  | 19 |
| SAP18 |  | 33 |
| NFATC2IP |  | 11 |
| MBLAC1 |  | 16 |
| PCDHB11 |  | 16 |
| FAM105B |  | 37 |
| DMBX1 |  | 84 |
| ACTL6B |  | 5 |
| MBTD1 |  | 5 |
| ZNF492 |  | 5 |
| SNX21 |  | 7 |
| TBC1D4 |  | 7 |
| PTPRH |  | 12 |
| UBXN4 |  | 13 |
| MYCT1 |  | 16 |
| RCOR3 |  | 21 |
| RAB23 |  | 25 |
| ROGDI |  | 33 |
| KIR3DL1 |  | 11 |
| DFNA5 |  | 16 |
| KLK13 |  | 19 |
| COX4I1 |  | 23 |
| GPR146 |  | 24 |
| TMEM131 |  | 31 |
| CENPO |  | 5 |
| SEC22C |  | 20 |
| PSMB7 |  | 8 |
| MSRA |  | 9 |
| PEX3 |  | 9 |
| NPY |  | 19 |
| GGTLC2 |  | 24 |
| COX16 |  | 8 |
| FIGLA |  | 17 |
| GLRA2 |  | 28 |
| C16orf53 |  | 8 |
| KRT2 |  | 8 |
| FCF1 |  | 12 |
| OR5H1 |  | 13 |
| DUSP22 |  | 16 |
| C11orf71 |  | 28 |
| NDUFB2 |  | 5 |
| REG1A |  | 5 |
| PTPN18 |  | 13 |
| NPHP1 |  | 20 |
| NUS1 |  | 27 |
| GDF10 |  | 28 |
| SERPINB11 |  | 5 |
| NEFM |  | 7 |
| POLR1C |  | 7 |
| C19orf10 |  | 8 |
| DNAJC28 |  | 9 |
| DPM1 |  | 12 |
| GNB5 |  | 19 |
| ZNF672 |  | 24 |
| CALHM1 |  | 36 |
| AGR3 |  | 39 |
| PCDHB16 |  | 40 |
| MRPL45 |  | 60 |
| KIAA0226L |  | 7 |
| LRAT |  | 8 |
| BET1L |  | 16 |
| ZFP36L2 |  | 16 |
| SNX4 |  | 19 |
| FKBP7 |  | 7 |
| C18orf62 |  | 8 |
| LCA5L |  | 8 |
| DNAJC12 |  | 13 |
| RAB5C |  | 16 |
| FAM213B |  | 21 |
| LIMS2 |  | 21 |
| POMC |  | 37 |
| PLAU |  | 7 |
| LAMTOR2 |  | 8 |
| CCL19 |  | 12 |
| FAM127C |  | 12 |
| MRPS15 |  | 24 |
| MTFR1 |  | 24 |
| CAPN13 |  | 29 |
| OR1J2 |  | 31 |
| APLN |  | 32 |
| CTAG2 |  | 4 |
| HNRNPCL1 |  | 5 |
| GK2 |  | 8 |
| RETNLB |  | 8 |
| MED7 |  | 11 |
| MCF2L |  | 12 |
| NDUFS6 |  | 12 |
| ZNF707 |  | 12 |
| WNT8A |  | 13 |
| ACP5 |  | 16 |
| SATB1 |  | 16 |
| LDOC1L |  | 20 |
| CTAGE5 |  | 32 |
| TUBB8 |  | 4 |
| GLB1L3 |  | 5 |
| CDKN1B |  | 7 |
| LRG1 |  | 8 |
| RWDD4 |  | 8 |
| TAGLN |  | 12 |
| MTFP1 |  | 13 |
| RASL10A |  | 15 |
| MOB2 |  | 16 |
| PRR23B |  | 19 |
| RASA3 |  | 25 |
| C12orf61 |  | 31 |
| DEFB112 |  | 32 |
| RASL11A |  | 37 |
| ARFRP1 |  | 4 |
| ATP5L |  | 7 |
| DCAF4 |  | 9 |
| SPRR2G |  | 9 |
| GNAI3 |  | 13 |
| EXD3 |  | 15 |
| C20orf166 |  | 19 |
| RTKN |  | 20 |
| DCK |  | 8 |
| PTEN |  | 8 |
| ADORA3 |  | 11 |
| FAM206A |  | 11 |
| CCDC11 |  | 12 |
| CDK2AP2 |  | 13 |
| HLA-A |  | 20 |
| TUBGCP3 |  | 24 |
| NANOS1 |  | 35 |
| CLEC10A |  | 7 |
| CHCHD8 |  | 8 |
| GNA13 |  | 8 |
| OCIAD2 |  | 8 |
| PNPLA2 |  | 8 |
| ZNF616 |  | 9 |
| FAM98A |  | 15 |
| NAT8L |  | 17 |
| APOC2 |  | 21 |
| TLR2 |  | 23 |
| KDELC1 |  | 28 |
| RIC8B |  | 28 |
| PAM16 |  | 31 |
| HSD17B6 |  | 37 |
| HIGD1C |  | 5 |
| TPGS1 |  | 5 |
| HNRPDL |  | 8 |
| IL17RC |  | 8 |
| FRG1 |  | 11 |
| MED21 |  | 11 |
| RASSF8 |  | 11 |
| NGRN |  | 12 |
| GPATCH3 |  | 13 |
| APTX |  | 15 |
| KIAA1683 |  | 16 |
| NDRG1 |  | 16 |
| WISP3 |  | 17 |
| TAS2R31 |  | 20 |
| SDR9C7 |  | 27 |
| KAAG1 |  | 5 |
| CAV1 |  | 7 |
| EMCN |  | 7 |
| HIST3H2BB |  | 7 |
| SLPI |  | 7 |
| TIMM13 |  | 7 |
| ARG1 |  | 8 |
| CCDC48 |  | 8 |
| MRPS6 |  | 11 |
| POLE4 |  | 11 |
| BEST1 |  | 12 |
| DIRAS1 |  | 12 |
| HIC2 |  | 15 |
| PARP15 |  | 23 |
| CHMP4C |  | 24 |
| ELAC2 |  | 25 |
| CXCL13 |  | 27 |
| C10orf81 |  | 29 |
| GLTP |  | 29 |
| NDUFS5 |  | 31 |
| DUSP21 |  | 7 |
| OBFC2B |  | 7 |
| CHAC2 |  | 11 |
| RDH14 |  | 16 |
| FKBP6 |  | 19 |
| TTK |  | 19 |
| RBM23 |  | 20 |
| VPS28 |  | 21 |
| GRAPL |  | 41 |
| AKAP17A |  | 4 |
| LILRB3 |  | 5 |
| NMNAT3 |  | 7 |
| SF3B5 |  | 7 |
| GLMN |  | 9 |
| FAM13C |  | 12 |
| RBMS1 |  | 12 |
| TNFSF10 |  | 12 |
| ZFPL1 |  | 12 |
| C16orf78 |  | 13 |
| GTSF1 |  | 13 |
| UBXN6 |  | 13 |
| FAM185A |  | 16 |
| MRPS24 |  | 16 |
| NT5C |  | 19 |
| SH3GL2 |  | 19 |
| IL18 |  | 25 |
| S100A14 |  | 25 |
| MZT1 |  | 29 |
| NPC2 |  | 7 |
| AMMECR1 |  | 8 |
| ANXA2 |  | 8 |
| CST9 |  | 9 |
| SLC2A6 |  | 11 |
| SLC22A7 |  | 12 |
| STK4 |  | 15 |
| C2orf76 |  | 17 |
| DEFB134 |  | 17 |
| ZKSCAN4 |  | 17 |
| GNG10 |  | 20 |
| POLD3 |  | 20 |
| FMNL1 |  | 21 |
| TCEAL7 |  | 35 |
| KLK6 |  | 36 |
| WDR20 |  | 5 |
| WDR31 |  | 5 |
| GRIN2C |  | 7 |
| SORBS1 |  | 7 |
| FBXL15 |  | 8 |
| LINGO1 |  | 8 |
| STYK1 |  | 8 |
| PARL |  | 9 |
| TAC4 |  | 9 |
| TUBG1 |  | 9 |
| LIMS3 |  | 11 |
| VPS39 |  | 13 |
| MRGPRX3 |  | 15 |
| HBEGF |  | 16 |
| MAPT |  | 16 |
| SKP1 |  | 16 |
| PYHIN1 |  | 23 |
| FDX1L |  | 24 |
| GBP3 |  | 24 |
| LRRC45 |  | 24 |
| SPRR2E |  | 27 |
| ARID3C |  | 52 |
| C19orf80 |  | 4 |
| PFDN1 |  | 5 |
| RPF1 |  | 7 |
| SIRT6 |  | 7 |
| PIPOX |  | 8 |
| ZSWIM7 |  | 9 |
| RNASE2 |  | 11 |
| CHODL |  | 12 |
| LYZ |  | 12 |
| RPS6 |  | 12 |
| TAF5L |  | 12 |
| STX11 |  | 13 |
| APOA1 |  | 16 |
| CUL3 |  | 16 |
| IP6K2 |  | 17 |
| SRP68 |  | 17 |
| SUGP2 |  | 19 |
| ARAP3 |  | 20 |
| BLOC1S1 |  | 20 |
| NOL7 |  | 20 |
| TMX1 |  | 23 |
| NDUFAF1 |  | 25 |
| C22orf42 |  | 27 |
| TNNT2 |  | 28 |
| AGMAT |  | 3 |
| C19orf40 |  | 7 |
| ARHGEF18 |  | 8 |
| FAM86C1 |  | 8 |
| MAX |  | 9 |
| RGS1 |  | 9 |
| ZNF530 |  | 9 |
| NXT2 |  | 12 |
| RPL5 |  | 12 |
| ZNF333 |  | 12 |
| GCNT3 |  | 15 |
| PSMD8 |  | 16 |
| DEFB119 |  | 17 |
| GPX4 |  | 17 |
| RABL2B |  | 17 |
| C6orf141 |  | 20 |
| UNC119B |  | 23 |
| MCM5 |  | 28 |
| PPARD |  | 37 |
| HLA-F |  | 48 |
| NDST4 |  | 5 |
| PHOSPHO1 |  | 5 |
| UEVLD |  | 5 |
| CSH2 |  | 7 |
| CXCL1 |  | 8 |
| HMGCS2 |  | 8 |
| IL5RA |  | 8 |
| NCAPG |  | 8 |
| CYB561D2 |  | 9 |
| C12orf50 |  | 11 |
| C2orf88 |  | 12 |
| SRA1 |  | 12 |
| NCOA7 |  | 15 |
| C1orf115 |  | 16 |
| C8B |  | 16 |
| PIAS4 |  | 16 |
| APOPT1 |  | 17 |
| MXD4 |  | 19 |
| WDR86 |  | 19 |
| ECH1 |  | 20 |
| ADAM19 |  | 23 |
| PQLC1 |  | 23 |
| INTS5 |  | 27 |
| TMPRSS5 |  | 27 |
| C17orf102 |  | 44 |
| FMO5 |  | 4 |
| SNX29 |  | 5 |
| SPTB |  | 5 |
| LASP1 |  | 7 |
| LCMT1 |  | 7 |
| PNMA5 |  | 7 |
| TRAPPC2 |  | 7 |
| TREX2 |  | 7 |
| FZR1 |  | 8 |
| GCHFR |  | 8 |
| SLC19A1 |  | 8 |
| ZMYND19 |  | 8 |
| MRPS18A |  | 9 |
| SLC36A3 |  | 9 |
| NUDT18 |  | 12 |
| RNF114 |  | 12 |
| SPEF1 |  | 12 |
| ECSIT |  | 15 |
| MLNR |  | 15 |
| NUBP1 |  | 15 |
| SNRPD2 |  | 15 |
| FKBPL |  | 16 |
| HIGD2A |  | 17 |
| ADAT2 |  | 20 |
| C16orf93 |  | 21 |
| CNN1 |  | 21 |
| HCLS1 |  | 21 |
| MUC1 |  | 23 |
| PARK2 |  | 24 |
| FUBP3 |  | 25 |
| LRRC28 |  | 25 |
| ZNF501 |  | 31 |
| LMO3 |  | 61 |
| CYTIP |  | 3 |
| FAM150B |  | 5 |
| HSFX2 |  | 5 |
| MOXD1 |  | 7 |
| RPP30 |  | 7 |
| VPREB1 |  | 7 |
| KIF9 |  | 8 |
| ANGPT4 |  | 9 |
| OXCT2 |  | 9 |
| SLC22A9 |  | 9 |
| C4orf36 |  | 11 |
| MITD1 |  | 11 |
| TNIP3 |  | 11 |
| IDO2 |  | 12 |
| IFT81 |  | 12 |
| RHBDD1 |  | 19 |
| KLHL36 |  | 21 |
| GHRL |  | 23 |
| FAM124B |  | 24 |
| TTC14 |  | 28 |
| PGGT1B |  | 33 |
| ZSCAN16 |  | 33 |
| PCGF5 |  | 37 |
| DUSP10 |  | 56 |
| FNTA |  | 69 |
| IGDCC4 |  | 3 |
| INTS4 |  | 4 |
| PCDHA9 |  | 4 |
| TRIOBP |  | 4 |
| PLSCR1 |  | 5 |
| UNC45A |  | 5 |
| CRIP3 |  | 7 |
| SCT |  | 7 |
| CNTN2 |  | 8 |
| FAM175B |  | 8 |
| HN1L |  | 8 |
| TMEM62 |  | 9 |
| C8G |  | 11 |
| SEC31B |  | 11 |
| PTP4A2 |  | 12 |
| DACT2 |  | 13 |
| IL2RG |  | 13 |
| SDF2L1 |  | 13 |
| UST |  | 13 |
| CHST12 |  | 15 |
| LGALS9B |  | 16 |
| RND3 |  | 17 |
| ZNF323 |  | 17 |
| BCHE |  | 19 |
| CGB8 |  | 21 |
| PPP1CC |  | 23 |
| SDHA |  | 25 |
| BRP44 |  | 27 |
| CHD9 |  | 27 |
| MXD1 |  | 28 |
| SNX17 |  | 4 |
| CDCP2 |  | 5 |
| LIN7C |  | 5 |
| SLC22A6 |  | 5 |
| C3orf45 |  | 7 |
| PTGES |  | 7 |
| KCNK1 |  | 8 |
| TRAF3IP3 |  | 8 |
| PTPN11 |  | 9 |
| FAM150A |  | 13 |
| G0S2 |  | 15 |
| MRPS34 |  | 15 |
| CDX2 |  | 16 |
| ELOVL3 |  | 17 |
| DCN |  | 19 |
| MAB21L1 |  | 19 |
| CCDC28B |  | 20 |
| POU5F2 |  | 20 |
| USMG5 |  | 20 |
| UVSSA |  | 25 |
| TGM3 |  | 28 |
| BEND6 |  | 29 |
| RETSAT |  | 29 |
| ATP6V1D |  | 31 |
| RPS21 |  | 39 |
| PMS1 |  | 40 |
| EMILIN3 |  | 43 |
| NETO2 |  | 44 |
| AREG |  | 3 |
| MRPL27 |  | 3 |
| CDK4 |  | 4 |
| DNAJB1 |  | 4 |
| ACOT12 |  | 5 |
| STAR |  | 5 |
| TOR1B |  | 5 |
| ZNF572 |  | 5 |
| C3orf35 |  | 7 |
| DUSP18 |  | 7 |
| FUZ |  | 7 |
| SNX15 |  | 7 |
| MCTS1 |  | 8 |
| PPP6R2 |  | 8 |
| TOX4 |  | 8 |
| ADCYAP1 |  | 9 |
| AWAT1 |  | 9 |
| FGD3 |  | 9 |
| GANAB |  | 9 |
| PPIL1 |  | 9 |
| DNAJC2 |  | 11 |
| DYNC1LI1 |  | 11 |
| AHSP |  | 12 |
| IFIH1 |  | 12 |
| DGUOK |  | 13 |
| RPS26 |  | 13 |
| TMCO6 |  | 13 |
| CNDP1 |  | 15 |
| KIF25 |  | 15 |
| TMTC2 |  | 15 |
| ENDOV |  | 16 |
| AMELY |  | 17 |
| MTHFD2L |  | 17 |
| SSX4 |  | 19 |
| CCDC121 |  | 20 |
| RUSC1-AS1 |  | 21 |
| COX6B1 |  | 23 |
| TTC29 |  | 24 |
| KCNMA1 |  | 25 |
| AMMECR1L |  | 27 |
| PTMS |  | 27 |
| TMPRSS11E |  | 27 |
| PAPLN |  | 28 |
| GEMIN7 |  | 32 |
| ADIG |  | 35 |
| COA5 |  | 35 |
| RSPO3 |  | 39 |
| C21orf62 |  | 3 |
| TMEM64 |  | 3 |
| CA5B |  | 5 |
| EPDR1 |  | 5 |
| FAM116A |  | 5 |
| RAB36 |  | 5 |
| UBE2D1 |  | 5 |
| ARHGAP11A |  | 7 |
| FGFBP2 |  | 7 |
| ARL5A |  | 8 |
| C11orf54 |  | 8 |
| MAPK13 |  | 11 |
| TFPI2 |  | 11 |
| WAPAL |  | 11 |
| SLURP1 |  | 12 |
| PDE4D |  | 13 |
| ZCCHC12 |  | 13 |
| UBE2S |  | 16 |
| LRRIQ3 |  | 17 |
| ZMYM5 |  | 17 |
| NYAP2 |  | 20 |
| RARA |  | 21 |
| C19orf47 |  | 23 |
| GBA |  | 23 |
| BCL6B |  | 27 |
| WBP5 |  | 28 |
| C19orf55 |  | 29 |
| CCL26 |  | 32 |
| TMEM246 |  | 32 |
| S1PR1 |  | 40 |
| EXT1 |  | 4 |
| GCFC1 |  | 4 |
| RFX3 |  | 4 |
| TLR7 |  | 4 |
| C17orf90 |  | 5 |
| FAXC |  | 5 |
| GPR113 |  | 5 |
| KIF2B |  | 5 |
| NLRP5 |  | 5 |
| CMKLR1 |  | 7 |
| GALNS |  | 7 |
| HPSE2 |  | 7 |
| PKP2 |  | 7 |
| RFXAP |  | 7 |
| ZAR1L |  | 7 |
| ATP6V0D2 |  | 8 |
| ICAM4 |  | 8 |
| PACRG |  | 8 |
| C4orf29 |  | 9 |
| FAM35A |  | 9 |
| SLC12A9 |  | 9 |
| TADA2B |  | 9 |
| ZNF416 |  | 9 |
| RPL14 |  | 11 |
| SLC35E2B |  | 11 |
| CHD1L |  | 12 |
| CTNNBIP1 |  | 12 |
| PCLO |  | 12 |
| SLMO1 |  | 12 |
| TCF23 |  | 12 |
| TOR1A |  | 12 |
| KCTD3 |  | 13 |
| SPRR2F |  | 13 |
| ZNF280C |  | 15 |
| BTG3 |  | 16 |
| EIF3G |  | 16 |
| LRRTM2 |  | 16 |
| TSGA13 |  | 16 |
| FAM89B |  | 17 |
| FLOT2 |  | 17 |
| SCEL |  | 17 |
| TMEM89 |  | 17 |
| FAM27E3 |  | 19 |
| INTU |  | 21 |
| FCGR2B |  | 23 |
| GOLGA7B |  | 23 |
| HRASLS |  | 27 |
| NTF3 |  | 29 |
| SLN |  | 32 |
| KIAA0247 |  | 33 |
| C11orf84 |  | 36 |
| CHRNG |  | 36 |
| HEXIM1 |  | 61 |
| HMX1 |  | 79 |
| FSTL4 |  | 3 |
| GABARAP |  | 3 |
| GATAD2A |  | 4 |
| PDPN |  | 4 |
| PEMT |  | 4 |
| TMTC1 |  | 4 |
| ATPAF1 |  | 5 |
| DMRTC2 |  | 5 |
| IGBP1 |  | 5 |
| MLC1 |  | 5 |
| NR0B2 |  | 5 |
| PDK1 |  | 5 |
| GINS2 |  | 7 |
| KRTAP5-4 |  | 7 |
| PDC |  | 7 |
| PGLYRP1 |  | 7 |
| PRR14L |  | 7 |
| RAB1B |  | 7 |
| SPATA19 |  | 7 |
| SYT17 |  | 7 |
| CPT1A |  | 8 |
| GUSB |  | 8 |
| KCNAB2 |  | 8 |
| LHFPL2 |  | 8 |
| OR6B2 |  | 8 |
| PFN4 |  | 8 |
| RPS28 |  | 8 |
| TYW1B |  | 8 |
| CYP11B1 |  | 9 |
| RAD54L2 |  | 9 |
| COX6A1 |  | 11 |
| CCDC151 |  | 12 |
| GALNT13 |  | 12 |
| IL31RA |  | 12 |
| REG1B |  | 12 |
| TAS2R41 |  | 12 |
| TCEA2 |  | 12 |
| MBP |  | 13 |
| RBM48 |  | 13 |
| DEC1 |  | 15 |
| PRSS33 |  | 15 |
| DRD3 |  | 16 |
| HIST2H3D |  | 16 |
| HS2ST1 |  | 16 |
| MED17 |  | 17 |
| PATL1 |  | 17 |
| ELMO1 |  | 19 |
| TXNDC9 |  | 19 |
| ARHGAP19 |  | 20 |
| TMEM126B |  | 20 |
| C6orf225 |  | 21 |
| C9 |  | 21 |
| DYNC1LI2 |  | 21 |
| LRRC36 |  | 25 |
| CFHR3 |  | 29 |
| EN1 |  | 29 |
| RNASE9 |  | 29 |
| SCMH1 |  | 29 |
| SLA2 |  | 29 |
| TEX264 |  | 36 |
| ZNF561 |  | 37 |
| PDE6H |  | 39 |
| RPL27A |  | 39 |
| CLPSL2 |  | 1 |
| CD300C |  | 3 |
| PLA2G7 |  | 3 |
| S100A7 |  | 3 |
| ABCE1 |  | 4 |
| ABHD14A |  | 4 |
| AGA |  | 4 |
| ARHGEF6 |  | 4 |
| CCL3L3 |  | 4 |
| EDAR |  | 4 |
| NDUFAF4 |  | 4 |
| SLC35G5 |  | 4 |
| VPS26B |  | 4 |
| YRDC |  | 4 |
| CAMK1 |  | 5 |
| MDP1 |  | 5 |
| OTUB1 |  | 5 |
| C1orf204 |  | 7 |
| DGKG |  | 7 |
| IGFALS |  | 7 |
| MMS19 |  | 7 |
| PDGFRL |  | 7 |
| SLC15A1 |  | 7 |
| STAG3 |  | 7 |
| UTP11L |  | 7 |
| CLIC5 |  | 8 |
| SLC17A8 |  | 8 |
| TEX13A |  | 8 |
| TIMP4 |  | 8 |
| FAM219B |  | 9 |
| SLC22A8 |  | 9 |
| FLRT1 |  | 11 |
| AZU1 |  | 12 |
| PFN2 |  | 12 |
| DMC1 |  | 13 |
| C1orf61 |  | 15 |
| FAM200A |  | 15 |
| FBXL4 |  | 15 |
| CTSG |  | 16 |
| CDCA8 |  | 17 |
| ZNF101 |  | 17 |
| GDF5 |  | 19 |
| DAP3 |  | 20 |
| PTPDC1 |  | 20 |
| SULT1C4 |  | 20 |
| MRPL2 |  | 21 |
| TREM1 |  | 21 |
| NAA10 |  | 23 |
| HEY2 |  | 27 |
| PNPO |  | 27 |
| SPEM1 |  | 27 |
| ECT2 |  | 28 |
| GRIN1 |  | 28 |
| DACH2 |  | 29 |
| IQCF1 |  | 31 |
| ZNF354C |  | 31 |
| CD79B |  | 35 |
| TMEM234 |  | 40 |
| ZNF276 |  | 40 |
| TRNT1 |  | 48 |
| MMAB |  | 3 |
| PRKAB2 |  | 3 |
| CLDN15 |  | 4 |
| SPATA2L |  | 4 |
| SPDYE4 |  | 4 |
| C5orf49 |  | 5 |
| FAM58A |  | 5 |
| HDAC6 |  | 5 |
| NIPA1 |  | 5 |
| NIPAL3 |  | 5 |
| ARHGEF40 |  | 7 |
| C1orf228 |  | 7 |
| EXOG |  | 7 |
| JUNB |  | 7 |
| LRFN2 |  | 7 |
| LY96 |  | 7 |
| PRKCB |  | 7 |
| SPANXN4 |  | 7 |
| ST7L |  | 7 |
| TAS2R50 |  | 7 |
| THNSL1 |  | 7 |
| CCDC37 |  | 8 |
| CD33 |  | 8 |
| COMT |  | 8 |
| DYX1C1 |  | 8 |
| GUCA1B |  | 8 |
| NHLRC3 |  | 8 |
| RASSF1 |  | 8 |
| MICU1 |  | 9 |
| SASS6 |  | 9 |
| C7orf69 |  | 11 |
| DDHD2 |  | 11 |
| LIAS |  | 11 |
| MRPL52 |  | 11 |
| CCDC24 |  | 12 |
| OLFM3 |  | 12 |
| RPS4X |  | 12 |
| DEPDC1 |  | 13 |
| IL16 |  | 13 |
| TMEM223 |  | 13 |
| BIN3 |  | 15 |
| MORN4 |  | 15 |
| POLR3E |  | 15 |
| ZNF419 |  | 15 |
| GPR119 |  | 16 |
| PODN |  | 16 |
| RAB28 |  | 16 |
| NCALD |  | 17 |
| 38961 |  | 19 |
| C4orf26 |  | 19 |
| GH2 |  | 19 |
| ODF3L2 |  | 19 |
| POLM |  | 19 |
| SLC23A2 |  | 19 |
| SLC38A2 |  | 19 |
| ARHGEF3 |  | 21 |
| TMPRSS11B |  | 21 |
| CENPQ |  | 23 |
| CYP2C9 |  | 23 |
| DKK2 |  | 23 |
| APCS |  | 27 |
| ATF2 |  | 28 |
| P2RY14 |  | 28 |
| C5orf58 |  | 32 |
| FOSL2 |  | 41 |
| HIST1H2BJ |  | 41 |
| LAG3 |  | 1 |
| OR2B6 |  | 1 |
| RORB |  | 3 |
| SRPK3 |  | 3 |
| APOBEC3H |  | 4 |
| DCAF4L2 |  | 4 |
| SLBP |  | 4 |
| TMEM178 |  | 4 |
| ART1 |  | 5 |
| CEP63 |  | 5 |
| PDSS1 |  | 5 |
| PHACTR2 |  | 5 |
| POPDC2 |  | 5 |
| UCMA |  | 5 |
| UPRT |  | 5 |
| ACADS |  | 7 |
| ANTXR2 |  | 7 |
| ATOX1 |  | 7 |
| MSMB |  | 7 |
| MTHFD2 |  | 7 |
| RHOF |  | 7 |
| VAC14 |  | 7 |
| 37135 |  | 8 |
| CKMT1A |  | 8 |
| CLDN3 |  | 8 |
| DEFB104B |  | 8 |
| EIF4H |  | 8 |
| KCNH2 |  | 8 |
| PLA2G2F |  | 8 |
| PLA2G4D |  | 8 |
| PTPRCAP |  | 8 |
| RAMP3 |  | 8 |
| RBP5 |  | 8 |
| SF3A2 |  | 8 |
| COMMD3 |  | 9 |
| ISG20 |  | 9 |
| TMEM59 |  | 9 |
| UGT2A3 |  | 9 |
| ZMAT2 |  | 9 |
| DFFA |  | 11 |
| DTD1 |  | 11 |
| SGCE |  | 11 |
| HMGN3 |  | 12 |
| MRPL13 |  | 12 |
| OR10S1 |  | 12 |
| RPL26L1 |  | 12 |
| LAMA4 |  | 13 |
| LHX8 |  | 13 |
| C2orf28 |  | 15 |
| MBD3L1 |  | 15 |
| BPI |  | 16 |
| C17orf56 |  | 16 |
| C3orf18 |  | 16 |
| COX5A |  | 16 |
| TCF25 |  | 16 |
| TMEM179B |  | 16 |
| ZNF599 |  | 16 |
| CST8 |  | 17 |
| HIGD1A |  | 17 |
| PTPN2 |  | 17 |
| GIP |  | 19 |
| SMPD3 |  | 19 |
| TMEM194A |  | 19 |
| WFDC13 |  | 19 |
| PTGER4 |  | 20 |
| STMN3 |  | 20 |
| CCDC127 |  | 21 |
| KIF19 |  | 21 |
| LATS1 |  | 21 |
| MTMR9 |  | 21 |
| ZFP82 |  | 21 |
| LYRM7 |  | 24 |
| MYL9 |  | 24 |
| IBSP |  | 29 |
| DNAJB12 |  | 32 |
| CHST9 |  | 33 |
| ANK1 |  | 37 |
| C1QTNF9B |  | 1 |
| PDXK |  | 1 |
| AKAP14 |  | 3 |
| HIST1H3J |  | 3 |
| MRAP2 |  | 3 |
| WARS2 |  | 3 |
| C3orf24 |  | 4 |
| CHMP2A |  | 4 |
| FGF10 |  | 4 |
| HTR1B |  | 4 |
| KDELC2 |  | 4 |
| TMEM74 |  | 4 |
| TOB1 |  | 4 |
| TTC32 |  | 4 |
| ZDHHC5 |  | 4 |
| ZNF136 |  | 4 |
| BYSL |  | 5 |
| CETP |  | 5 |
| CXorf40B |  | 5 |
| LACTB |  | 5 |
| NDUFB11 |  | 5 |
| PARD6B |  | 5 |
| SNAPC2 |  | 5 |
| ABCA9 |  | 7 |
| ALDH3B2 |  | 7 |
| DHRS2 |  | 7 |
| IL12RB2 |  | 7 |
| MOCS2 |  | 7 |
| PLEKHJ1 |  | 7 |
| UGT1A10 |  | 7 |
| VRTN |  | 7 |
| ZNF528 |  | 7 |
| ZNF808 |  | 7 |
| DEFB123 |  | 8 |
| KIAA2013 |  | 8 |
| NDUFV2 |  | 8 |
| SCGB1D4 |  | 8 |
| SEMA4C |  | 8 |
| SSTR5 |  | 8 |
| ENO3 |  | 9 |
| FGD4 |  | 9 |
| MSH5 |  | 9 |
| SGOL1 |  | 9 |
| TMEM26 |  | 9 |
| B3GAT1 |  | 11 |
| C1orf227 |  | 11 |
| C3orf14 |  | 11 |
| CCL23 |  | 11 |
| LRRC15 |  | 11 |
| C8orf31 |  | 12 |
| CCT8L2 |  | 12 |
| FAM48A |  | 12 |
| NFIB |  | 12 |
| PYY |  | 12 |
| SFRP2 |  | 12 |
| SMOC1 |  | 12 |
| SMPDL3A |  | 12 |
| FFAR2 |  | 13 |
| DCUN1D1 |  | 15 |
| DYNC1I1 |  | 15 |
| GPX5 |  | 15 |
| ITFG3 |  | 15 |
| MGP |  | 15 |
| BEX1 |  | 16 |
| FST |  | 16 |
| SPCS3 |  | 16 |
| CROT |  | 17 |
| FAM107B |  | 17 |
| LY6G5C |  | 17 |
| ACOT13 |  | 19 |
| ADAD2 |  | 19 |
| OPRL1 |  | 19 |
| MAD2L1BP |  | 20 |
| MNF1 |  | 20 |
| ZNF132 |  | 21 |
| DHX38 |  | 23 |
| ADCK3 |  | 24 |
| HOXB5 |  | 24 |
| RAB6C |  | 24 |
| TMEM196 |  | 25 |
| C6orf162 |  | 27 |
| ELOF1 |  | 27 |
| C8orf86 |  | 29 |
| C10orf32 |  | 32 |
| ZC4H2 |  | 49 |
| TRIM59 |  | 1 |
| APOF |  | 3 |
| DAAM2 |  | 3 |
| MUS81 |  | 3 |
| RNF39 |  | 3 |
| ST6GALNAC5 |  | 3 |
| GXYLT2 |  | 4 |
| LRP12 |  | 4 |
| NANS |  | 4 |
| PARN |  | 4 |
| THUMPD3 |  | 4 |
| TRIM16 |  | 4 |
| ZKSCAN2 |  | 4 |
| ANKK1 |  | 5 |
| BTD |  | 5 |
| CADM4 |  | 5 |
| CD80 |  | 5 |
| IGF2 |  | 5 |
| MFAP4 |  | 5 |
| PROK2 |  | 5 |
| RAD9B |  | 5 |
| RPSA |  | 5 |
| A1CF |  | 7 |
| CCL21 |  | 7 |
| CDHR2 |  | 7 |
| GNG8 |  | 7 |
| GRIK3 |  | 7 |
| KLRD1 |  | 7 |
| MAGEA5 |  | 7 |
| PCID2 |  | 7 |
| TMED7 |  | 7 |
| TUBB1 |  | 7 |
| ZMAT5 |  | 7 |
| COBL |  | 8 |
| DDX54 |  | 8 |
| DZANK1 |  | 8 |
| GPER |  | 8 |
| PRODH |  | 8 |
| ARHGEF10L |  | 9 |
| NEGR1 |  | 9 |
| PLIN3 |  | 9 |
| RPS6KA5 |  | 9 |
| UTS2R |  | 9 |
| C2CD4B |  | 11 |
| PNLDC1 |  | 11 |
| ZNF300 |  | 11 |
| C5orf20 |  | 12 |
| ERMAP |  | 12 |
| GOLGA4 |  | 12 |
| NR1H3 |  | 12 |
| WDR1 |  | 12 |
| ZNF280B |  | 12 |
| CNTF |  | 13 |
| F2RL1 |  | 13 |
| FOXD3 |  | 13 |
| MCM10 |  | 13 |
| MTCP1NB |  | 13 |
| PRAP1 |  | 13 |
| SOX12 |  | 13 |
| THAP11 |  | 13 |
| UBE2Z |  | 13 |
| APEX1 |  | 15 |
| COX6C |  | 15 |
| DSCC1 |  | 15 |
| LILRB1 |  | 15 |
| LRRC4C |  | 15 |
| MYL12B |  | 15 |
| TXNRD3NB |  | 15 |
| CD36 |  | 16 |
| DNAJC8 |  | 16 |
| HDHD1 |  | 16 |
| SPINK2 |  | 16 |
| GFRA2 |  | 17 |
| WDR76 |  | 17 |
| FRMD6 |  | 19 |
| INSL4 |  | 19 |
| KLRC1 |  | 19 |
| PEF1 |  | 19 |
| QRFPR |  | 19 |
| SARNP |  | 19 |
| SLC22A1 |  | 19 |
| REG3A |  | 20 |
| USP25 |  | 20 |
| ZNF718 |  | 20 |
| CDK16 |  | 21 |
| ITGB2 |  | 23 |
| MTERFD3 |  | 23 |
| DCDC2B |  | 24 |
| PCDHGB1 |  | 25 |
| FLOT1 |  | 27 |
| IFITM5 |  | 27 |
| MRPS23 |  | 27 |
| UBE2T |  | 27 |
| TRIM48 |  | 31 |
| PDS5A |  | 32 |
| FAM189A1 |  | 35 |
| SCLT1 |  | 40 |
| MCRS1 |  | 63 |
| CRYZ |  | 1 |
| KIR2DS2 |  | 1 |
| LYPD6 |  | 1 |
| AIPL1 |  | 3 |
| ERC1 |  | 3 |
| FAM63B |  | 3 |
| FDFT1 |  | 3 |
| GPATCH4 |  | 3 |
| MPEG1 |  | 3 |
| RAP2C |  | 3 |
| RPRD1A |  | 3 |
| TOR3A |  | 3 |
| ANAPC16 |  | 4 |
| AP1S3 |  | 4 |
| BOC |  | 4 |
| C12orf75 |  | 4 |
| DRG1 |  | 4 |
| EIF1B |  | 4 |
| IL4 |  | 4 |
| RAP2A |  | 4 |
| RRAGC |  | 4 |
| SGCB |  | 4 |
| SRD5A1 |  | 4 |
| TMLHE |  | 4 |
| TRPC3 |  | 4 |
| ZNF658 |  | 4 |
| ABHD12B |  | 5 |
| ALKBH7 |  | 5 |
| COPS6 |  | 5 |
| DGAT2L6 |  | 5 |
| GALNT12 |  | 5 |
| MPL |  | 5 |
| PANK1 |  | 5 |
| RALA |  | 5 |
| ROPN1B |  | 5 |
| SLC25A14 |  | 5 |
| TGIF2LY |  | 5 |
| ZNF19 |  | 5 |
| APCDD1 |  | 7 |
| C15orf43 |  | 7 |
| C2orf62 |  | 7 |
| DOCK5 |  | 7 |
| GATAD2B |  | 7 |
| NDUFA4L2 |  | 7 |
| AGXT2 |  | 8 |
| C16orf57 |  | 8 |
| DCTN1 |  | 8 |
| GOLM1 |  | 8 |
| KCTD11 |  | 8 |
| LCN1 |  | 8 |
| LDHC |  | 8 |
| PEX2 |  | 8 |
| PRDM7 |  | 8 |
| PVRL1 |  | 8 |
| WSCD2 |  | 8 |
| ZNF395 |  | 8 |
| HIST1H3D |  | 9 |
| IFT27 |  | 9 |
| KIAA1984 |  | 9 |
| NOBOX |  | 9 |
| PCDHGB5 |  | 9 |
| SLC25A27 |  | 9 |
| TNFSF11 |  | 9 |
| WDR54 |  | 9 |
| XPO5 |  | 9 |
| BIN2 |  | 11 |
| C5orf46 |  | 11 |
| CHST14 |  | 11 |
| CLEC2B |  | 11 |
| GGTLC1 |  | 11 |
| GPC4 |  | 11 |
| PHACTR1 |  | 11 |
| SLC12A8 |  | 11 |
| SOSTDC1 |  | 11 |
| TIMP2 |  | 11 |
| FGFR2 |  | 12 |
| RAB8B |  | 12 |
| TKTL2 |  | 12 |
| WDR6 |  | 12 |
| BCL7A |  | 13 |
| GNL3L |  | 13 |
| HP1BP3 |  | 13 |
| MEGF9 |  | 13 |
| OR6T1 |  | 13 |
| GTF2H3 |  | 15 |
| HAUS4 |  | 15 |
| MED24 |  | 15 |
| DEFA5 |  | 16 |
| RARS2 |  | 16 |
| RPS19BP1 |  | 16 |
| SCAP |  | 16 |
| TAS2R42 |  | 16 |
| TRAPPC12 |  | 16 |
| ARHGAP36 |  | 17 |
| GSTM2 |  | 17 |
| NOL10 |  | 17 |
| KIF2C |  | 19 |
| L3MBTL1 |  | 19 |
| TMBIM4 |  | 19 |
| ITGB8 |  | 21 |
| LCTL |  | 21 |
| INTS6 |  | 23 |
| SCARB1 |  | 23 |
| TMEM92 |  | 23 |
| ZNF558 |  | 23 |
| LRPAP1 |  | 24 |
| PRH2 |  | 24 |
| ZNF829 |  | 24 |
| CEACAM16 |  | 25 |
| RARRES2 |  | 28 |
| ARL2BP |  | 31 |
| OR10A2 |  | 31 |
| PPP3CB |  | 31 |
| RAPGEF5 |  | 32 |
| RGS18 |  | 32 |
| DHCR24 |  | 36 |
| DUSP5 |  | 43 |
| AP2M1 |  | 55 |
| ZNF35 |  | 61 |
| RYBP |  | 88 |
| C1orf222 |  | 1 |
| C1orf96 |  | 1 |
| CHI3L2 |  | 1 |
| HOGA1 |  | 1 |
| DCAF12 |  | 3 |
| FBXW12 |  | 3 |
| GK |  | 3 |
| TDRD9 |  | 3 |
| USP37 |  | 3 |
| ZNF396 |  | 3 |
| AANAT |  | 4 |
| ATP6V0D1 |  | 4 |
| BCS1L |  | 4 |
| COMTD1 |  | 4 |
| DUOXA1 |  | 4 |
| EIF2S2 |  | 4 |
| FCRL3 |  | 4 |
| IL13RA1 |  | 4 |
| PSMA5 |  | 4 |
| SNRPC |  | 4 |
| VANGL2 |  | 4 |
| VBP1 |  | 4 |
| ACSM3 |  | 5 |
| ATAD3C |  | 5 |
| CCNI |  | 5 |
| CD28 |  | 5 |
| GPR137B |  | 5 |
| IL21R |  | 5 |
| KLF6 |  | 5 |
| LSM7 |  | 5 |
| LTF |  | 5 |
| SAMD10 |  | 5 |
| SCPEP1 |  | 5 |
| TIRAP |  | 5 |
| ADCY2 |  | 7 |
| AK3 |  | 7 |
| CTBS |  | 7 |
| FAM53C |  | 7 |
| GBA3 |  | 7 |
| IFNA7 |  | 7 |
| IMPA2 |  | 7 |
| PDE7B |  | 7 |
| PLEK2 |  | 7 |
| RAB43 |  | 7 |
| THOC3 |  | 7 |
| DDT |  | 8 |
| LGR5 |  | 8 |
| LHFPL1 |  | 8 |
| MTPAP |  | 8 |
| RPL21 |  | 8 |
| RPP25L |  | 8 |
| TCEAL3 |  | 8 |
| USP29 |  | 8 |
| USP30 |  | 8 |
| HCFC1 |  | 9 |
| PNPLA4 |  | 9 |
| PPAP2A |  | 9 |
| PSMD10 |  | 9 |
| QARS |  | 9 |
| RAD51D |  | 9 |
| SLITRK3 |  | 9 |
| TMEM164 |  | 9 |
| VASH1 |  | 9 |
| WNT9B |  | 9 |
| ZNF223 |  | 9 |
| DNASE2 |  | 11 |
| HEPN1 |  | 11 |
| ISLR2 |  | 11 |
| LAMP3 |  | 11 |
| LMLN |  | 11 |
| NAGA |  | 11 |
| NUF2 |  | 11 |
| SAMD4B |  | 11 |
| SDHB |  | 11 |
| THPO |  | 11 |
| UGT1A4 |  | 11 |
| ANKDD1A |  | 12 |
| IL2RA |  | 12 |
| OR2T33 |  | 12 |
| SPATA8 |  | 12 |
| SSX5 |  | 12 |
| ATP5L2 |  | 13 |
| C7orf49 |  | 13 |
| CAPG |  | 13 |
| CSNK1A1 |  | 13 |
| FZD8 |  | 13 |
| MARCH9 |  | 13 |
| NLGN3 |  | 13 |
| PRR3 |  | 13 |
| RPL35 |  | 13 |
| TBC1D26 |  | 13 |
| TMEM189 |  | 13 |
| ANXA1 |  | 15 |
| GPBAR1 |  | 15 |
| OR13J1 |  | 15 |
| SLC39A3 |  | 15 |
| FCRL2 |  | 16 |
| HEATR5A |  | 16 |
| MAT1A |  | 16 |
| NTAN1 |  | 16 |
| OMG |  | 16 |
| C10orf62 |  | 17 |
| C17orf89 |  | 17 |
| C9orf11 |  | 17 |
| DEFB103B |  | 17 |
| EIF3F |  | 17 |
| GFRA3 |  | 17 |
| RAB33B |  | 17 |
| C16orf7 |  | 19 |
| USP8 |  | 19 |
| BMS1 |  | 20 |
| DCAKD |  | 21 |
| EIF3E |  | 21 |
| USP16 |  | 21 |
| FAM134B |  | 23 |
| INSL5 |  | 23 |
| LYG2 |  | 23 |
| XKR6 |  | 23 |
| HNRNPA2B1 |  | 24 |
| PTHLH |  | 24 |
| CYP26A1 |  | 25 |
| ZNF529 |  | 25 |
| AZGP1 |  | 27 |
| NDUFB3 |  | 27 |
| B4GALT4 |  | 29 |
| DARC |  | 29 |
| VEGFA |  | 31 |
| RGS3 |  | 32 |
| TSGA10IP |  | 32 |
| ZFC3H1 |  | 36 |
| CDX1 |  | 39 |
| ZNF777 |  | 39 |
| SIX1 |  | 41 |
| ANKRD44 |  | 43 |
| CIRBP |  | 44 |
| PMEPA1 |  | 44 |
| HOXC5 |  | 57 |
| E4F1 |  | 59 |
| BAGE4 |  | 1 |
| C1orf64 |  | 1 |
| DTNBP1 |  | 1 |
| OMA1 |  | 1 |
| ANKRD20A3 |  | 3 |
| ARHGAP25 |  | 3 |
| COQ5 |  | 3 |
| DNHD1 |  | 3 |
| FAM50A |  | 3 |
| GNRHR |  | 3 |
| HOPX |  | 3 |
| IRAK3 |  | 3 |
| NCL |  | 3 |
| SMCP |  | 3 |
| TBXAS1 |  | 3 |
| TGDS |  | 3 |
| TIMMDC1 |  | 3 |
| TMEM123 |  | 3 |
| B4GALT5 |  | 4 |
| BPIFB4 |  | 4 |
| ENOSF1 |  | 4 |
| EPHX3 |  | 4 |
| GRTP1 |  | 4 |
| GTF2B |  | 4 |
| KCNMB4 |  | 4 |
| LYPD4 |  | 4 |
| MAP2K5 |  | 4 |
| MRGPRX4 |  | 4 |
| OR13C9 |  | 4 |
| PCDHB5 |  | 4 |
| SCUBE1 |  | 4 |
| SDHC |  | 4 |
| TRPS1 |  | 4 |
| CYP3A4 |  | 5 |
| DCAF15 |  | 5 |
| DCAF7 |  | 5 |
| DMWD |  | 5 |
| EIF4E |  | 5 |
| FAM26E |  | 5 |
| HBG1 |  | 5 |
| PCDHB6 |  | 5 |
| RFPL4B |  | 5 |
| RNASE13 |  | 5 |
| RPS15A |  | 5 |
| SHOC2 |  | 5 |
| ST3GAL3 |  | 5 |
| TADA1 |  | 5 |
| TNFRSF9 |  | 5 |
| TRMT12 |  | 5 |
| UBE3C |  | 5 |
| ZMYND11 |  | 5 |
| ADCK5 |  | 7 |
| C17orf67 |  | 7 |
| C1RL |  | 7 |
| C5orf60 |  | 7 |
| CENPW |  | 7 |
| FHL1 |  | 7 |
| LDHD |  | 7 |
| LOXL3 |  | 7 |
| RHBDL1 |  | 7 |
| TMEM82 |  | 7 |
| ZDHHC8 |  | 7 |
| BPY2 |  | 8 |
| CA1 |  | 8 |
| FGL1 |  | 8 |
| KIAA1143 |  | 8 |
| LRTM2 |  | 8 |
| PCDHGC5 |  | 8 |
| PTH1R |  | 8 |
| TBC1D3C |  | 8 |
| UQCR10 |  | 8 |
| ZSCAN20 |  | 8 |
| ALOX15 |  | 9 |
| CALCRL |  | 9 |
| EPT1 |  | 9 |
| MIPEP |  | 9 |
| TTC9C |  | 9 |
| VHL |  | 9 |
| ZC2HC1C |  | 9 |
| ZNF546 |  | 9 |
| CA6 |  | 11 |
| CHRNA3 |  | 11 |
| DPPA5 |  | 11 |
| FAM102B |  | 11 |
| FOLH1B |  | 11 |
| GNLY |  | 11 |
| INSL3 |  | 11 |
| ORAOV1 |  | 11 |
| PTP4A3 |  | 11 |
| SUMF2 |  | 11 |
| TNFRSF1A |  | 11 |
| YPEL3 |  | 11 |
| ZNF131 |  | 11 |
| ANGPTL4 |  | 12 |
| CHGA |  | 12 |
| CXorf48 |  | 12 |
| DAD1 |  | 12 |
| EDF1 |  | 12 |
| FAM181A |  | 12 |
| GPR85 |  | 12 |
| LIMS3L |  | 12 |
| MTUS1 |  | 12 |
| SMPDL3B |  | 12 |
| BSCL2 |  | 13 |
| ECD |  | 13 |
| GLDC |  | 13 |
| MAGED2 |  | 13 |
| OLIG3 |  | 13 |
| PDIK1L |  | 13 |
| PPIAL4B |  | 13 |
| PPM1B |  | 13 |
| PTPN9 |  | 13 |
| RPS11 |  | 13 |
| TRAPPC5 |  | 13 |
| ZNF774 |  | 13 |
| HDC |  | 15 |
| KAL1 |  | 15 |
| KCNC4 |  | 15 |
| LPGAT1 |  | 15 |
| MAP3K6 |  | 15 |
| NLRP7 |  | 15 |
| PPIL4 |  | 15 |
| ZNF287 |  | 15 |
| 40422 |  | 16 |
| BTNL9 |  | 16 |
| C2orf63 |  | 16 |
| GAB2 |  | 16 |
| IFT46 |  | 16 |
| SLC30A5 |  | 16 |
| CCDC83 |  | 17 |
| RPL8 |  | 17 |
| SKP2 |  | 17 |
| TGIF2LX |  | 17 |
| THAP10 |  | 17 |
| KPNA2 |  | 19 |
| NYNRIN |  | 19 |
| PLA2G2D |  | 19 |
| FAM209A |  | 20 |
| MPLKIP |  | 20 |
| SPANXC |  | 20 |
| ZNF414 |  | 20 |
| ART3 |  | 21 |
| EFHA1 |  | 21 |
| GLIPR1L2 |  | 21 |
| PODNL1 |  | 21 |
| PPP1R12B |  | 21 |
| PAPD5 |  | 23 |
| ZNF3 |  | 23 |
| C12orf59 |  | 24 |
| FAM19A5 |  | 25 |
| PRDX3 |  | 25 |
| RCE1 |  | 28 |
| C7orf31 |  | 31 |
| HSFY2 |  | 31 |
| RGS5 |  | 31 |
| UBD |  | 31 |
| CEMP1 |  | 32 |
| KLF12 |  | 33 |
| SPANXA1 |  | 35 |
| TNNI3 |  | 37 |
| HSD17B7 |  | 39 |
| HUS1 |  | 39 |
| ZSCAN4 |  | 39 |
| DUPD1 |  | 43 |
| ZNF410 |  | 43 |
| OR7C1 |  | 1 |
| ATP12A |  | 3 |
| C7orf26 |  | 3 |
| C9orf123 |  | 3 |
| CDKL5 |  | 3 |
| DCUN1D2 |  | 3 |
| ENTPD7 |  | 3 |
| IL3RA |  | 3 |
| MRPS11 |  | 3 |
| MRPS18C |  | 3 |
| OAZ3 |  | 3 |
| PCBP3 |  | 3 |
| RTN4 |  | 3 |
| SLC25A29 |  | 3 |
| SPAG6 |  | 3 |
| TMEM180 |  | 3 |
| ACTR6 |  | 4 |
| BEX4 |  | 4 |
| C1orf21 |  | 4 |
| CCDC142 |  | 4 |
| COL23A1 |  | 4 |
| EML3 |  | 4 |
| FCN1 |  | 4 |
| FGF4 |  | 4 |
| HLA-DRB5 |  | 4 |
| IL2 |  | 4 |
| INF2 |  | 4 |
| IST1 |  | 4 |
| NPTX2 |  | 4 |
| NTRK3 |  | 4 |
| OSTN |  | 4 |
| RFTN1 |  | 4 |
| SDHAF1 |  | 4 |
| TAF13 |  | 4 |
| TUBG2 |  | 4 |
| C19orf53 |  | 5 |
| CCDC90B |  | 5 |
| CHRDL2 |  | 5 |
| CHRM3 |  | 5 |
| CPNE5 |  | 5 |
| CT45A3 |  | 5 |
| DHDH |  | 5 |
| FLT1 |  | 5 |
| IFNA16 |  | 5 |
| IL17RD |  | 5 |
| LIX1L |  | 5 |
| MRPS5 |  | 5 |
| NR2E3 |  | 5 |
| SF3B3 |  | 5 |
| SLC5A10 |  | 5 |
| SLCO1A2 |  | 5 |
| TNS4 |  | 5 |
| USP36 |  | 5 |
| ZNF562 |  | 5 |
| ADI1 |  | 7 |
| ATG9B |  | 7 |
| BTG1 |  | 7 |
| DNAJB3 |  | 7 |
| SLC10A1 |  | 7 |
| TULP1 |  | 7 |
| VWA2 |  | 7 |
| APH1A |  | 8 |
| ARPC5L |  | 8 |
| ASXL2 |  | 8 |
| C20orf72 |  | 8 |
| COMMD5 |  | 8 |
| FGB |  | 8 |
| H2BFWT |  | 8 |
| HMG20B |  | 8 |
| LIG3 |  | 8 |
| MANSC1 |  | 8 |
| PCDHA11 |  | 8 |
| SLC2A11 |  | 8 |
| SNX31 |  | 8 |
| STK19 |  | 8 |
| TAX1BP3 |  | 8 |
| TMEM241 |  | 8 |
| C1QA |  | 9 |
| CCL5 |  | 9 |
| CIB2 |  | 9 |
| DHH |  | 9 |
| GJA5 |  | 9 |
| OR5AK2 |  | 9 |
| OXTR |  | 9 |
| RSBN1 |  | 9 |
| FAM19A1 |  | 11 |
| FCHSD2 |  | 11 |
| IGFBP2 |  | 11 |
| PXT1 |  | 11 |
| TPGS2 |  | 11 |
| ZBTB6 |  | 11 |
| ZC3HC1 |  | 11 |
| MYL2 |  | 12 |
| NKAIN1 |  | 12 |
| PTPRR |  | 12 |
| SEMA3D |  | 12 |
| SPDYA |  | 12 |
| C19orf45 |  | 13 |
| CEP85 |  | 13 |
| F3 |  | 13 |
| MRPS31 |  | 13 |
| SEC31A |  | 13 |
| SLC9B1 |  | 13 |
| VSTM2A |  | 13 |
| ZNF673 |  | 13 |
| APOH |  | 15 |
| ARMC1 |  | 15 |
| DNAL1 |  | 15 |
| HSPD1 |  | 15 |
| MMP7 |  | 15 |
| SFTPA2 |  | 15 |
| ACOT4 |  | 16 |
| ADIPOR2 |  | 16 |
| DNAJC15 |  | 16 |
| HOXD12 |  | 16 |
| OR4F15 |  | 16 |
| PMEL |  | 16 |
| UCN3 |  | 16 |
| RAB11B |  | 17 |
| SLC5A2 |  | 17 |
| CATSPER2 |  | 19 |
| RPL7 |  | 19 |
| VPS53 |  | 19 |
| CLEC12A |  | 20 |
| IL6R |  | 20 |
| POLR2C |  | 20 |
| SCIMP |  | 20 |
| ZNF653 |  | 20 |
| AKTIP |  | 21 |
| DEFB115 |  | 21 |
| DLK1 |  | 21 |
| FA2H |  | 21 |
| FIGNL1 |  | 21 |
| PRSS35 |  | 21 |
| RALGPS2 |  | 21 |
| RPN2 |  | 21 |
| RUVBL1 |  | 21 |
| SEMA6C |  | 21 |
| TRIM61 |  | 21 |
| TTC33 |  | 21 |
| ARSK |  | 23 |
| LETM2 |  | 23 |
| CD180 |  | 24 |
| FGF19 |  | 24 |
| LRRC37B |  | 24 |
| MYL4 |  | 24 |
| ZNF784 |  | 24 |
| ABCA8 |  | 25 |
| CHMP1B |  | 25 |
| LSMD1 |  | 25 |
| SH3RF2 |  | 25 |
| PPP1R36 |  | 27 |
| UGT3A1 |  | 27 |
| NAA40 |  | 29 |
| C6orf25 |  | 31 |
| CLEC14A |  | 33 |
| CYYR1 |  | 33 |
| ZACN |  | 33 |
| HLA-G |  | 35 |
| SPPL3 |  | 35 |
| PAX7 |  | 36 |
| RNF6 |  | 40 |
| MTCH1 |  | 41 |
| STK35 |  | 61 |
| C16orf42 |  | 1 |
| C8orf74 |  | 1 |
| CAMKK1 |  | 1 |
| CYB5D2 |  | 1 |
| DMGDH |  | 1 |
| FAM217B |  | 1 |
| GABRA1 |  | 1 |
| HIST1H2AE |  | 1 |
| IDI1 |  | 1 |
| PGAM2 |  | 1 |
| SRFBP1 |  | 1 |
| SYNPO2L |  | 1 |
| BFAR |  | 3 |
| CYP3A7 |  | 3 |
| DNAJB2 |  | 3 |
| IL28A |  | 3 |
| IMP3 |  | 3 |
| KIAA0355 |  | 3 |
| MCOLN1 |  | 3 |
| MMP26 |  | 3 |
| MORN5 |  | 3 |
| OXA1L |  | 3 |
| PANK4 |  | 3 |
| PRSS57 |  | 3 |
| RBM7 |  | 3 |
| SAMD4A |  | 3 |
| SEZ6 |  | 3 |
| STARD13 |  | 3 |
| STARD5 |  | 3 |
| UBA7 |  | 3 |
| UGT1A9 |  | 3 |
| ZNF260 |  | 3 |
| ZNF689 |  | 3 |
| ACAA2 |  | 4 |
| B2M |  | 4 |
| BCL2L11 |  | 4 |
| C16orf45 |  | 4 |
| C20orf4 |  | 4 |
| C22orf23 |  | 4 |
| C9orf169 |  | 4 |
| CHCHD1 |  | 4 |
| DCAF5 |  | 4 |
| FADD |  | 4 |
| FAM163B |  | 4 |
| FAM81A |  | 4 |
| FOLR3 |  | 4 |
| FZD7 |  | 4 |
| GIMAP4 |  | 4 |
| HLA-DRB1 |  | 4 |
| PLCXD3 |  | 4 |
| PLRG1 |  | 4 |
| RNF25 |  | 4 |
| SOCS5 |  | 4 |
| SUV39H2 |  | 4 |
| TCF21 |  | 4 |
| TPT1 |  | 4 |
| ZNF584 |  | 4 |
| ANXA2R |  | 5 |
| BLVRA |  | 5 |
| C5orf54 |  | 5 |
| CC2D1B |  | 5 |
| COL21A1 |  | 5 |
| COL2A1 |  | 5 |
| DCP2 |  | 5 |
| DEFB131 |  | 5 |
| DSCR3 |  | 5 |
| ERLIN2 |  | 5 |
| FOLR2 |  | 5 |
| KYNU |  | 5 |
| MAD2L1 |  | 5 |
| NDUFA4 |  | 5 |
| NECAP2 |  | 5 |
| OMP |  | 5 |
| PIGB |  | 5 |
| PRL |  | 5 |
| RPL36AL |  | 5 |
| RPS29 |  | 5 |
| SPINK7 |  | 5 |
| SUPV3L1 |  | 5 |
| TXNDC12 |  | 5 |
| UQCC |  | 5 |
| ZNF225 |  | 5 |
| ZNF518A |  | 5 |
| ALG1L |  | 7 |
| ASPRV1 |  | 7 |
| C22orf26 |  | 7 |
| CASP4 |  | 7 |
| CDK5RAP1 |  | 7 |
| CENPI |  | 7 |
| HIST1H2AA |  | 7 |
| LGI1 |  | 7 |
| LRRC14B |  | 7 |
| MANBAL |  | 7 |
| METTL2A |  | 7 |
| NPAS3 |  | 7 |
| NT5DC1 |  | 7 |
| ORC4 |  | 7 |
| POLR2G |  | 7 |
| PPP1R14D |  | 7 |
| RDH5 |  | 7 |
| SURF2 |  | 7 |
| TBC1D7 |  | 7 |
| TIGD3 |  | 7 |
| 37500 |  | 8 |
| C12orf52 |  | 8 |
| CCDC93 |  | 8 |
| CHMP7 |  | 8 |
| EDEM3 |  | 8 |
| GUCY1B3 |  | 8 |
| HRASLS2 |  | 8 |
| MAFB |  | 8 |
| NANOS3 |  | 8 |
| PDE6G |  | 8 |
| POLE3 |  | 8 |
| RMI2 |  | 8 |
| SLC52A2 |  | 8 |
| UBE2V2 |  | 8 |
| VIP |  | 8 |
| C10orf91 |  | 9 |
| CALM1 |  | 9 |
| GFM2 |  | 9 |
| GTF2F2 |  | 9 |
| HIST1H4A |  | 9 |
| KCND2 |  | 9 |
| KCNG4 |  | 9 |
| MED22 |  | 9 |
| MICAL3 |  | 9 |
| PILRB |  | 9 |
| PTN |  | 9 |
| RRAGA |  | 9 |
| SLC16A4 |  | 9 |
| ZNF75D |  | 9 |
| ACTR1B |  | 11 |
| ANGEL1 |  | 11 |
| C11orf45 |  | 11 |
| C5orf62 |  | 11 |
| CDC26 |  | 11 |
| CNGA3 |  | 11 |
| COMMD4 |  | 11 |
| ECHDC2 |  | 11 |
| KRTAP13-2 |  | 11 |
| MYO10 |  | 11 |
| PCDHGA6 |  | 11 |
| SLC5A1 |  | 11 |
| SPRED1 |  | 11 |
| STK40 |  | 11 |
| TBC1D2 |  | 11 |
| TCEA3 |  | 11 |
| TMEM88 |  | 11 |
| XYLT1 |  | 11 |
| C7orf45 |  | 12 |
| DPM3 |  | 12 |
| EME2 |  | 12 |
| GNA11 |  | 12 |
| KRCC1 |  | 12 |
| ZNF200 |  | 12 |
| C11orf52 |  | 13 |
| DHX16 |  | 13 |
| ERCC1 |  | 13 |
| MAGEB2 |  | 13 |
| MIER1 |  | 13 |
| RBM15 |  | 13 |
| TMED4 |  | 13 |
| C19orf57 |  | 15 |
| HIST1H3I |  | 15 |
| HTRA4 |  | 15 |
| INPP4A |  | 15 |
| LRRC49 |  | 15 |
| NSMCE1 |  | 15 |
| OR8H1 |  | 15 |
| RSPH10B2 |  | 15 |
| TNFRSF18 |  | 15 |
| AMIGO1 |  | 16 |
| C17orf50 |  | 16 |
| GLP2R |  | 16 |
| HECTD2 |  | 16 |
| LRTOMT |  | 16 |
| PENK |  | 16 |
| SUMO3 |  | 16 |
| VPS16 |  | 16 |
| ZFP57 |  | 16 |
| ZNF704 |  | 16 |
| ADSSL1 |  | 17 |
| C7orf63 |  | 17 |
| CACNB4 |  | 17 |
| NDUFA13 |  | 17 |
| OR52I2 |  | 17 |
| RAB11A |  | 17 |
| SLC8A3 |  | 17 |
| TTLL6 |  | 17 |
| ZNF695 |  | 17 |
| FOXR1 |  | 19 |
| GALNT3 |  | 19 |
| NPFF |  | 19 |
| POLR1D |  | 19 |
| SPC25 |  | 19 |
| SPRR2B |  | 19 |
| C2orf50 |  | 20 |
| DCLRE1C |  | 20 |
| FBXO5 |  | 20 |
| GAGE2E |  | 20 |
| MRPL4 |  | 20 |
| PAPOLA |  | 20 |
| SAMD12 |  | 20 |
| TAS2R14 |  | 20 |
| B4GALT1 |  | 21 |
| C14orf37 |  | 21 |
| RASGEF1C |  | 21 |
| TCL1B |  | 21 |
| LNX1 |  | 23 |
| RPS19 |  | 23 |
| BRCA1 |  | 24 |
| CRISPLD2 |  | 24 |
| KLK11 |  | 24 |
| CD22 |  | 25 |
| CERCAM |  | 27 |
| ASB6 |  | 28 |
| BRK1 |  | 28 |
| RPL13 |  | 28 |
| WFDC5 |  | 28 |
| FOXD4L2 |  | 29 |
| MRPL10 |  | 29 |
| NDNL2 |  | 29 |
| PLEKHG6 |  | 29 |
| ADAMTS18 |  | 31 |
| BOD1L2 |  | 31 |
| HMGN2 |  | 31 |
| EPN3 |  | 32 |
| PRRX1 |  | 32 |
| ARL13A |  | 33 |
| OSM |  | 35 |
| TMEM144 |  | 35 |
| DLX5 |  | 44 |
| SERBP1 |  | 47 |
| ZNF74 |  | 49 |
| LMX1B |  | 53 |
| UBXN1 |  | 53 |
| MYPOP |  | 73 |
| ARHGEF9 |  | 1 |
| BOK |  | 1 |
| C11orf1 |  | 1 |
| C15orf63 |  | 1 |
| CCDC146 |  | 1 |
| CHAMP1 |  | 1 |
| CSDC2 |  | 1 |
| CTAG1A |  | 1 |
| CYP3A5 |  | 1 |
| DHFR |  | 1 |
| EZH2 |  | 1 |
| LYSMD2 |  | 1 |
| RPL4 |  | 1 |
| SLC38A11 |  | 1 |
| SLC6A7 |  | 1 |
| TCEA1 |  | 1 |
| TPPP2 |  | 1 |
| ZNF654 |  | 1 |
| ZPBP2 |  | 1 |
| AGFG2 |  | 3 |
| AMZ1 |  | 3 |
| ASB9 |  | 3 |
| C16orf80 |  | 3 |
| C4orf27 |  | 3 |
| C9orf43 |  | 3 |
| CBY1 |  | 3 |
| CCDC167 |  | 3 |
| CCDC54 |  | 3 |
| FAM218A |  | 3 |
| FAM83F |  | 3 |
| GPN2 |  | 3 |
| GTF2IRD2 |  | 3 |
| KIAA0141 |  | 3 |
| MYOZ2 |  | 3 |
| PTS |  | 3 |
| RAD51B |  | 3 |
| SNAPC3 |  | 3 |
| TLE1 |  | 3 |
| TOM1 |  | 3 |
| UBE2L3 |  | 3 |
| ZNF607 |  | 3 |
| ABHD10 |  | 4 |
| ASTE1 |  | 4 |
| ATPIF1 |  | 4 |
| AUH |  | 4 |
| C1orf146 |  | 4 |
| CPA3 |  | 4 |
| DYRK4 |  | 4 |
| EIF2C3 |  | 4 |
| FBXW9 |  | 4 |
| FRRS1 |  | 4 |
| GABRG2 |  | 4 |
| GDF15 |  | 4 |
| GPLD1 |  | 4 |
| GPR89A |  | 4 |
| HEATR1 |  | 4 |
| HHIP |  | 4 |
| HTR2C |  | 4 |
| IDH3B |  | 4 |
| IFNA5 |  | 4 |
| IL17A |  | 4 |
| INSL6 |  | 4 |
| MRPS18B |  | 4 |
| OR2T10 |  | 4 |
| PARVG |  | 4 |
| PCDHA3 |  | 4 |
| PDE7A |  | 4 |
| PRSS37 |  | 4 |
| SERPINA9 |  | 4 |
| SNX22 |  | 4 |
| A1BG |  | 5 |
| ACVR1 |  | 5 |
| ADH7 |  | 5 |
| ANTXR1 |  | 5 |
| ANXA13 |  | 5 |
| BMP2 |  | 5 |
| BRD3 |  | 5 |
| CD14 |  | 5 |
| CSAG1 |  | 5 |
| DHRS11 |  | 5 |
| DKKL1 |  | 5 |
| DPYSL4 |  | 5 |
| DYNC1I2 |  | 5 |
| GFRA4 |  | 5 |
| HEPACAM2 |  | 5 |
| LARS |  | 5 |
| MRGPRD |  | 5 |
| OAZ1 |  | 5 |
| OSCP1 |  | 5 |
| P4HB |  | 5 |
| PAPSS2 |  | 5 |
| PSG9 |  | 5 |
| RAD51AP1 |  | 5 |
| SCGB2A2 |  | 5 |
| SFN |  | 5 |
| SLC23A3 |  | 5 |
| SLC35A3 |  | 5 |
| SLCO2B1 |  | 5 |
| THAP8 |  | 5 |
| TREH |  | 5 |
| TRPM8 |  | 5 |
| UFC1 |  | 5 |
| WT1 |  | 5 |
| ATP5G2 |  | 7 |
| C12orf57 |  | 7 |
| C17orf75 |  | 7 |
| C8orf59 |  | 7 |
| DDX41 |  | 7 |
| DEFA3 |  | 7 |
| DHRS9 |  | 7 |
| DNAJC10 |  | 7 |
| EIF3H |  | 7 |
| GML |  | 7 |
| HPS6 |  | 7 |
| IL36RN |  | 7 |
| IQCB1 |  | 7 |
| LIM2 |  | 7 |
| MAN2B1 |  | 7 |
| MTHFD1L |  | 7 |
| PBX4 |  | 7 |
| PHLDB1 |  | 7 |
| PIP |  | 7 |
| PLGLB2 |  | 7 |
| POTEC |  | 7 |
| PPBP |  | 7 |
| PPIAL4G |  | 7 |
| PPIG |  | 7 |
| PSORS1C1 |  | 7 |
| PTGDR |  | 7 |
| RBP4 |  | 7 |
| RSG1 |  | 7 |
| SFTA2 |  | 7 |
| UPF3A |  | 7 |
| ATP2A3 |  | 8 |
| C7orf29 |  | 8 |
| DNAAF2 |  | 8 |
| FAM109B |  | 8 |
| GLYATL2 |  | 8 |
| IFI27L1 |  | 8 |
| KCNJ9 |  | 8 |
| KIAA1737 |  | 8 |
| LY6G6D |  | 8 |
| NUSAP1 |  | 8 |
| PTGFRN |  | 8 |
| SLC46A2 |  | 8 |
| SPATA2 |  | 8 |
| SUCLG1 |  | 8 |
| ZFAND6 |  | 8 |
| ACYP2 |  | 9 |
| AMY1A |  | 9 |
| ASRGL1 |  | 9 |
| C1QTNF9 |  | 9 |
| GEMIN8 |  | 9 |
| GSDMC |  | 9 |
| KRT85 |  | 9 |
| MAP1LC3C |  | 9 |
| MC2R |  | 9 |
| MKI67IP |  | 9 |
| MOS |  | 9 |
| MRO |  | 9 |
| NDUFB6 |  | 9 |
| OR10AG1 |  | 9 |
| RNF166 |  | 9 |
| TAGLN2 |  | 9 |
| THAP6 |  | 9 |
| ZNF280A |  | 9 |
| BRF2 |  | 11 |
| C12orf49 |  | 11 |
| CCND1 |  | 11 |
| CCP110 |  | 11 |
| ENTPD2 |  | 11 |
| GDF11 |  | 11 |
| GREM2 |  | 11 |
| IFNA6 |  | 11 |
| NRN1L |  | 11 |
| PSMB6 |  | 11 |
| SCAMP1 |  | 11 |
| UPK3B |  | 11 |
| ZNF549 |  | 11 |
| ADAM18 |  | 12 |
| AKIP1 |  | 12 |
| ARHGEF16 |  | 12 |
| CHRNB2 |  | 12 |
| COPZ2 |  | 12 |
| MPDU1 |  | 12 |
| OR1L3 |  | 12 |
| PRRG4 |  | 12 |
| RNF115 |  | 12 |
| SLC30A10 |  | 12 |
| SUCLG2 |  | 12 |
| WFDC3 |  | 12 |
| BOD1 |  | 13 |
| C20orf132 |  | 13 |
| CHIT1 |  | 13 |
| FAM54B |  | 13 |
| FAM57A |  | 13 |
| GORAB |  | 13 |
| HTR3B |  | 13 |
| SIRPA |  | 13 |
| SLC35D3 |  | 13 |
| SLC5A9 |  | 13 |
| TMEM205 |  | 13 |
| AGPAT3 |  | 15 |
| C3orf23 |  | 15 |
| CWC15 |  | 15 |
| DPEP2 |  | 15 |
| FCGR3B |  | 15 |
| GOLGA8A |  | 15 |
| GPR12 |  | 15 |
| GPX8 |  | 15 |
| GRIN3B |  | 15 |
| HGF |  | 15 |
| HIST1H4B |  | 15 |
| LMBRD1 |  | 15 |
| MOGAT2 |  | 15 |
| OR1N1 |  | 15 |
| SAV1 |  | 15 |
| TIMM8A |  | 15 |
| TRPC5 |  | 15 |
| ZNF512B |  | 15 |
| AMDHD1 |  | 16 |
| CERS4 |  | 16 |
| CPLX1 |  | 16 |
| FUT3 |  | 16 |
| LRRC20 |  | 16 |
| POLR3D |  | 16 |
| SYT11 |  | 16 |
| TRIM41 |  | 16 |
| ACD |  | 17 |
| ADD3 |  | 17 |
| ARL17A |  | 17 |
| ATP6V1G3 |  | 17 |
| C12orf60 |  | 17 |
| C7orf59 |  | 17 |
| CA9 |  | 17 |
| CDC6 |  | 17 |
| EPHA7 |  | 17 |
| SERAC1 |  | 17 |
| SHISA5 |  | 17 |
| XRRA1 |  | 17 |
| BTNL8 |  | 19 |
| C20orf57 |  | 19 |
| CXCR6 |  | 19 |
| PDLIM1 |  | 19 |
| SCRN3 |  | 19 |
| UTP15 |  | 19 |
| ARMC10 |  | 20 |
| CHCHD2 |  | 20 |
| COQ9 |  | 20 |
| MTNR1A |  | 20 |
| TMEM120A |  | 20 |
| TRAPPC10 |  | 20 |
| DUS1L |  | 21 |
| PIAS3 |  | 21 |
| SNIP1 |  | 21 |
| SOWAHB |  | 21 |
| ZNF230 |  | 21 |
| CBX6 |  | 23 |
| FOXN4 |  | 23 |
| PNKD |  | 23 |
| COG1 |  | 24 |
| PRKRIR |  | 24 |
| PYCR1 |  | 24 |
| RPL15 |  | 24 |
| SYCN |  | 24 |
| ZSCAN2 |  | 24 |
| B3GALNT1 |  | 25 |
| SOCS1 |  | 25 |
| CD247 |  | 27 |
| SECISBP2 |  | 27 |
| GC |  | 28 |
| RBFA |  | 28 |
| SSX2B |  | 28 |
| PCDHGB2 |  | 31 |
| CPLX3 |  | 32 |
| ORC6 |  | 32 |
| ZNF655 |  | 32 |
| FAM136A |  | 33 |
| ZNF556 |  | 35 |
| NHLH2 |  | 36 |
| RUFY4 |  | 37 |
| CDC73 |  | 41 |
| PPM1L |  | 43 |
| ANKRD27 |  | 48 |
| ISL2 |  | 65 |
| GPX6 |  | 67 |
| WASL |  | 91 |
| ACTB |  | 1 |
| ACTRT1 |  | 1 |
| ANKRD23 |  | 1 |
| APRT |  | 1 |
| ARFIP2 |  | 1 |
| ATP6V1F |  | 1 |
| C1orf158 |  | 1 |
| C1QTNF1 |  | 1 |
| CCDC34 |  | 1 |
| CGRRF1 |  | 1 |
| CORO1A |  | 1 |
| CRTC1 |  | 1 |
| CSAG3 |  | 1 |
| FAM194A |  | 1 |
| GAGE1 |  | 1 |
| GFPT2 |  | 1 |
| GSTM1 |  | 1 |
| GUCA2B |  | 1 |
| HORMAD2 |  | 1 |
| HTN3 |  | 1 |
| LAMTOR3 |  | 1 |
| LEPRE1 |  | 1 |
| MECP2 |  | 1 |
| MIB2 |  | 1 |
| NUPL1 |  | 1 |
| PARS2 |  | 1 |
| PXDNL |  | 1 |
| RFESD |  | 1 |
| RSPH4A |  | 1 |
| SLC30A6 |  | 1 |
| SLCO1B1 |  | 1 |
| SPACA4 |  | 1 |
| SPON1 |  | 1 |
| THAP4 |  | 1 |
| THUMPD1 |  | 1 |
| TRIP11 |  | 1 |
| XRCC6 |  | 1 |
| ZNF778 |  | 1 |
| ACRV1 |  | 3 |
| AGAP3 |  | 3 |
| ANGPTL2 |  | 3 |
| ANKS1B |  | 3 |
| ATAD3B |  | 3 |
| ATP6V1B2 |  | 3 |
| C10orf140 |  | 3 |
| C12orf74 |  | 3 |
| CNNM1 |  | 3 |
| CRYZL1 |  | 3 |
| DKK1 |  | 3 |
| DPF3 |  | 3 |
| ECHS1 |  | 3 |
| ENC1 |  | 3 |
| FCGR1B |  | 3 |
| GLUD2 |  | 3 |
| HERC6 |  | 3 |
| HIST1H2AI |  | 3 |
| HJURP |  | 3 |
| IL1RAPL2 |  | 3 |
| KCTD19 |  | 3 |
| LBP |  | 3 |
| LSM4 |  | 3 |
| MED23 |  | 3 |
| MTX2 |  | 3 |
| NDUFA11 |  | 3 |
| NREP |  | 3 |
| PGA3 |  | 3 |
| PIGA |  | 3 |
| PPP1R14A |  | 3 |
| QPCT |  | 3 |
| RAMP1 |  | 3 |
| RSAD2 |  | 3 |
| SF3B2 |  | 3 |
| SLC39A7 |  | 3 |
| SLC41A3 |  | 3 |
| SLITRK6 |  | 3 |
| SPRR2A |  | 3 |
| SPRR2D |  | 3 |
| STYXL1 |  | 3 |
| TAS1R2 |  | 3 |
| TLR5 |  | 3 |
| YKT6 |  | 3 |
| ZNF10 |  | 3 |
| ZNF277 |  | 3 |
| ZNF420 |  | 3 |
| ABCD1 |  | 4 |
| AHSA2 |  | 4 |
| BMP4 |  | 4 |
| BTBD16 |  | 4 |
| CHST15 |  | 4 |
| CHST8 |  | 4 |
| CKAP5 |  | 4 |
| DHRS13 |  | 4 |
| ERAS |  | 4 |
| FADS2 |  | 4 |
| FSD1 |  | 4 |
| FTSJ1 |  | 4 |
| GPR132 |  | 4 |
| HDLBP |  | 4 |
| HLA-DOA |  | 4 |
| HSD11B1 |  | 4 |
| KLRF1 |  | 4 |
| LARP4B |  | 4 |
| MORC2 |  | 4 |
| MRPL39 |  | 4 |
| MSL2 |  | 4 |
| MUC20 |  | 4 |
| NHLRC1 |  | 4 |
| OLA1 |  | 4 |
| PARP11 |  | 4 |
| PCDHGC4 |  | 4 |
| PCSK2 |  | 4 |
| PDCD1LG2 |  | 4 |
| PRKACG |  | 4 |
| PRSS1 |  | 4 |
| PSMD4 |  | 4 |
| RNASET2 |  | 4 |
| RSRC1 |  | 4 |
| SGOL2 |  | 4 |
| SGSM3 |  | 4 |
| SLC25A37 |  | 4 |
| ST3GAL1 |  | 4 |
| STC2 |  | 4 |
| TNP2 |  | 4 |
| TOR2A |  | 4 |
| TRAIP |  | 4 |
| TUT1 |  | 4 |
| TWF1 |  | 4 |
| WDR18 |  | 4 |
| XAGE2 |  | 4 |
| ZFP41 |  | 4 |
| ZNF683 |  | 4 |
| ARID5B |  | 5 |
| BLID |  | 5 |
| BRS3 |  | 5 |
| BZW1 |  | 5 |
| C2orf66 |  | 5 |
| CAPN5 |  | 5 |
| CCNH |  | 5 |
| CDH24 |  | 5 |
| CLEC3A |  | 5 |
| CPA4 |  | 5 |
| CPNE8 |  | 5 |
| CSF1 |  | 5 |
| DKK4 |  | 5 |
| EXOSC4 |  | 5 |
| FCGR2A |  | 5 |
| FYB |  | 5 |
| GKN1 |  | 5 |
| GUCA1C |  | 5 |
| IFNA17 |  | 5 |
| JTB |  | 5 |
| KIAA0907 |  | 5 |
| KLK12 |  | 5 |
| MAGEB10 |  | 5 |
| MLLT1 |  | 5 |
| NMRK1 |  | 5 |
| PCCB |  | 5 |
| PHYH |  | 5 |
| PLXNA1 |  | 5 |
| PPP1R13L |  | 5 |
| PRC1 |  | 5 |
| PRUNE |  | 5 |
| RBBP7 |  | 5 |
| RCAN2 |  | 5 |
| REPS1 |  | 5 |
| SEMA4F |  | 5 |
| SLC6A2 |  | 5 |
| SNRNP27 |  | 5 |
| TIMM23 |  | 5 |
| WDTC1 |  | 5 |
| ACSF2 |  | 7 |
| ACVR2B |  | 7 |
| AKAP1 |  | 7 |
| ARPC5 |  | 7 |
| C14orf79 |  | 7 |
| C1QTNF3 |  | 7 |
| CEACAM1 |  | 7 |
| CNBD1 |  | 7 |
| CYLC2 |  | 7 |
| DEFB124 |  | 7 |
| DEFB135 |  | 7 |
| DSTN |  | 7 |
| DTX3L |  | 7 |
| EHD2 |  | 7 |
| ELANE |  | 7 |
| FAS |  | 7 |
| FBXO11 |  | 7 |
| FOS |  | 7 |
| GPAA1 |  | 7 |
| METTL19 |  | 7 |
| NAE1 |  | 7 |
| NUPL2 |  | 7 |
| OR1E1 |  | 7 |
| RAB15 |  | 7 |
| REG4 |  | 7 |
| SLC16A10 |  | 7 |
| SYTL1 |  | 7 |
| TNP1 |  | 7 |
| TYW5 |  | 7 |
| XPR1 |  | 7 |
| ZBTB37 |  | 7 |
| ZNF662 |  | 7 |
| ASF1B |  | 8 |
| C9orf173 |  | 8 |
| CACNG7 |  | 8 |
| CYC1 |  | 8 |
| DCP1A |  | 8 |
| DUSP7 |  | 8 |
| ECE2 |  | 8 |
| EIF2C2 |  | 8 |
| FAM127A |  | 8 |
| FAM54A |  | 8 |
| FMNL2 |  | 8 |
| GJB4 |  | 8 |
| HLA-E |  | 8 |
| IGFL3 |  | 8 |
| LEMD1 |  | 8 |
| LGALS9C |  | 8 |
| LIFR |  | 8 |
| MKNK1 |  | 8 |
| MRPS17 |  | 8 |
| NDUFB7 |  | 8 |
| PES1 |  | 8 |
| PSMD7 |  | 8 |
| RAB33A |  | 8 |
| RWDD2B |  | 8 |
| SCG3 |  | 8 |
| SETD3 |  | 8 |
| SLA |  | 8 |
| SOCS3 |  | 8 |
| ST6GALNAC4 |  | 8 |
| SUSD3 |  | 8 |
| TIMM17B |  | 8 |
| TMEM18 |  | 8 |
| TMEM216 |  | 8 |
| TOR1AIP1 |  | 8 |
| ZFHX3 |  | 8 |
| ZNF436 |  | 8 |
| ABCF2 |  | 9 |
| C11orf41 |  | 9 |
| C1D |  | 9 |
| CCL13 |  | 9 |
| CERS2 |  | 9 |
| DCST1 |  | 9 |
| DLGAP5 |  | 9 |
| DRD1 |  | 9 |
| DYRK3 |  | 9 |
| E2F3 |  | 9 |
| ETNK2 |  | 9 |
| GGPS1 |  | 9 |
| GP6 |  | 9 |
| GPR6 |  | 9 |
| HIST1H1T |  | 9 |
| KCNK7 |  | 9 |
| LDB3 |  | 9 |
| LRFN4 |  | 9 |
| NONO |  | 9 |
| OR5K1 |  | 9 |
| OTOA |  | 9 |
| PCP4 |  | 9 |
| PHACTR3 |  | 9 |
| PSME1 |  | 9 |
| RPH3A |  | 9 |
| SAMSN1 |  | 9 |
| SCNM1 |  | 9 |
| SFPQ |  | 9 |
| SLC45A2 |  | 9 |
| SSC5D |  | 9 |
| SSR4 |  | 9 |
| TMEM37 |  | 9 |
| TTC39B |  | 9 |
| ZNF764 |  | 9 |
| AP4S1 |  | 11 |
| ASIC3 |  | 11 |
| C12orf53 |  | 11 |
| C4orf37 |  | 11 |
| C8orf22 |  | 11 |
| CCKBR |  | 11 |
| CD68 |  | 11 |
| EXOSC3 |  | 11 |
| FAM110B |  | 11 |
| FTL |  | 11 |
| GNRH1 |  | 11 |
| HSPA13 |  | 11 |
| KIRREL2 |  | 11 |
| MED28 |  | 11 |
| MYL10 |  | 11 |
| NCSTN |  | 11 |
| PCDHGB3 |  | 11 |
| PEA15 |  | 11 |
| PLA2G5 |  | 11 |
| PRPF19 |  | 11 |
| PRRT3 |  | 11 |
| RRM1 |  | 11 |
| SELE |  | 11 |
| SHC2 |  | 11 |
| SLC25A47 |  | 11 |
| SYT12 |  | 11 |
| UPP1 |  | 11 |
| ZBBX |  | 11 |
| ZNF44 |  | 11 |
| C12orf39 |  | 12 |
| C19orf26 |  | 12 |
| C20orf24 |  | 12 |
| CCL18 |  | 12 |
| CYP19A1 |  | 12 |
| DDX53 |  | 12 |
| DNAJB9 |  | 12 |
| FLYWCH2 |  | 12 |
| FSD1L |  | 12 |
| G6PC3 |  | 12 |
| HS3ST5 |  | 12 |
| KTI12 |  | 12 |
| LYZL6 |  | 12 |
| NCF2 |  | 12 |
| PIGO |  | 12 |
| PPCS |  | 12 |
| RET |  | 12 |
| RNF7 |  | 12 |
| SIM2 |  | 12 |
| SLC9A3R2 |  | 12 |
| SYT5 |  | 12 |
| TMEM30A |  | 12 |
| WTAP |  | 12 |
| ZNF619 |  | 12 |
| CASC4 |  | 13 |
| CCDC56 |  | 13 |
| CD7 |  | 13 |
| CSAG2 |  | 13 |
| GOLT1A |  | 13 |
| HSDL2 |  | 13 |
| IDNK |  | 13 |
| MCM2 |  | 13 |
| NCR3 |  | 13 |
| OR7A5 |  | 13 |
| SETBP1 |  | 13 |
| SLAMF9 |  | 13 |
| STK38 |  | 13 |
| TMEM213 |  | 13 |
| TMEM51 |  | 13 |
| USP6 |  | 13 |
| XAF1 |  | 13 |
| ZFP30 |  | 13 |
| ARMS2 |  | 15 |
| ASIC4 |  | 15 |
| ATXN7L3 |  | 15 |
| C2orf84 |  | 15 |
| CPSF7 |  | 15 |
| GPIHBP1 |  | 15 |
| IL17B |  | 15 |
| MAGEB3 |  | 15 |
| PDE6B |  | 15 |
| RPL36A |  | 15 |
| RPL6 |  | 15 |
| SLC22A5 |  | 15 |
| SP2 |  | 15 |
| STX5 |  | 15 |
| TBC1D22A |  | 15 |
| TWF2 |  | 15 |
| VTN |  | 15 |
| ZNF582 |  | 15 |
| ATF7IP2 |  | 16 |
| FAM49B |  | 16 |
| FECH |  | 16 |
| HOXB8 |  | 16 |
| LRRTM4 |  | 16 |
| ODF3L1 |  | 16 |
| PER1 |  | 16 |
| SLX1A |  | 16 |
| SSX3 |  | 16 |
| ARFGAP3 |  | 17 |
| C18orf1 |  | 17 |
| DYNLL1 |  | 17 |
| MYO1F |  | 17 |
| POLD4 |  | 17 |
| RRP36 |  | 17 |
| TMED6 |  | 17 |
| ZNF674 |  | 17 |
| YPEL2 |  | 19 |
| ALKBH3 |  | 19 |
| ANKRD36 |  | 19 |
| BVES |  | 19 |
| CST1 |  | 19 |
| DPM2 |  | 19 |
| HSD11B1L |  | 19 |
| RGS8 |  | 19 |
| TUB |  | 19 |
| TXNDC16 |  | 19 |
| ACADSB |  | 20 |
| ADSS |  | 20 |
| ASCL4 |  | 20 |
| CYP20A1 |  | 20 |
| FAM176C |  | 20 |
| HYAL2 |  | 20 |
| PIF1 |  | 20 |
| PIK3IP1 |  | 20 |
| PIP5K1B |  | 20 |
| TAS2R40 |  | 20 |
| C19orf76 |  | 21 |
| CT45A4 |  | 21 |
| DNAJC1 |  | 21 |
| LRRC29 |  | 21 |
| ODF3 |  | 21 |
| SEC63 |  | 21 |
| STK17A |  | 21 |
| TMEM211 |  | 21 |
| C9orf80 |  | 23 |
| FAM198B |  | 23 |
| FNDC3B |  | 23 |
| KANSL1L |  | 23 |
| ULBP1 |  | 23 |
| VCAM1 |  | 23 |
| PKP3 |  | 24 |
| POTEE |  | 24 |
| ZNF785 |  | 24 |
| ACAT1 |  | 25 |
| CER1 |  | 25 |
| ID2 |  | 25 |
| LHX9 |  | 25 |
| TBCC |  | 25 |
| ZNF493 |  | 25 |
| C8orf58 |  | 27 |
| LAD1 |  | 27 |
| TM2D3 |  | 27 |
| TMA16 |  | 27 |
| U2AF1 |  | 27 |
| DYNC2LI1 |  | 28 |
| DDX39A |  | 31 |
| NR1H4 |  | 32 |
| RARG |  | 32 |
| CRB3 |  | 33 |
| LIME1 |  | 33 |
| HIST1H2BE |  | 36 |
| NR2C2 |  | 36 |
| WDR74 |  | 37 |
| 41153 |  | 40 |
| FAM129C |  | 40 |
| ZBTB38 |  | 40 |
| KLC2 |  | 41 |
| IRS1 |  | 43 |
| KAZN |  | 44 |
| LMO1 |  | 52 |
| ZSCAN21 |  | 52 |
| HOXD4 |  | 55 |
| HOXC10 |  | 61 |
| EZR |  | 80 |
| ADH1C |  | 1 |
| AIM1L |  | 1 |
| B3GALT6 |  | 1 |
| C14orf129 |  | 1 |
| C1orf124 |  | 1 |
| CD27 |  | 1 |
| CD44 |  | 1 |
| CDC25C |  | 1 |
| CPNE2 |  | 1 |
| CRISP1 |  | 1 |
| DBH |  | 1 |
| DEFB130 |  | 1 |
| DPP7 |  | 1 |
| DUSP3 |  | 1 |
| EMR3 |  | 1 |
| FAM105A |  | 1 |
| FAM169B |  | 1 |
| FAM19A3 |  | 1 |
| FAM5B |  | 1 |
| FANCM |  | 1 |
| GALM |  | 1 |
| GPATCH2 |  | 1 |
| GPR52 |  | 1 |
| GRIPAP1 |  | 1 |
| HDHD3 |  | 1 |
| INHA |  | 1 |
| IP6K3 |  | 1 |
| ITIH3 |  | 1 |
| ITK |  | 1 |
| KCNAB3 |  | 1 |
| KCNE3 |  | 1 |
| MAPK12 |  | 1 |
| MST1 |  | 1 |
| NACC2 |  | 1 |
| NDUFS7 |  | 1 |
| NID2 |  | 1 |
| NPTX1 |  | 1 |
| NR1I2 |  | 1 |
| OR8B4 |  | 1 |
| PAQR7 |  | 1 |
| PDRG1 |  | 1 |
| PIK3R3 |  | 1 |
| PLGLB1 |  | 1 |
| PRKAR2A |  | 1 |
| RAB37 |  | 1 |
| RAPSN |  | 1 |
| RASSF7 |  | 1 |
| RDH16 |  | 1 |
| RECK |  | 1 |
| RNF141 |  | 1 |
| SENP5 |  | 1 |
| SENP8 |  | 1 |
| SH3BGRL3 |  | 1 |
| SHISA6 |  | 1 |
| SHISA7 |  | 1 |
| SMO |  | 1 |
| SPAG11B |  | 1 |
| TACR3 |  | 1 |
| TBL1Y |  | 1 |
| TMC6 |  | 1 |
| TMEM101 |  | 1 |
| TMEM66 |  | 1 |
| TPP1 |  | 1 |
| TRABD |  | 1 |
| UBE2O |  | 1 |
| UBQLN4 |  | 1 |
| VPS41 |  | 1 |
| WDR85 |  | 1 |
| ZFP2 |  | 1 |
| ZNF117 |  | 1 |
| ZNF626 |  | 1 |
| AASDHPPT |  | 3 |
| ACYP1 |  | 3 |
| ADCY5 |  | 3 |
| ADH6 |  | 3 |
| AHI1 |  | 3 |
| ALDH3B1 |  | 3 |
| ANKRD16 |  | 3 |
| APOBEC3D |  | 3 |
| ASPM |  | 3 |
| ATAT1 |  | 3 |
| BDKRB1 |  | 3 |
| BEND4 |  | 3 |
| BET1 |  | 3 |
| C10orf118 |  | 3 |
| C1orf162 |  | 3 |
| C3orf52 |  | 3 |
| CD38 |  | 3 |
| CD96 |  | 3 |
| CFC1 |  | 3 |
| CHST4 |  | 3 |
| CMPK1 |  | 3 |
| CNPY2 |  | 3 |
| CNRIP1 |  | 3 |
| CPNE9 |  | 3 |
| DAO |  | 3 |
| DDX17 |  | 3 |
| DEFB118 |  | 3 |
| DHX8 |  | 3 |
| DTNA |  | 3 |
| EDEM2 |  | 3 |
| EEF1D |  | 3 |
| EFNA1 |  | 3 |
| EIF4ENIF1 |  | 3 |
| ENAH |  | 3 |
| EPB42 |  | 3 |
| EPHB3 |  | 3 |
| FABP2 |  | 3 |
| FAM196A |  | 3 |
| FAM32A |  | 3 |
| FAM76B |  | 3 |
| FBXW10 |  | 3 |
| G3BP2 |  | 3 |
| GABPA |  | 3 |
| GABRR1 |  | 3 |
| GAD1 |  | 3 |
| GADD45A |  | 3 |
| GIT2 |  | 3 |
| GLT1D1 |  | 3 |
| GPRC5B |  | 3 |
| GSX1 |  | 3 |
| H2AFJ |  | 3 |
| H2AFZ |  | 3 |
| HCFC1R1 |  | 3 |
| HEMK1 |  | 3 |
| HIST2H4A |  | 3 |
| HPD |  | 3 |
| IFRD2 |  | 3 |
| IL17RB |  | 3 |
| KCTD6 |  | 3 |
| KLK9 |  | 3 |
| LGALS7B |  | 3 |
| LIPT1 |  | 3 |
| LSM14B |  | 3 |
| MAP1LC3B |  | 3 |
| MFAP3L |  | 3 |
| MRPL1 |  | 3 |
| MRPS16 |  | 3 |
| MRPS35 |  | 3 |
| MTM1 |  | 3 |
| NAP1L5 |  | 3 |
| NAT8B |  | 3 |
| NEUROD2 |  | 3 |
| NME5 |  | 3 |
| NPAS1 |  | 3 |
| NPR3 |  | 3 |
| OR4F3 |  | 3 |
| OR8U8 |  | 3 |
| OSTC |  | 3 |
| OTOP3 |  | 3 |
| PAH |  | 3 |
| PFDN2 |  | 3 |
| PFN1 |  | 3 |
| PGPEP1 |  | 3 |
| PHOSPHO2 |  | 3 |
| PIP4K2A |  | 3 |
| POLK |  | 3 |
| POMZP3 |  | 3 |
| PPP2R2D |  | 3 |
| PTCRA |  | 3 |
| PTGDS |  | 3 |
| RELL2 |  | 3 |
| RHOD |  | 3 |
| RPS2 |  | 3 |
| SCNN1D |  | 3 |
| SECTM1 |  | 3 |
| SEL1L3 |  | 3 |
| SIGLEC10 |  | 3 |
| SLC27A5 |  | 3 |
| SNTG1 |  | 3 |
| SNX7 |  | 3 |
| SRSF2 |  | 3 |
| SSR2 |  | 3 |
| SSRP1 |  | 3 |
| SYAP1 |  | 3 |
| SYBU |  | 3 |
| TCEB3C |  | 3 |
| TGFBR2 |  | 3 |
| TM6SF2 |  | 3 |
| TMCO2 |  | 3 |
| TMEM70 |  | 3 |
| TPD52L1 |  | 3 |
| TRAK2 |  | 3 |
| TRPM1 |  | 3 |
| TTL |  | 3 |
| TUBB2B |  | 3 |
| USP31 |  | 3 |
| WAC |  | 3 |
| WNT7A |  | 3 |
| WTIP |  | 3 |
| XKR8 |  | 3 |
| ZBTB49 |  | 3 |
| ZHX1-C8ORF76 |  | 3 |
| ZNF559 |  | 3 |
| ZNF773 |  | 3 |
| ACBD7 |  | 4 |
| AFMID |  | 4 |
| AKR1A1 |  | 4 |
| AKR1B10 |  | 4 |
| ALDH2 |  | 4 |
| ALPI |  | 4 |
| ARL9 |  | 4 |
| ARSF |  | 4 |
| ASPDH |  | 4 |
| B3GALT5 |  | 4 |
| C15orf41 |  | 4 |
| C1orf116 |  | 4 |
| C20orf20 |  | 4 |
| C5orf38 |  | 4 |
| C9orf86 |  | 4 |
| CADM1 |  | 4 |
| CBR3 |  | 4 |
| CBWD2 |  | 4 |
| CDC42SE2 |  | 4 |
| CDO1 |  | 4 |
| CHMP2B |  | 4 |
| CORO7 |  | 4 |
| CPVL |  | 4 |
| CST11 |  | 4 |
| CYBRD1 |  | 4 |
| CYP27C1 |  | 4 |
| CYTH4 |  | 4 |
| DBX1 |  | 4 |
| DCT |  | 4 |
| DCTN6 |  | 4 |
| DEFB4A |  | 4 |
| EGLN1 |  | 4 |
| EHD1 |  | 4 |
| EPHA1 |  | 4 |
| EPS8L2 |  | 4 |
| EREG |  | 4 |
| EXD1 |  | 4 |
| FARSA |  | 4 |
| FASTK |  | 4 |
| FCGR1A |  | 4 |
| FCN3 |  | 4 |
| FGD6 |  | 4 |
| GABRB2 |  | 4 |
| GBP7 |  | 4 |
| GCKR |  | 4 |
| GNAO1 |  | 4 |
| GPR157 |  | 4 |
| GPR35 |  | 4 |
| GTF2H4 |  | 4 |
| HEY1 |  | 4 |
| HIBCH |  | 4 |
| HIST1H2AG |  | 4 |
| HYI |  | 4 |
| IGSF1 |  | 4 |
| IL10RA |  | 4 |
| KERA |  | 4 |
| KIR2DS3 |  | 4 |
| KRTAP22-1 |  | 4 |
| LIPC |  | 4 |
| MBD3L2 |  | 4 |
| MED20 |  | 4 |
| MFNG |  | 4 |
| MIOS |  | 4 |
| MOCS1 |  | 4 |
| MRPL33 |  | 4 |
| MSTO1 |  | 4 |
| NEFL |  | 4 |
| NFAM1 |  | 4 |
| NOXRED1 |  | 4 |
| NQO1 |  | 4 |
| NTSR1 |  | 4 |
| NUDT13 |  | 4 |
| PADI3 |  | 4 |
| PDE1C |  | 4 |
| PDK4 |  | 4 |
| PELI3 |  | 4 |
| PEX7 |  | 4 |
| PHGDH |  | 4 |
| PHYHD1 |  | 4 |
| POLE |  | 4 |
| PPIA |  | 4 |
| PPIAL4A |  | 4 |
| PPM1J |  | 4 |
| PPP1CA |  | 4 |
| PPY |  | 4 |
| PRDX6 |  | 4 |
| PSORS1C2 |  | 4 |
| PVRL3 |  | 4 |
| RAB39B |  | 4 |
| RBM15B |  | 4 |
| RHOBTB1 |  | 4 |
| RNF13 |  | 4 |
| RPIA |  | 4 |
| RPL26 |  | 4 |
| RUFY2 |  | 4 |
| SERINC1 |  | 4 |
| SGSH |  | 4 |
| SIAE |  | 4 |
| SIAH1 |  | 4 |
| SIX2 |  | 4 |
| SLC10A7 |  | 4 |
| SMAD7 |  | 4 |
| SNRPD3 |  | 4 |
| SNX27 |  | 4 |
| SOCS2 |  | 4 |
| SPRYD7 |  | 4 |
| SRSF8 |  | 4 |
| STT3B |  | 4 |
| TBRG4 |  | 4 |
| TMEM107 |  | 4 |
| TMEM136 |  | 4 |
| TMEM167B |  | 4 |
| TNFSF13 |  | 4 |
| TNNT1 |  | 4 |
| TUSC2 |  | 4 |
| VSIG1 |  | 4 |
| WDR37 |  | 4 |
| ACTRT2 |  | 5 |
| AHNAK |  | 5 |
| ANKRD1 |  | 5 |
| ARPC1B |  | 5 |
| ASCC2 |  | 5 |
| ATP5B |  | 5 |
| B3GAT2 |  | 5 |
| BPGM |  | 5 |
| C15orf53 |  | 5 |
| C7orf34 |  | 5 |
| CCDC59 |  | 5 |
| CCL17 |  | 5 |
| CDH7 |  | 5 |
| CLDN14 |  | 5 |
| COPS5 |  | 5 |
| COQ10B |  | 5 |
| COQ4 |  | 5 |
| CPSF4 |  | 5 |
| CSH1 |  | 5 |
| CXCL3 |  | 5 |
| DCX |  | 5 |
| DEFB106A |  | 5 |
| EGLN3 |  | 5 |
| EMC7 |  | 5 |
| EXOC8 |  | 5 |
| FAM149B1 |  | 5 |
| FBXL19 |  | 5 |
| FGD2 |  | 5 |
| FGF14 |  | 5 |
| FGGY |  | 5 |
| FOXD4 |  | 5 |
| GNAT2 |  | 5 |
| GPR128 |  | 5 |
| GPRC5C |  | 5 |
| GTF3C3 |  | 5 |
| HACL1 |  | 5 |
| HDDC2 |  | 5 |
| HIST1H4G |  | 5 |
| IFT80 |  | 5 |
| ISOC2 |  | 5 |
| KIF1B |  | 5 |
| KIR2DL5A |  | 5 |
| KLHDC8A |  | 5 |
| LILRA2 |  | 5 |
| LIN7B |  | 5 |
| LOXL2 |  | 5 |
| LRRC48 |  | 5 |
| MAU2 |  | 5 |
| MCFD2 |  | 5 |
| MCOLN3 |  | 5 |
| METTL20 |  | 5 |
| MTMR10 |  | 5 |
| MTRF1L |  | 5 |
| NIPSNAP3B |  | 5 |
| NPM3 |  | 5 |
| NSFL1C |  | 5 |
| OR52W1 |  | 5 |
| PARP16 |  | 5 |
| PDHB |  | 5 |
| PGK2 |  | 5 |
| PHPT1 |  | 5 |
| PLB1 |  | 5 |
| PNPLA6 |  | 5 |
| PRSS38 |  | 5 |
| PSMG3 |  | 5 |
| PTCH2 |  | 5 |
| RARRES1 |  | 5 |
| RBBP6 |  | 5 |
| RERG |  | 5 |
| RFC5 |  | 5 |
| RGR |  | 5 |
| RNF150 |  | 5 |
| S100A8 |  | 5 |
| S1PR2 |  | 5 |
| SAMHD1 |  | 5 |
| SAPCD1 |  | 5 |
| SLC4A8 |  | 5 |
| SLC50A1 |  | 5 |
| SLC7A5 |  | 5 |
| SPATA6L |  | 5 |
| SRP14 |  | 5 |
| STK17B |  | 5 |
| TCEAL1 |  | 5 |
| TEKT5 |  | 5 |
| TMED2 |  | 5 |
| TPTE2 |  | 5 |
| TRIM11 |  | 5 |
| TRIT1 |  | 5 |
| TRO |  | 5 |
| UCHL5 |  | 5 |
| UNC45B |  | 5 |
| USP1 |  | 5 |
| WBSCR16 |  | 5 |
| WDR33 |  | 5 |
| YARS2 |  | 5 |
| ZNF585B |  | 5 |
| ZNF79 |  | 5 |
| ALG5 |  | 7 |
| ALPK1 |  | 7 |
| ARF6 |  | 7 |
| BCKDHB |  | 7 |
| C14orf159 |  | 7 |
| CCDC82 |  | 7 |
| CD300LG |  | 7 |
| CDH19 |  | 7 |
| CTSL2 |  | 7 |
| DDX49 |  | 7 |
| DEFA1B |  | 7 |
| DENND5A |  | 7 |
| EIF3L |  | 7 |
| EVI2A |  | 7 |
| FAM167B |  | 7 |
| FBXO30 |  | 7 |
| GLUL |  | 7 |
| HELT |  | 7 |
| HSPA4 |  | 7 |
| IGSF6 |  | 7 |
| IL1RL1 |  | 7 |
| INTS10 |  | 7 |
| LEPREL2 |  | 7 |
| LRRC6 |  | 7 |
| LYPLAL1 |  | 7 |
| MAT2B |  | 7 |
| MGARP |  | 7 |
| NBPF1 |  | 7 |
| OAS1 |  | 7 |
| OBP2A |  | 7 |
| PATE1 |  | 7 |
| PCDHGA5 |  | 7 |
| PLCZ1 |  | 7 |
| POC1B |  | 7 |
| PROZ |  | 7 |
| RHBDF1 |  | 7 |
| RNF121 |  | 7 |
| RPL9 |  | 7 |
| RRN3 |  | 7 |
| S100A11 |  | 7 |
| SAFB2 |  | 7 |
| SDS |  | 7 |
| SEMA3B |  | 7 |
| SERPINA12 |  | 7 |
| SGCA |  | 7 |
| SLC25A15 |  | 7 |
| SLC4A5 |  | 7 |
| SLC8A2 |  | 7 |
| SMARCD1 |  | 7 |
| SPINK14 |  | 7 |
| STARD6 |  | 7 |
| TJP2 |  | 7 |
| TMEM108 |  | 7 |
| TSR1 |  | 7 |
| UBL5 |  | 7 |
| UGT1A3 |  | 7 |
| ZNF586 |  | 7 |
| ZNF747 |  | 7 |
| ZXDC |  | 7 |
| AARS |  | 8 |
| ADAP2 |  | 8 |
| AEN |  | 8 |
| ANAPC10 |  | 8 |
| APOL4 |  | 8 |
| BMP6 |  | 8 |
| C10orf122 |  | 8 |
| C14orf135 |  | 8 |
| C1orf198 |  | 8 |
| C20orf203 |  | 8 |
| C3orf19 |  | 8 |
| CCDC116 |  | 8 |
| CD48 |  | 8 |
| CGB5 |  | 8 |
| CHST6 |  | 8 |
| CXCL11 |  | 8 |
| CYP8B1 |  | 8 |
| DALRD3 |  | 8 |
| EPHA8 |  | 8 |
| ERCC5 |  | 8 |
| FCRL1 |  | 8 |
| FN3KRP |  | 8 |
| FNDC4 |  | 8 |
| FOXD4L3 |  | 8 |
| HIST1H3E |  | 8 |
| IKBKE |  | 8 |
| LACTB2 |  | 8 |
| MBTPS2 |  | 8 |
| MMP12 |  | 8 |
| MRPL46 |  | 8 |
| NPM2 |  | 8 |
| NUDT16 |  | 8 |
| OGN |  | 8 |
| OR2H1 |  | 8 |
| PLAUR |  | 8 |
| PNCK |  | 8 |
| PRR15 |  | 8 |
| PRR16 |  | 8 |
| RAB24 |  | 8 |
| RASL11B |  | 8 |
| RBM3 |  | 8 |
| RFK |  | 8 |
| ROM1 |  | 8 |
| SLC48A1 |  | 8 |
| SNCAIP |  | 8 |
| SNTA1 |  | 8 |
| SPINLW1 |  | 8 |
| SPOCK3 |  | 8 |
| SUN1 |  | 8 |
| TEX33 |  | 8 |
| TMEM161B |  | 8 |
| TMEM169 |  | 8 |
| TMEM220 |  | 8 |
| TMPO |  | 8 |
| TMPRSS2 |  | 8 |
| TMPRSS7 |  | 8 |
| TNFSF8 |  | 8 |
| TYROBP |  | 8 |
| ULK3 |  | 8 |
| WDR7 |  | 8 |
| YPEL1 |  | 8 |
| ZMYM6NB |  | 8 |
| ACER1 |  | 9 |
| ACOX2 |  | 9 |
| AFP |  | 9 |
| BRDT |  | 9 |
| C12orf43 |  | 9 |
| C17orf109 |  | 9 |
| C22orf32 |  | 9 |
| C4orf47 |  | 9 |
| C6orf118 |  | 9 |
| EID3 |  | 9 |
| EMID1 |  | 9 |
| EMR1 |  | 9 |
| ETFB |  | 9 |
| FAM217A |  | 9 |
| FUT8 |  | 9 |
| GAS2L1 |  | 9 |
| GRK6 |  | 9 |
| HIST2H2AB |  | 9 |
| HTR3D |  | 9 |
| IGF2BP3 |  | 9 |
| KLRC4 |  | 9 |
| MAPKAPK2 |  | 9 |
| MED19 |  | 9 |
| MMP9 |  | 9 |
| NOP56 |  | 9 |
| NUMBL |  | 9 |
| OR2K2 |  | 9 |
| OR8D1 |  | 9 |
| RABL3 |  | 9 |
| RIC8A |  | 9 |
| S100A3 |  | 9 |
| SOX15 |  | 9 |
| STMN2 |  | 9 |
| TCIRG1 |  | 9 |
| TPTE |  | 9 |
| TRIM65 |  | 9 |
| TTC25 |  | 9 |
| TTYH3 |  | 9 |
| WFDC2 |  | 9 |
| ZNF140 |  | 9 |
| ZNF623 |  | 9 |
| AGTR1 |  | 11 |
| BMPR1B |  | 11 |
| C22orf31 |  | 11 |
| CCDC9 |  | 11 |
| CDC34 |  | 11 |
| CHRNA7 |  | 11 |
| DTWD2 |  | 11 |
| EDDM3B |  | 11 |
| EFNA2 |  | 11 |
| EVX1 |  | 11 |
| FAM209B |  | 11 |
| FGF12 |  | 11 |
| HIPK2 |  | 11 |
| HIST1H2BK |  | 11 |
| HLA-DPB1 |  | 11 |
| IAPP |  | 11 |
| LARP1B |  | 11 |
| LILRB4 |  | 11 |
| LY9 |  | 11 |
| LYSMD1 |  | 11 |
| MC3R |  | 11 |
| MEAF6 |  | 11 |
| NF2 |  | 11 |
| OR8D2 |  | 11 |
| PCYT2 |  | 11 |
| PF4V1 |  | 11 |
| POLR3G |  | 11 |
| PPME1 |  | 11 |
| PTH |  | 11 |
| S100A9 |  | 11 |
| SLC25A10 |  | 11 |
| SULT1A1 |  | 11 |
| THY1 |  | 11 |
| UNKL |  | 11 |
| UTP14A |  | 11 |
| YPEL4 |  | 11 |
| ZNF383 |  | 11 |
| ZNF480 |  | 11 |
| C3orf43 |  | 12 |
| C9orf152 |  | 12 |
| CBR4 |  | 12 |
| CCL1 |  | 12 |
| CENPA |  | 12 |
| COG2 |  | 12 |
| CYFIP1 |  | 12 |
| DCTN3 |  | 12 |
| EDNRB |  | 12 |
| FBXO16 |  | 12 |
| GRID1 |  | 12 |
| GTPBP10 |  | 12 |
| HCST |  | 12 |
| HGFAC |  | 12 |
| HSF2 |  | 12 |
| KDELR1 |  | 12 |
| L2HGDH |  | 12 |
| MAPKAPK5 |  | 12 |
| MCOLN2 |  | 12 |
| MDK |  | 12 |
| MTRF1 |  | 12 |
| NOB1 |  | 12 |
| NR2C1 |  | 12 |
| OLFML2B |  | 12 |
| P2RX1 |  | 12 |
| PSENEN |  | 12 |
| RPS6KA1 |  | 12 |
| S100A13 |  | 12 |
| SLC34A1 |  | 12 |
| SLC5A12 |  | 12 |
| SYNC |  | 12 |
| TAAR2 |  | 12 |
| TMEM116 |  | 12 |
| TWIST2 |  | 12 |
| VN1R2 |  | 12 |
| ZNF322 |  | 12 |
| ZNF461 |  | 12 |
| ZNF92 |  | 12 |
| ABRA |  | 13 |
| ADTRP |  | 13 |
| ALDH6A1 |  | 13 |
| C10orf125 |  | 13 |
| C17orf85 |  | 13 |
| C20orf79 |  | 13 |
| C21orf59 |  | 13 |
| C2orf73 |  | 13 |
| CD74 |  | 13 |
| CELA1 |  | 13 |
| CLDN17 |  | 13 |
| CLDN18 |  | 13 |
| CRNN |  | 13 |
| CSPP1 |  | 13 |
| CYP2B6 |  | 13 |
| DOK1 |  | 13 |
| ECM2 |  | 13 |
| FADS3 |  | 13 |
| GJD4 |  | 13 |
| GNB1 |  | 13 |
| GUCY1A3 |  | 13 |
| HLA-DRB4 |  | 13 |
| HTR5A |  | 13 |
| IKBIP |  | 13 |
| IL23R |  | 13 |
| NXN |  | 13 |
| OR10G3 |  | 13 |
| PCBP4 |  | 13 |
| PCDHGB6 |  | 13 |
| PDILT |  | 13 |
| PHYHIP |  | 13 |
| PIGK |  | 13 |
| RNF180 |  | 13 |
| SAP30BP |  | 13 |
| SETX |  | 13 |
| SIGMAR1 |  | 13 |
| SLC20A1 |  | 13 |
| TMEM173 |  | 13 |
| TMEM184A |  | 13 |
| ZNF25 |  | 13 |
| C19orf38 |  | 15 |
| 40787 |  | 15 |
| BOLA2B |  | 15 |
| CAB39L |  | 15 |
| COLEC11 |  | 15 |
| DMKN |  | 15 |
| GPC5 |  | 15 |
| MLST8 |  | 15 |
| PHACTR4 |  | 15 |
| PNLIP |  | 15 |
| PPP2R2B |  | 15 |
| PQBP1 |  | 15 |
| RND1 |  | 15 |
| TDGF1 |  | 15 |
| TMEM8B |  | 15 |
| TRPC7 |  | 15 |
| USP47 |  | 15 |
| ZNF8 |  | 15 |
| ZNHIT6 |  | 15 |
| ATXN7L1 |  | 16 |
| C2orf61 |  | 16 |
| CCDC106 |  | 16 |
| CEP57 |  | 16 |
| EFNB1 |  | 16 |
| GET4 |  | 16 |
| GHSR |  | 16 |
| LRRC55 |  | 16 |
| PRSS23 |  | 16 |
| RASGRP4 |  | 16 |
| SLC25A12 |  | 16 |
| SORCS2 |  | 16 |
| SPATA4 |  | 16 |
| SPTLC1 |  | 16 |
| STAC2 |  | 16 |
| TBX22 |  | 16 |
| TMEM68 |  | 16 |
| TPPP3 |  | 16 |
| ZNF577 |  | 16 |
| ALDH3A1 |  | 17 |
| ANKRD20A1 |  | 17 |
| CCL7 |  | 17 |
| CCR3 |  | 17 |
| CLASP2 |  | 17 |
| FAM65C |  | 17 |
| GABRA3 |  | 17 |
| GGN |  | 17 |
| LECT2 |  | 17 |
| LRRC40 |  | 17 |
| NOG |  | 17 |
| P2RX2 |  | 17 |
| PRR15L |  | 17 |
| PTRH1 |  | 17 |
| PTTG2 |  | 17 |
| RPS27 |  | 17 |
| SETD5 |  | 17 |
| C21orf58 |  | 19 |
| C6orf15 |  | 19 |
| CLEC4M |  | 19 |
| ESAM |  | 19 |
| GALNTL5 |  | 19 |
| GPR115 |  | 19 |
| KIAA0825 |  | 19 |
| LAIR1 |  | 19 |
| LANCL1 |  | 19 |
| MB21D2 |  | 19 |
| MORF4L1 |  | 19 |
| MSL1 |  | 19 |
| SARS |  | 19 |
| SNRNP35 |  | 19 |
| USP53 |  | 19 |
| ZFP36L1 |  | 19 |
| ZNF254 |  | 19 |
| ARFGAP2 |  | 20 |
| C2orf65 |  | 20 |
| C9orf9 |  | 20 |
| CDH5 |  | 20 |
| MUM1L1 |  | 20 |
| PAPD7 |  | 20 |
| SAP130 |  | 20 |
| SKA2 |  | 20 |
| TEFM |  | 20 |
| BUB3 |  | 21 |
| CUL2 |  | 21 |
| DNAJC4 |  | 21 |
| DUSP26 |  | 21 |
| FAM216B |  | 21 |
| KCNK16 |  | 21 |
| LGMN |  | 21 |
| MED9 |  | 21 |
| NEUROG1 |  | 21 |
| NOP16 |  | 21 |
| NSL1 |  | 21 |
| OR1J4 |  | 21 |
| TNFRSF17 |  | 21 |
| WNT3 |  | 21 |
| ZC2HC1B |  | 21 |
| ZNF187 |  | 21 |
| ZNF415 |  | 21 |
| ANKHD1 |  | 23 |
| C1orf172 |  | 23 |
| FAM190A |  | 23 |
| GPR26 |  | 23 |
| PTPRB |  | 23 |
| RING1 |  | 23 |
| RPL34 |  | 23 |
| RXRG |  | 23 |
| TPPP |  | 23 |
| ZKSCAN1 |  | 23 |
| ARMC3 |  | 24 |
| C18orf21 |  | 24 |
| COX7A2 |  | 24 |
| HOXB3 |  | 24 |
| IFNAR2 |  | 24 |
| TMUB2 |  | 24 |
| ZNF621 |  | 24 |
| ERGIC2 |  | 25 |
| SALL2 |  | 25 |
| STK33 |  | 25 |
| CRISPLD1 |  | 27 |
| PDIA4 |  | 27 |
| WDR92 |  | 27 |
| ZBP1 |  | 27 |
| ZNF846 |  | 27 |
| GABPB2 |  | 28 |
| GGT1 |  | 28 |
| NUFIP2 |  | 28 |
| SC5DL |  | 28 |
| SLC27A2 |  | 28 |
| CYP2C18 |  | 29 |
| PF4 |  | 29 |
| ATP11B |  | 31 |
| SLITRK4 |  | 31 |
| YTHDF2 |  | 31 |
| C11orf87 |  | 32 |
| NANOG |  | 32 |
| SNAPC1 |  | 32 |
| TMEM183A |  | 32 |
| MYC |  | 35 |
| CHST10 |  | 36 |
| NKX2-3 |  | 36 |
| ZXDA |  | 36 |
| FAM76A |  | 37 |
| TPRN |  | 37 |
| C9orf139 |  | 39 |
| KLF15 |  | 39 |
| SLC39A1 |  | 39 |
| RTN1 |  | 40 |
| CNN3 |  | 41 |
| SYT6 |  | 44 |
| GIPC2 |  | 48 |
| HSFY1 |  | 49 |
| CDC42EP4 |  | 53 |
| KIAA0408 |  | 53 |
| CXXC1 |  | 55 |
| HOXB6 |  | 57 |
| ZNF341 |  | 57 |
| CCDC130 |  | 59 |
| HOXA1 |  | 77 |
| KPNA5 |  | 81 |
| RAX |  | 84 |
| 38231 |  | 1 |
| ABCG2 |  | 1 |
| ABHD8 |  | 1 |
| ACADM |  | 1 |
| ACADVL |  | 1 |
| ACOX3 |  | 1 |
| ACP6 |  | 1 |
| ACSBG2 |  | 1 |
| ACSM5 |  | 1 |
| ACTG1 |  | 1 |
| ACTR8 |  | 1 |
| ACVR1B |  | 1 |
| ACVR1C |  | 1 |
| ADAM21 |  | 1 |
| ADAM32 |  | 1 |
| ADAT3 |  | 1 |
| ADRBK2 |  | 1 |
| AGPAT1 |  | 1 |
| AK8 |  | 1 |
| AKAP7 |  | 1 |
| AKIRIN2 |  | 1 |
| AKR1C2 |  | 1 |
| ALDH1L2 |  | 1 |
| ALPP |  | 1 |
| ANGPT2 |  | 1 |
| ANKRD12 |  | 1 |
| ANKRD39 |  | 1 |
| ANKRD50 |  | 1 |
| APBB3 |  | 1 |
| APLF |  | 1 |
| APOL6 |  | 1 |
| AQP4 |  | 1 |
| AREGB |  | 1 |
| ARHGAP11B |  | 1 |
| ARHGAP35 |  | 1 |
| ARIH1 |  | 1 |
| ARL4C |  | 1 |
| ARMCX2 |  | 1 |
| ARMCX5 |  | 1 |
| ARRDC3 |  | 1 |
| ARSI |  | 1 |
| ASB10 |  | 1 |
| ASNS |  | 1 |
| ATG10 |  | 1 |
| ATG4C |  | 1 |
| ATG5 |  | 1 |
| ATP6V0A1 |  | 1 |
| ATP6V0A4 |  | 1 |
| ATPAF2 |  | 1 |
| ATXN7 |  | 1 |
| AURKAIP1 |  | 1 |
| AVPR1B |  | 1 |
| B3GALNT2 |  | 1 |
| B3GNT5 |  | 1 |
| BAZ2B |  | 1 |
| BBS5 |  | 1 |
| BCAP31 |  | 1 |
| BCAS4 |  | 1 |
| BMP5 |  | 1 |
| BMP7 |  | 1 |
| BPIFB3 |  | 1 |
| BPNT1 |  | 1 |
| BRIX1 |  | 1 |
| BTBD1 |  | 1 |
| BTBD17 |  | 1 |
| BTC |  | 1 |
| BTLA |  | 1 |
| BUB1B |  | 1 |
| C10orf27 |  | 1 |
| C10orf90 |  | 1 |
| C11orf82 |  | 1 |
| C15orf23 |  | 1 |
| C15orf29 |  | 1 |
| C17orf77 |  | 1 |
| C1orf105 |  | 1 |
| C1orf106 |  | 1 |
| C1orf111 |  | 1 |
| C1orf182 |  | 1 |
| C1orf201 |  | 1 |
| C1orf226 |  | 1 |
| C1orf27 |  | 1 |
| C1orf53 |  | 1 |
| C1QL3 |  | 1 |
| C1QTNF2 |  | 1 |
| C1R |  | 1 |
| C22orf43 |  | 1 |
| C2orf77 |  | 1 |
| C4BPA |  | 1 |
| C5orf4 |  | 1 |
| C5orf48 |  | 1 |
| C6orf195 |  | 1 |
| C6orf58 |  | 1 |
| C8orf40 |  | 1 |
| C9orf16 |  | 1 |
| CA12 |  | 1 |
| CACNG6 |  | 1 |
| CALCA |  | 1 |
| CAMK1D |  | 1 |
| CAMKK2 |  | 1 |
| CAPN11 |  | 1 |
| CASP9 |  | 1 |
| CASS4 |  | 1 |
| CAV2 |  | 1 |
| CBLC |  | 1 |
| CCDC89 |  | 1 |
| CCL28 |  | 1 |
| CCL3 |  | 1 |
| CCL4L2 |  | 1 |
| CCRN4L |  | 1 |
| CD1A |  | 1 |
| CD1E |  | 1 |
| CD226 |  | 1 |
| CD274 |  | 1 |
| CD93 |  | 1 |
| CDH26 |  | 1 |
| CDK15 |  | 1 |
| CDKL1 |  | 1 |
| CDKN2C |  | 1 |
| CDYL2 |  | 1 |
| CEBPE |  | 1 |
| CERS6 |  | 1 |
| CES3 |  | 1 |
| CES4A |  | 1 |
| CFB |  | 1 |
| CFHR4 |  | 1 |
| CGB |  | 1 |
| CGREF1 |  | 1 |
| CHCHD6 |  | 1 |
| CHIA |  | 1 |
| CHMP1A |  | 1 |
| CHRNB1 |  | 1 |
| CHRND |  | 1 |
| CHRNE |  | 1 |
| CHTOP |  | 1 |
| CISH |  | 1 |
| CLCA4 |  | 1 |
| CLCN2 |  | 1 |
| CLEC12B |  | 1 |
| CLEC4F |  | 1 |
| CLGN |  | 1 |
| CLN5 |  | 1 |
| CLP1 |  | 1 |
| CLPSL1 |  | 1 |
| CLTA |  | 1 |
| CLYBL |  | 1 |
| CMIP |  | 1 |
| CNIH2 |  | 1 |
| CNPPD1 |  | 1 |
| COL8A2 |  | 1 |
| COLEC10 |  | 1 |
| COMMD8 |  | 1 |
| COQ7 |  | 1 |
| CORO2B |  | 1 |
| COX15 |  | 1 |
| CPE |  | 1 |
| CPNE7 |  | 1 |
| CPXM2 |  | 1 |
| CRISP2 |  | 1 |
| CSNK1A1L |  | 1 |
| CSRP3 |  | 1 |
| CST4 |  | 1 |
| CTNNA1 |  | 1 |
| CWF19L1 |  | 1 |
| CXCL5 |  | 1 |
| CYB5R3 |  | 1 |
| CYP4F2 |  | 1 |
| CYP7B1 |  | 1 |
| CYTH1 |  | 1 |
| DCLRE1A |  | 1 |
| DDI2 |  | 1 |
| DDRGK1 |  | 1 |
| DDX56 |  | 1 |
| DDX6 |  | 1 |
| DECR2 |  | 1 |
| DEFA4 |  | 1 |
| DEFB4B |  | 1 |
| DEGS1 |  | 1 |
| DEXI |  | 1 |
| DGCR8 |  | 1 |
| DIRAS3 |  | 1 |
| DLL3 |  | 1 |
| DMAP1 |  | 1 |
| DNAJC11 |  | 1 |
| DNAJC5B |  | 1 |
| DOC2A |  | 1 |
| DOK2 |  | 1 |
| DPEP3 |  | 1 |
| DPPA3 |  | 1 |
| DUSP28 |  | 1 |
| E2F2 |  | 1 |
| E2F8 |  | 1 |
| EBI3 |  | 1 |
| EBLN2 |  | 1 |
| ECHDC1 |  | 1 |
| EDN3 |  | 1 |
| EFR3A |  | 1 |
| EIF1AD |  | 1 |
| EIF2B4 |  | 1 |
| ELAVL4 |  | 1 |
| ELMO3 |  | 1 |
| ELOVL5 |  | 1 |
| ELOVL6 |  | 1 |
| ELP4 |  | 1 |
| EMC1 |  | 1 |
| EMC6 |  | 1 |
| EMX2 |  | 1 |
| ENOPH1 |  | 1 |
| ENPEP |  | 1 |
| ENPP4 |  | 1 |
| ENPP5 |  | 1 |
| ENPP6 |  | 1 |
| ENTPD3 |  | 1 |
| ERBB3 |  | 1 |
| ERLEC1 |  | 1 |
| ERRFI1 |  | 1 |
| ESPN |  | 1 |
| ESRP1 |  | 1 |
| ETV7 |  | 1 |
| EZH1 |  | 1 |
| F11R |  | 1 |
| FAHD2A |  | 1 |
| FAHD2B |  | 1 |
| FAM100B |  | 1 |
| FAM160B2 |  | 1 |
| FAM165B |  | 1 |
| FAM166A |  | 1 |
| FAM174A |  | 1 |
| FAM208A |  | 1 |
| FAM3A |  | 1 |
| FAM59A |  | 1 |
| FANCB |  | 1 |
| FBLN7 |  | 1 |
| FBXL5 |  | 1 |
| FBXO21 |  | 1 |
| FBXO43 |  | 1 |
| FDPS |  | 1 |
| FGF1 |  | 1 |
| FGF11 |  | 1 |
| FLII |  | 1 |
| FNIP1 |  | 1 |
| FOXD4L6 |  | 1 |
| FOXN3 |  | 1 |
| FOXP1 |  | 1 |
| FPR2 |  | 1 |
| FRMPD2 |  | 1 |
| FSCN2 |  | 1 |
| FXYD3 |  | 1 |
| FXYD7 |  | 1 |
| GAB3 |  | 1 |
| GABRD |  | 1 |
| GAGE12D |  | 1 |
| GAL3ST1 |  | 1 |
| GALNT5 |  | 1 |
| GALNT8 |  | 1 |
| GALP |  | 1 |
| GBAS |  | 1 |
| GBP2 |  | 1 |
| GCFC2 |  | 1 |
| GCLM |  | 1 |
| GDF9 |  | 1 |
| GDI2 |  | 1 |
| GDPGP1 |  | 1 |
| GJB3 |  | 1 |
| GLIPR1L1 |  | 1 |
| GLRA3 |  | 1 |
| GMFG |  | 1 |
| GNAT1 |  | 1 |
| GNG5 |  | 1 |
| GNGT2 |  | 1 |
| GNS |  | 1 |
| GPBP1L1 |  | 1 |
| GPR137C |  | 1 |
| GPR149 |  | 1 |
| GPR31 |  | 1 |
| GPR4 |  | 1 |
| GPR65 |  | 1 |
| GPR87 |  | 1 |
| GRAMD1B |  | 1 |
| GRPEL2 |  | 1 |
| GSG1L |  | 1 |
| GTF2A1 |  | 1 |
| GYPA |  | 1 |
| GZMH |  | 1 |
| H2AFX |  | 1 |
| HADHA |  | 1 |
| HCN3 |  | 1 |
| HCRTR2 |  | 1 |
| HDDC3 |  | 1 |
| HEXIM2 |  | 1 |
| HHATL |  | 1 |
| HIST2H2AA3 |  | 1 |
| HIST2H2BE |  | 1 |
| HIST2H4B |  | 1 |
| HMMR |  | 1 |
| HNRNPH1 |  | 1 |
| HNRNPH2 |  | 1 |
| HNRPLL |  | 1 |
| HSD17B1 |  | 1 |
| HSD17B12 |  | 1 |
| HSF4 |  | 1 |
| HTRA1 |  | 1 |
| HYLS1 |  | 1 |
| ICAM1 |  | 1 |
| ICAM3 |  | 1 |
| IDI2 |  | 1 |
| IDS |  | 1 |
| IER2 |  | 1 |
| IFI30 |  | 1 |
| IFITM2 |  | 1 |
| IFNA14 |  | 1 |
| IFNA8 |  | 1 |
| IFNAR1 |  | 1 |
| IFNE |  | 1 |
| IFNK |  | 1 |
| IFT43 |  | 1 |
| IGFBP3 |  | 1 |
| IGFBP6 |  | 1 |
| IL17RA |  | 1 |
| IL25 |  | 1 |
| IL28RA |  | 1 |
| IL32 |  | 1 |
| IL33 |  | 1 |
| IL7R |  | 1 |
| INO80C |  | 1 |
| IRX3 |  | 1 |
| ISOC1 |  | 1 |
| ITIH5 |  | 1 |
| ITLN2 |  | 1 |
| JSRP1 |  | 1 |
| KANK1 |  | 1 |
| KAT7 |  | 1 |
| KATNAL1 |  | 1 |
| KAZALD1 |  | 1 |
| KBTBD12 |  | 1 |
| KCNC1 |  | 1 |
| KCND3 |  | 1 |
| KCNJ16 |  | 1 |
| KCNK9 |  | 1 |
| KCNQ2 |  | 1 |
| KCTD13 |  | 1 |
| KDSR |  | 1 |
| KIAA0284 |  | 1 |
| KIAA1161 |  | 1 |
| KIAA1609 |  | 1 |
| KIR2DS1 |  | 1 |
| KLHDC3 |  | 1 |
| KLHL8 |  | 1 |
| KLKB1 |  | 1 |
| KLRC3 |  | 1 |
| KLRK1 |  | 1 |
| KNG1 |  | 1 |
| KPNA3 |  | 1 |
| KPRP |  | 1 |
| KPTN |  | 1 |
| KRBA1 |  | 1 |
| KRT8 |  | 1 |
| KRT81 |  | 1 |
| KRT83 |  | 1 |
| LAMTOR1 |  | 1 |
| LCN15 |  | 1 |
| LGALS3BP |  | 1 |
| LGALSL |  | 1 |
| LIMS1 |  | 1 |
| LLGL2 |  | 1 |
| LLPH |  | 1 |
| LMAN2L |  | 1 |
| LMBRD2 |  | 1 |
| LPAR4 |  | 1 |
| LRRC25 |  | 1 |
| LRRC52 |  | 1 |
| LRRC57 |  | 1 |
| LRRTM1 |  | 1 |
| LSM1 |  | 1 |
| LSM10 |  | 1 |
| LTA |  | 1 |
| MAB21L2 |  | 1 |
| MAGEB6 |  | 1 |
| MAGEF1 |  | 1 |
| MAGOH |  | 1 |
| MAK16 |  | 1 |
| MAMDC4 |  | 1 |
| MAP4K1 |  | 1 |
| MAPK15 |  | 1 |
| MARCH1 |  | 1 |
| MARCH4 |  | 1 |
| MARCH6 |  | 1 |
| MAS1L |  | 1 |
| MATN4 |  | 1 |
| MBD5 |  | 1 |
| MBNL1 |  | 1 |
| MCCC1 |  | 1 |
| MCPH1 |  | 1 |
| MDGA2 |  | 1 |
| MEF2A |  | 1 |
| MEF2D |  | 1 |
| MEMO1 |  | 1 |
| MEP1A |  | 1 |
| MESP2 |  | 1 |
| METTL22 |  | 1 |
| MFAP3 |  | 1 |
| MGAT4B |  | 1 |
| MGAT5B |  | 1 |
| MKRN1 |  | 1 |
| MMP23A |  | 1 |
| MOGAT1 |  | 1 |
| MPP4 |  | 1 |
| MRPL3 |  | 1 |
| MRPL43 |  | 1 |
| MS4A6A |  | 1 |
| MTAP |  | 1 |
| MTBP |  | 1 |
| MTMR11 |  | 1 |
| MUC7 |  | 1 |
| MYEOV |  | 1 |
| MYEOV2 |  | 1 |
| MYO1B |  | 1 |
| MYO1C |  | 1 |
| NAGS |  | 1 |
| NALCN |  | 1 |
| NCKIPSD |  | 1 |
| NDC80 |  | 1 |
| NDP |  | 1 |
| NDUFAB1 |  | 1 |
| NDUFAF5 |  | 1 |
| NDUFB1 |  | 1 |
| NDUFS1 |  | 1 |
| NEK6 |  | 1 |
| NFE2L2 |  | 1 |
| NGB |  | 1 |
| NGEF |  | 1 |
| NIT1 |  | 1 |
| NLRP9 |  | 1 |
| NME6 |  | 1 |
| NOP58 |  | 1 |
| NOX4 |  | 1 |
| NPFFR1 |  | 1 |
| NPHS1 |  | 1 |
| NPTXR |  | 1 |
| NPVF |  | 1 |
| NPY2R |  | 1 |
| NRBF2 |  | 1 |
| NUDCD2 |  | 1 |
| NUDT22 |  | 1 |
| NUP62 |  | 1 |
| NXPH3 |  | 1 |
| OGDHL |  | 1 |
| OIT3 |  | 1 |
| OLFML2A |  | 1 |
| OPN1SW |  | 1 |
| OR10H2 |  | 1 |
| OR10H3 |  | 1 |
| OR11H12 |  | 1 |
| OR14A16 |  | 1 |
| OR2A12 |  | 1 |
| OR2G6 |  | 1 |
| OR2J2 |  | 1 |
| OR2S2 |  | 1 |
| OR2W3 |  | 1 |
| OR4Q3 |  | 1 |
| OR52N5 |  | 1 |
| OR5J2 |  | 1 |
| OR6N1 |  | 1 |
| OR7C2 |  | 1 |
| OR7D2 |  | 1 |
| OSGEP |  | 1 |
| OSGIN1 |  | 1 |
| OXNAD1 |  | 1 |
| OXSM |  | 1 |
| PADI6 |  | 1 |
| PAK4 |  | 1 |
| PANX3 |  | 1 |
| PC |  | 1 |
| PCCA |  | 1 |
| PCDHGA12 |  | 1 |
| PCK2 |  | 1 |
| PCP4L1 |  | 1 |
| PDDC1 |  | 1 |
| PDE10A |  | 1 |
| PDE4DIP |  | 1 |
| PDLIM3 |  | 1 |
| PGAP1 |  | 1 |
| PHB |  | 1 |
| PHEX |  | 1 |
| PHF21A |  | 1 |
| PIGF |  | 1 |
| PIGP |  | 1 |
| PIK3R6 |  | 1 |
| PINX1 |  | 1 |
| PITHD1 |  | 1 |
| PITPNA |  | 1 |
| PIWIL4 |  | 1 |
| PKNOX1 |  | 1 |
| PLEKHA1 |  | 1 |
| PNLIPRP1 |  | 1 |
| POFUT1 |  | 1 |
| POLE2 |  | 1 |
| POLR2J |  | 1 |
| PON2 |  | 1 |
| PPIL2 |  | 1 |
| PPP1R3B |  | 1 |
| PPP1R7 |  | 1 |
| PPP2R3A |  | 1 |
| PPP4R2 |  | 1 |
| PRAF2 |  | 1 |
| PRAME |  | 1 |
| PRDX4 |  | 1 |
| PRDX5 |  | 1 |
| PRELID2 |  | 1 |
| PRNP |  | 1 |
| PRR14 |  | 1 |
| PRRG1 |  | 1 |
| PRSS53 |  | 1 |
| PSEN2 |  | 1 |
| PSMA4 |  | 1 |
| PSMC2 |  | 1 |
| PSMC4 |  | 1 |
| PTGIR |  | 1 |
| PTGIS |  | 1 |
| PTPRS |  | 1 |
| PWP2 |  | 1 |
| PYROXD2 |  | 1 |
| QSOX1 |  | 1 |
| RAB27A |  | 1 |
| RAB30 |  | 1 |
| RAB40C |  | 1 |
| RAB41 |  | 1 |
| RAB9B |  | 1 |
| RALY |  | 1 |
| RANBP3L |  | 1 |
| RANGRF |  | 1 |
| RASGRP2 |  | 1 |
| RASSF3 |  | 1 |
| RBX1 |  | 1 |
| RC3H2 |  | 1 |
| RCN1 |  | 1 |
| RDH12 |  | 1 |
| REEP6 |  | 1 |
| REG3G |  | 1 |
| RELL1 |  | 1 |
| REN |  | 1 |
| RHCG |  | 1 |
| RHEB |  | 1 |
| RHOC |  | 1 |
| RICTOR |  | 1 |
| RIMKLA |  | 1 |
| RIN2 |  | 1 |
| RNASE10 |  | 1 |
| RNF133 |  | 1 |
| RNF144A |  | 1 |
| RNF170 |  | 1 |
| RNF185 |  | 1 |
| RPGR |  | 1 |
| RPH3AL |  | 1 |
| RPL18A |  | 1 |
| RPL35A |  | 1 |
| RPL39L |  | 1 |
| RPP14 |  | 1 |
| RPS6KB2 |  | 1 |
| RPTOR |  | 1 |
| RSBN1L |  | 1 |
| RSPO1 |  | 1 |
| RWDD2A |  | 1 |
| RYK |  | 1 |
| S100P |  | 1 |
| S1PR3 |  | 1 |
| SALL4 |  | 1 |
| SBDS |  | 1 |
| SCARB2 |  | 1 |
| SCN3B |  | 1 |
| SDC1 |  | 1 |
| SEC14L3 |  | 1 |
| SEMA3E |  | 1 |
| SERHL2 |  | 1 |
| SERPINA3 |  | 1 |
| SERPINA7 |  | 1 |
| SERPINB5 |  | 1 |
| SERPINB9 |  | 1 |
| SESN3 |  | 1 |
| SF1 |  | 1 |
| SFXN4 |  | 1 |
| SH3PXD2B |  | 1 |
| SHC4 |  | 1 |
| SIX5 |  | 1 |
| SLC16A11 |  | 1 |
| SLC16A8 |  | 1 |
| SLC17A4 |  | 1 |
| SLC25A52 |  | 1 |
| SLC26A1 |  | 1 |
| SLC26A11 |  | 1 |
| SLC2A2 |  | 1 |
| SLC35C1 |  | 1 |
| SLC5A7 |  | 1 |
| SMN2 |  | 1 |
| SMR3B |  | 1 |
| SMU1 |  | 1 |
| SNF8 |  | 1 |
| SNRNP48 |  | 1 |
| SNRPA1 |  | 1 |
| SNRPB |  | 1 |
| SNRPB2 |  | 1 |
| SNX14 |  | 1 |
| SNX6 |  | 1 |
| SOD2 |  | 1 |
| SOLH |  | 1 |
| SOX17 |  | 1 |
| SP6 |  | 1 |
| SPACA1 |  | 1 |
| SPAG7 |  | 1 |
| SPANXE |  | 1 |
| SPATA12 |  | 1 |
| SPDYC |  | 1 |
| SPESP1 |  | 1 |
| SPG7 |  | 1 |
| SPINK1 |  | 1 |
| SPINT1 |  | 1 |
| SPINT2 |  | 1 |
| SPP1 |  | 1 |
| SRBD1 |  | 1 |
| SREK1IP1 |  | 1 |
| SRSF5 |  | 1 |
| ST6GALNAC1 |  | 1 |
| STAP2 |  | 1 |
| STAT6 |  | 1 |
| STIM1 |  | 1 |
| STK16 |  | 1 |
| STK25 |  | 1 |
| STK32A |  | 1 |
| STK38L |  | 1 |
| SULT1A4 |  | 1 |
| SULT4A1 |  | 1 |
| SULT6B1 |  | 1 |
| SVOPL |  | 1 |
| SWAP70 |  | 1 |
| SWSAP1 |  | 1 |
| SYNDIG1 |  | 1 |
| SYNGR4 |  | 1 |
| SYP |  | 1 |
| TAF1A |  | 1 |
| TAF2 |  | 1 |
| TAP2 |  | 1 |
| TBC1D22B |  | 1 |
| TBCB |  | 1 |
| TCEAL5 |  | 1 |
| TCN2 |  | 1 |
| TCTA |  | 1 |
| TDRD12 |  | 1 |
| TEAD1 |  | 1 |
| TEAD2 |  | 1 |
| TECTB |  | 1 |
| TESK2 |  | 1 |
| TFDP3 |  | 1 |
| TFF2 |  | 1 |
| TFR2 |  | 1 |
| TH1L |  | 1 |
| TICAM1 |  | 1 |
| TIMD4 |  | 1 |
| TINF2 |  | 1 |
| TIPIN |  | 1 |
| TLCD1 |  | 1 |
| TLE2 |  | 1 |
| TLR3 |  | 1 |
| TM4SF18 |  | 1 |
| TMC5 |  | 1 |
| TMEFF1 |  | 1 |
| TMEM126A |  | 1 |
| TMEM130 |  | 1 |
| TMEM159 |  | 1 |
| TMEM176B |  | 1 |
| TMEM177 |  | 1 |
| TMEM208 |  | 1 |
| TMIGD1 |  | 1 |
| TMOD1 |  | 1 |
| TMX2 |  | 1 |
| TNFAIP3 |  | 1 |
| TOMM5 |  | 1 |
| TP53TG5 |  | 1 |
| TPMT |  | 1 |
| TPO |  | 1 |
| TPSAB1 |  | 1 |
| TPX2 |  | 1 |
| TRAF4 |  | 1 |
| TRAP1 |  | 1 |
| TRAPPC1 |  | 1 |
| TRAT1 |  | 1 |
| TRDMT1 |  | 1 |
| TREML2 |  | 1 |
| TRPA1 |  | 1 |
| TRUB2 |  | 1 |
| TSEN34 |  | 1 |
| TSHR |  | 1 |
| TSHZ3 |  | 1 |
| TSPAN15 |  | 1 |
| TTYH2 |  | 1 |
| TUBD1 |  | 1 |
| TXLNA |  | 1 |
| TXNDC11 |  | 1 |
| TXNDC8 |  | 1 |
| TYRP1 |  | 1 |
| UBA5 |  | 1 |
| UBE2D4 |  | 1 |
| UBE3B |  | 1 |
| UGT1A8 |  | 1 |
| ULK4 |  | 1 |
| UPK1A |  | 1 |
| UROS |  | 1 |
| USP39 |  | 1 |
| USP54 |  | 1 |
| VDAC2 |  | 1 |
| VEGFB |  | 1 |
| VPS25 |  | 1 |
| VPS37A |  | 1 |
| VWA5A |  | 1 |
| WARS |  | 1 |
| WBSCR27 |  | 1 |
| WDR36 |  | 1 |
| WDR5 |  | 1 |
| WDR91 |  | 1 |
| WFDC10A |  | 1 |
| WIF1 |  | 1 |
| WISP2 |  | 1 |
| WNK1 |  | 1 |
| WNT5A |  | 1 |
| WWP1 |  | 1 |
| XIRP2 |  | 1 |
| XKRX |  | 1 |
| XPA |  | 1 |
| XPO6 |  | 1 |
| ZCRB1 |  | 1 |
| ZDHHC13 |  | 1 |
| ZFAND2B |  | 1 |
| ZFAND5 |  | 1 |
| ZFP28 |  | 1 |
| ZGPAT |  | 1 |
| ZIM2 |  | 1 |
| ZNF214 |  | 1 |
| ZNF284 |  | 1 |
| ZNF285 |  | 1 |
| ZNF324 |  | 1 |
| ZNF33A |  | 1 |
| ZNF488 |  | 1 |
| ZNF490 |  | 1 |
| ZNF510 |  | 1 |
| ZNF543 |  | 1 |
| ZNF69 |  | 1 |
| ZNF7 |  | 1 |
| ZNF708 |  | 1 |
| ZNF770 |  | 1 |
| ACADL |  | 3 |
| ACBD6 |  | 3 |
| ACOT11 |  | 3 |
| ACPL2 |  | 3 |
| ADAM2 |  | 3 |
| ADCK1 |  | 3 |
| ADPRH |  | 3 |
| ADRBK1 |  | 3 |
| AGXT2L1 |  | 3 |
| AGXT2L2 |  | 3 |
| AHNAK2 |  | 3 |
| AHSG |  | 3 |
| AKR1C1 |  | 3 |
| ALDH5A1 |  | 3 |
| AMN1 |  | 3 |
| AMPD2 |  | 3 |
| AMTN |  | 3 |
| AMY2B |  | 3 |
| ANAPC5 |  | 3 |
| ANGPTL3 |  | 3 |
| ANKRD49 |  | 3 |
| ANKRD5 |  | 3 |
| ANXA4 |  | 3 |
| APBB2 |  | 3 |
| APPL1 |  | 3 |
| ARMCX6 |  | 3 |
| ARSG |  | 3 |
| ASAH1 |  | 3 |
| ASB4 |  | 3 |
| ASPHD1 |  | 3 |
| ASTL |  | 3 |
| ASZ1 |  | 3 |
| ATF7IP |  | 3 |
| ATL2 |  | 3 |
| ATP2A1 |  | 3 |
| ATP6V0A2 |  | 3 |
| B4GALNT3 |  | 3 |
| BCAN |  | 3 |
| BGN |  | 3 |
| BHMT2 |  | 3 |
| BRD1 |  | 3 |
| BRD4 |  | 3 |
| C10orf71 |  | 3 |
| C10orf82 |  | 3 |
| C14orf80 |  | 3 |
| C15orf54 |  | 3 |
| C16orf3 |  | 3 |
| C16orf73 |  | 3 |
| C17orf47 |  | 3 |
| C1orf168 |  | 3 |
| C1orf212 |  | 3 |
| C22orf13 |  | 3 |
| C22orf39 |  | 3 |
| C22orf46 |  | 3 |
| C2CD2 |  | 3 |
| C2orf47 |  | 3 |
| C2orf54 |  | 3 |
| C3AR1 |  | 3 |
| C3orf27 |  | 3 |
| C3orf37 |  | 3 |
| C4orf33 |  | 3 |
| C5orf44 |  | 3 |
| C6orf89 |  | 3 |
| C9orf142 |  | 3 |
| C9orf170 |  | 3 |
| C9orf171 |  | 3 |
| CA14 |  | 3 |
| CACNA2D4 |  | 3 |
| CALML4 |  | 3 |
| CAMKMT |  | 3 |
| CANT1 |  | 3 |
| CAPS2 |  | 3 |
| CCDC14 |  | 3 |
| CCDC41 |  | 3 |
| CCDC43 |  | 3 |
| CCDC60 |  | 3 |
| CCDC72 |  | 3 |
| CCHCR1 |  | 3 |
| CCND3 |  | 3 |
| CCR6 |  | 3 |
| CCZ1B |  | 3 |
| CD164L2 |  | 3 |
| CD3E |  | 3 |
| CD40 |  | 3 |
| CD55 |  | 3 |
| CD8B |  | 3 |
| CDC25B |  | 3 |
| CGB7 |  | 3 |
| CHI3L1 |  | 3 |
| CHL1 |  | 3 |
| CHRNB4 |  | 3 |
| CIB4 |  | 3 |
| CLPTM1 |  | 3 |
| CLSTN2 |  | 3 |
| CNOT10 |  | 3 |
| COG7 |  | 3 |
| COQ3 |  | 3 |
| CPA6 |  | 3 |
| CPM |  | 3 |
| CRIP2 |  | 3 |
| CSRP2BP |  | 3 |
| CXorf1 |  | 3 |
| CYP11B2 |  | 3 |
| CYP4F8 |  | 3 |
| DAP |  | 3 |
| DDX18 |  | 3 |
| DDX31 |  | 3 |
| DDX43 |  | 3 |
| DEPDC5 |  | 3 |
| DGAT2 |  | 3 |
| DHRS1 |  | 3 |
| DHX57 |  | 3 |
| DIDO1 |  | 3 |
| DLC1 |  | 3 |
| DMP1 |  | 3 |
| DNAH14 |  | 3 |
| DNAJB6 |  | 3 |
| DNAJC14 |  | 3 |
| DNASE2B |  | 3 |
| DOCK4 |  | 3 |
| DSN1 |  | 3 |
| DUS4L |  | 3 |
| DUSP19 |  | 3 |
| DUSP6 |  | 3 |
| EAPP |  | 3 |
| EDNRA |  | 3 |
| EIF4EBP2 |  | 3 |
| ENPP7 |  | 3 |
| EPB41L2 |  | 3 |
| EPHX1 |  | 3 |
| ERGIC1 |  | 3 |
| ERI3 |  | 3 |
| ESR2 |  | 3 |
| ETHE1 |  | 3 |
| ETS1 |  | 3 |
| EVI2B |  | 3 |
| F11 |  | 3 |
| F8 |  | 3 |
| F8A1 |  | 3 |
| FAAH2 |  | 3 |
| FAM102A |  | 3 |
| FAM111A |  | 3 |
| FAM18A |  | 3 |
| FAM190B |  | 3 |
| FAM45A |  | 3 |
| FAM96A |  | 3 |
| FAN1 |  | 3 |
| FBXO24 |  | 3 |
| FBXO40 |  | 3 |
| FCGR3A |  | 3 |
| FCRL4 |  | 3 |
| FEZ1 |  | 3 |
| FOXD4L4 |  | 3 |
| FOXN2 |  | 3 |
| FUT9 |  | 3 |
| FXC1 |  | 3 |
| FZD10 |  | 3 |
| G6PC2 |  | 3 |
| GADD45B |  | 3 |
| GALNT10 |  | 3 |
| GAN |  | 3 |
| GATS |  | 3 |
| GCA |  | 3 |
| GDA |  | 3 |
| GGH |  | 3 |
| GJC1 |  | 3 |
| GMCL1 |  | 3 |
| GNAI1 |  | 3 |
| GNB1L |  | 3 |
| GORASP1 |  | 3 |
| GP5 |  | 3 |
| GPR139 |  | 3 |
| GPR142 |  | 3 |
| GPR151 |  | 3 |
| GPR180 |  | 3 |
| GPR34 |  | 3 |
| GSN |  | 3 |
| GSTCD |  | 3 |
| GTF2A2 |  | 3 |
| GTPBP8 |  | 3 |
| HAND2 |  | 3 |
| HBE1 |  | 3 |
| HBG2 |  | 3 |
| HBS1L |  | 3 |
| HBZ |  | 3 |
| HDAC3 |  | 3 |
| HENMT1 |  | 3 |
| HES6 |  | 3 |
| HIST1H2AC |  | 3 |
| HIST1H3A |  | 3 |
| HIST3H2A |  | 3 |
| HLA-B |  | 3 |
| HOXD10 |  | 3 |
| HS6ST2 |  | 3 |
| HSD17B13 |  | 3 |
| HSD17B2 |  | 3 |
| HSPA2 |  | 3 |
| HUS1B |  | 3 |
| ICA1 |  | 3 |
| IDH3G |  | 3 |
| IGFBP1 |  | 3 |
| IGFBP7 |  | 3 |
| IGSF10 |  | 3 |
| IGSF9 |  | 3 |
| IL17F |  | 3 |
| ILF3 |  | 3 |
| ILK |  | 3 |
| IQCF3 |  | 3 |
| IQUB |  | 3 |
| ITGA9 |  | 3 |
| KANSL3 |  | 3 |
| KBTBD10 |  | 3 |
| KBTBD4 |  | 3 |
| KCNK13 |  | 3 |
| KCNK4 |  | 3 |
| KIAA0040 |  | 3 |
| KIF16B |  | 3 |
| KIR2DL1 |  | 3 |
| KIRREL |  | 3 |
| KLHL11 |  | 3 |
| KMO |  | 3 |
| KRT12 |  | 3 |
| KRT3 |  | 3 |
| KRT80 |  | 3 |
| KRTAP26-1 |  | 3 |
| LAPTM5 |  | 3 |
| LARP6 |  | 3 |
| LARP7 |  | 3 |
| LBX2 |  | 3 |
| LCE3E |  | 3 |
| LIN28A |  | 3 |
| LIPF |  | 3 |
| LMAN1 |  | 3 |
| LOXL1 |  | 3 |
| LPCAT4 |  | 3 |
| LPL |  | 3 |
| LRP1 |  | 3 |
| LRRN2 |  | 3 |
| LRRN3 |  | 3 |
| LRTM1 |  | 3 |
| LSR |  | 3 |
| LYPD6B |  | 3 |
| MANEA |  | 3 |
| MAOA |  | 3 |
| MDGA1 |  | 3 |
| MED1 |  | 3 |
| MEN1 |  | 3 |
| MEPCE |  | 3 |
| METTL14 |  | 3 |
| MFAP1 |  | 3 |
| MIA3 |  | 3 |
| MICA |  | 3 |
| MIOX |  | 3 |
| MMACHC |  | 3 |
| MMP19 |  | 3 |
| MMP3 |  | 3 |
| MND1 |  | 3 |
| MNS1 |  | 3 |
| MORF4L2 |  | 3 |
| MPV17 |  | 3 |
| MPZL2 |  | 3 |
| MRGPRF |  | 3 |
| MRPL50 |  | 3 |
| MTFMT |  | 3 |
| MTIF3 |  | 3 |
| N4BP2L2 |  | 3 |
| NAALAD2 |  | 3 |
| NBPF3 |  | 3 |
| NCF1 |  | 3 |
| NDST3 |  | 3 |
| NEUROD1 |  | 3 |
| NKX3-1 |  | 3 |
| NKX3-2 |  | 3 |
| NOLC1 |  | 3 |
| NPL |  | 3 |
| NPSR1 |  | 3 |
| NR1I3 |  | 3 |
| NRP2 |  | 3 |
| NT5DC3 |  | 3 |
| NT5E |  | 3 |
| NXF5 |  | 3 |
| OPN1MW |  | 3 |
| OPN1MW2 |  | 3 |
| OR10H1 |  | 3 |
| OR10J5 |  | 3 |
| OR13C5 |  | 3 |
| OR2M4 |  | 3 |
| OR3A1 |  | 3 |
| OR4S2 |  | 3 |
| OR52L1 |  | 3 |
| OR5C1 |  | 3 |
| OR6C4 |  | 3 |
| OR6M1 |  | 3 |
| OR7E24 |  | 3 |
| P2RY2 |  | 3 |
| P4HA2 |  | 3 |
| PADI1 |  | 3 |
| PAQR8 |  | 3 |
| PCDH12 |  | 3 |
| PCTP |  | 3 |
| PDE8B |  | 3 |
| PDE9A |  | 3 |
| PDS5B |  | 3 |
| PDZD8 |  | 3 |
| PER2 |  | 3 |
| PEX11B |  | 3 |
| PGP |  | 3 |
| PHF20 |  | 3 |
| PI15 |  | 3 |
| PIEZO2 |  | 3 |
| PIGH |  | 3 |
| PIGT |  | 3 |
| PIH1D1 |  | 3 |
| PIWIL2 |  | 3 |
| PLAC8L1 |  | 3 |
| PLBD1 |  | 3 |
| PLCXD2 |  | 3 |
| PLEKHO1 |  | 3 |
| PNKP |  | 3 |
| PNPLA1 |  | 3 |
| POLR2E |  | 3 |
| POLR2F |  | 3 |
| POLR2M |  | 3 |
| PON1 |  | 3 |
| POU2F2 |  | 3 |
| POU5F1 |  | 3 |
| PPEF2 |  | 3 |
| PPID |  | 3 |
| PPP1R8 |  | 3 |
| PPP2R5C |  | 3 |
| PPP3CC |  | 3 |
| PRCC |  | 3 |
| PRDM12 |  | 3 |
| PRKX |  | 3 |
| PRR25 |  | 3 |
| PRRC2B |  | 3 |
| PRSS21 |  | 3 |
| PSMA2 |  | 3 |
| PSMC1 |  | 3 |
| PSMD3 |  | 3 |
| PSPC1 |  | 3 |
| PTP4A1 |  | 3 |
| PVRL4 |  | 3 |
| RAB7L1 |  | 3 |
| RAD23B |  | 3 |
| RAMP2 |  | 3 |
| RASD2 |  | 3 |
| RBBP4 |  | 3 |
| RBM17 |  | 3 |
| RBM4 |  | 3 |
| RCBTB1 |  | 3 |
| REEP2 |  | 3 |
| RGL4 |  | 3 |
| RGN |  | 3 |
| RPE |  | 3 |
| RPL37A |  | 3 |
| RRP7A |  | 3 |
| RSU1 |  | 3 |
| RTBDN |  | 3 |
| RUVBL2 |  | 3 |
| RXFP1 |  | 3 |
| SAMD7 |  | 3 |
| SCAMP2 |  | 3 |
| SCGB1A1 |  | 3 |
| SCML1 |  | 3 |
| SCP2 |  | 3 |
| SDHD |  | 3 |
| SEMA6A |  | 3 |
| SEMA7A |  | 3 |
| SENP6 |  | 3 |
| SEPHS1 |  | 3 |
| SFSWAP |  | 3 |
| SGK1 |  | 3 |
| SH3D19 |  | 3 |
| SLAMF8 |  | 3 |
| SLC13A2 |  | 3 |
| SLC16A14 |  | 3 |
| SLC25A5 |  | 3 |
| SLC25A6 |  | 3 |
| SLC2A12 |  | 3 |
| SLC2A13 |  | 3 |
| SLC35A4 |  | 3 |
| SLC35A5 |  | 3 |
| SLC35D2 |  | 3 |
| SLC35F5 |  | 3 |
| SLC37A4 |  | 3 |
| SLC44A3 |  | 3 |
| SLC44A5 |  | 3 |
| SLC47A1 |  | 3 |
| SLC5A3 |  | 3 |
| SLU7 |  | 3 |
| SMEK1 |  | 3 |
| SMG8 |  | 3 |
| SMTNL2 |  | 3 |
| SNRPE |  | 3 |
| SNX33 |  | 3 |
| SOS1 |  | 3 |
| SOX10 |  | 3 |
| SPAG11A |  | 3 |
| SPAG9 |  | 3 |
| SPAM1 |  | 3 |
| SPARCL1 |  | 3 |
| SPATA22 |  | 3 |
| SPCS2 |  | 3 |
| SPOPL |  | 3 |
| SPRY2 |  | 3 |
| SPTLC3 |  | 3 |
| SRL |  | 3 |
| SRY |  | 3 |
| ST6GALNAC3 |  | 3 |
| STAMBPL1 |  | 3 |
| STON1 |  | 3 |
| STOX1 |  | 3 |
| STX19 |  | 3 |
| STXBP3 |  | 3 |
| STXBP5 |  | 3 |
| SYNPO |  | 3 |
| SYT16 |  | 3 |
| SYT9 |  | 3 |
| TACC1 |  | 3 |
| TACR1 |  | 3 |
| TAGAP |  | 3 |
| TBC1D3B |  | 3 |
| TCF4 |  | 3 |
| TDRD3 |  | 3 |
| TEN1 |  | 3 |
| TET2 |  | 3 |
| TGM5 |  | 3 |
| THEM5 |  | 3 |
| THRAP3 |  | 3 |
| TIMM21 |  | 3 |
| TIMP3 |  | 3 |
| TLE6 |  | 3 |
| TM4SF4 |  | 3 |
| TMEM139 |  | 3 |
| TMEM140 |  | 3 |
| TMEM14B |  | 3 |
| TMEM150A |  | 3 |
| TMEM185B |  | 3 |
| TMEM25 |  | 3 |
| TMPRSS12 |  | 3 |
| TMPRSS3 |  | 3 |
| TMPRSS9 |  | 3 |
| TMSB10 |  | 3 |
| TNXB |  | 3 |
| TP53TG3 |  | 3 |
| TRAF1 |  | 3 |
| TREM2 |  | 3 |
| TREML1 |  | 3 |
| TRIM39 |  | 3 |
| TRIM5 |  | 3 |
| TRMT61B |  | 3 |
| TRPC4 |  | 3 |
| TSHB |  | 3 |
| TSPAN1 |  | 3 |
| TTC39C |  | 3 |
| TTLL2 |  | 3 |
| TTLL9 |  | 3 |
| TUBA1C |  | 3 |
| TUBA4A |  | 3 |
| TUBB |  | 3 |
| UBA1 |  | 3 |
| UBAC2 |  | 3 |
| UBE2L6 |  | 3 |
| UBR2 |  | 3 |
| UCP1 |  | 3 |
| UGT2B11 |  | 3 |
| UIMC1 |  | 3 |
| USP28 |  | 3 |
| UXT |  | 3 |
| VAT1L |  | 3 |
| VIPR2 |  | 3 |
| VPS45 |  | 3 |
| VPS54 |  | 3 |
| WBP4 |  | 3 |
| WDR45 |  | 3 |
| WDR46 |  | 3 |
| WDR47 |  | 3 |
| WFDC10B |  | 3 |
| WFDC11 |  | 3 |
| WNT1 |  | 3 |
| XCL1 |  | 3 |
| XKR3 |  | 3 |
| YSK4 |  | 3 |
| YWHAZ |  | 3 |
| ZBTB25 |  | 3 |
| ZCCHC17 |  | 3 |
| ZG16B |  | 3 |
| ZMYM6 |  | 3 |
| ZNF273 |  | 3 |
| ZNF350 |  | 3 |
| ZNF454 |  | 3 |
| ZNF491 |  | 3 |
| ZNF517 |  | 3 |
| ZNF554 |  | 3 |
| ZNF641 |  | 3 |
| ZNF692 |  | 3 |
| ZNF706 |  | 3 |
| ZNRD1 |  | 3 |
| 41883 |  | 4 |
| AAMP |  | 4 |
| ABCB9 |  | 4 |
| ACMSD |  | 4 |
| ACN9 |  | 4 |
| ACOT6 |  | 4 |
| ACTR3 |  | 4 |
| ADAMTS15 |  | 4 |
| AFM |  | 4 |
| AGGF1 |  | 4 |
| AJAP1 |  | 4 |
| AKR1D1 |  | 4 |
| AKT1S1 |  | 4 |
| ALCAM |  | 4 |
| ALDH1L1 |  | 4 |
| ALPPL2 |  | 4 |
| AMT |  | 4 |
| ANKRD33 |  | 4 |
| AP1M2 |  | 4 |
| AP3S1 |  | 4 |
| APEX2 |  | 4 |
| APOL5 |  | 4 |
| AQP12A |  | 4 |
| ASIC1 |  | 4 |
| ASIC2 |  | 4 |
| ATP1B2 |  | 4 |
| ATP5C1 |  | 4 |
| B3GNT1 |  | 4 |
| B3GNT8 |  | 4 |
| BCAR3 |  | 4 |
| BCOR |  | 4 |
| BPIFB1 |  | 4 |
| BRAF |  | 4 |
| BTN3A2 |  | 4 |
| BTNL3 |  | 4 |
| C10orf12 |  | 4 |
| C11orf49 |  | 4 |
| C12orf12 |  | 4 |
| C14orf101 |  | 4 |
| C15orf59 |  | 4 |
| C16orf54 |  | 4 |
| C16orf72 |  | 4 |
| C17orf61 |  | 4 |
| C17orf81 |  | 4 |
| C19orf25 |  | 4 |
| C1orf210 |  | 4 |
| C3orf75 |  | 4 |
| C5orf51 |  | 4 |
| C5orf64 |  | 4 |
| C6orf165 |  | 4 |
| C9orf106 |  | 4 |
| CABYR |  | 4 |
| CACNG2 |  | 4 |
| CAMLG |  | 4 |
| CAPZA2 |  | 4 |
| CCDC107 |  | 4 |
| CCDC68 |  | 4 |
| CCK |  | 4 |
| CD4 |  | 4 |
| CD99L2 |  | 4 |
| CDCA7 |  | 4 |
| CDHR3 |  | 4 |
| CDK17 |  | 4 |
| CDKN2A |  | 4 |
| CELA2A |  | 4 |
| CELF4 |  | 4 |
| CHCHD3 |  | 4 |
| CHIC1 |  | 4 |
| CHST7 |  | 4 |
| CLDN19 |  | 4 |
| CLEC18A |  | 4 |
| CLEC5A |  | 4 |
| CMTM2 |  | 4 |
| CNOT6L |  | 4 |
| COX18 |  | 4 |
| CSF2 |  | 4 |
| CSMD2 |  | 4 |
| CSN3 |  | 4 |
| CST3 |  | 4 |
| CSTF2T |  | 4 |
| CTSC |  | 4 |
| CYP26B1 |  | 4 |
| D2HGDH |  | 4 |
| DAGLB |  | 4 |
| DAND5 |  | 4 |
| DARS2 |  | 4 |
| DDB2 |  | 4 |
| DEFB113 |  | 4 |
| DEGS2 |  | 4 |
| DENND2A |  | 4 |
| DENND2C |  | 4 |
| DGCR14 |  | 4 |
| DHFRL1 |  | 4 |
| DHX34 |  | 4 |
| DIABLO |  | 4 |
| DIRAS2 |  | 4 |
| DNAH6 |  | 4 |
| DNAJC17 |  | 4 |
| DRAM1 |  | 4 |
| DUSP16 |  | 4 |
| DUSP4 |  | 4 |
| E2F6 |  | 4 |
| EDDM3A |  | 4 |
| EDIL3 |  | 4 |
| EEF1A1 |  | 4 |
| ELF2 |  | 4 |
| ELOVL1 |  | 4 |
| EPB41L4B |  | 4 |
| ERBB2 |  | 4 |
| ERP44 |  | 4 |
| ETFA |  | 4 |
| EXOC7 |  | 4 |
| FAM173B |  | 4 |
| FAM207A |  | 4 |
| FAM221A |  | 4 |
| FAM40A |  | 4 |
| FAM71F2 |  | 4 |
| FOLR1 |  | 4 |
| FOXP3 |  | 4 |
| FSTL1 |  | 4 |
| G6PD |  | 4 |
| GALNT6 |  | 4 |
| GJA10 |  | 4 |
| GLRX |  | 4 |
| GLT6D1 |  | 4 |
| GMPR2 |  | 4 |
| GPHA2 |  | 4 |
| GPN1 |  | 4 |
| GPR20 |  | 4 |
| GPR45 |  | 4 |
| GRN |  | 4 |
| GRPR |  | 4 |
| GSDMD |  | 4 |
| HAUS5 |  | 4 |
| HELLS |  | 4 |
| HIST1H2AK |  | 4 |
| HIST1H4E |  | 4 |
| HSD17B4 |  | 4 |
| HSPBAP1 |  | 4 |
| HSPH1 |  | 4 |
| HTR6 |  | 4 |
| HYAL4 |  | 4 |
| IDH3A |  | 4 |
| IFIT1B |  | 4 |
| IFNA1 |  | 4 |
| IL15 |  | 4 |
| IL1R2 |  | 4 |
| IPPK |  | 4 |
| ISLR |  | 4 |
| JAM2 |  | 4 |
| KCNG1 |  | 4 |
| KCNK5 |  | 4 |
| KCNMB3 |  | 4 |
| KIAA0146 |  | 4 |
| KIAA1383 |  | 4 |
| KPNA4 |  | 4 |
| L3MBTL2 |  | 4 |
| LCE1B |  | 4 |
| LCN2 |  | 4 |
| LEAP2 |  | 4 |
| LILRA5 |  | 4 |
| LRIT1 |  | 4 |
| LRRC39 |  | 4 |
| LRRN4CL |  | 4 |
| LUC7L |  | 4 |
| LYPD1 |  | 4 |
| LYRM2 |  | 4 |
| MAGEB4 |  | 4 |
| MAN1B1 |  | 4 |
| MAP1LC3A |  | 4 |
| MAP3K9 |  | 4 |
| MARC1 |  | 4 |
| MERTK |  | 4 |
| METAP2 |  | 4 |
| METTL18 |  | 4 |
| MFSD4 |  | 4 |
| MGRN1 |  | 4 |
| MITF |  | 4 |
| MLF1 |  | 4 |
| MPHOSPH6 |  | 4 |
| MRPL17 |  | 4 |
| MSH4 |  | 4 |
| NCEH1 |  | 4 |
| NCR2 |  | 4 |
| NDUFV1 |  | 4 |
| NHLRC4 |  | 4 |
| NOX5 |  | 4 |
| NPR2 |  | 4 |
| NPRL3 |  | 4 |
| NUP43 |  | 4 |
| OCLM |  | 4 |
| OCM |  | 4 |
| OPCML |  | 4 |
| OR10T2 |  | 4 |
| OR2C3 |  | 4 |
| OR4D2 |  | 4 |
| OR51Q1 |  | 4 |
| OR52A5 |  | 4 |
| OR5P2 |  | 4 |
| OSR2 |  | 4 |
| OTUD6B |  | 4 |
| OVOL1 |  | 4 |
| PALM2 |  | 4 |
| PANK2 |  | 4 |
| PCDHGA2 |  | 4 |
| PCP2 |  | 4 |
| PDCD10 |  | 4 |
| PDGFD |  | 4 |
| PDHA1 |  | 4 |
| PFKFB3 |  | 4 |
| PGM2L1 |  | 4 |
| PHF11 |  | 4 |
| PHF5A |  | 4 |
| PITX1 |  | 4 |
| PMPCB |  | 4 |
| PPA2 |  | 4 |
| PPM1M |  | 4 |
| PPTC7 |  | 4 |
| PRELID1 |  | 4 |
| PSMB2 |  | 4 |
| PSMB3 |  | 4 |
| PSMB9 |  | 4 |
| PSME3 |  | 4 |
| PTCD1 |  | 4 |
| PTCD2 |  | 4 |
| PTER |  | 4 |
| PTPN12 |  | 4 |
| PTPN6 |  | 4 |
| PTPRK |  | 4 |
| PTPRN2 |  | 4 |
| PVRL2 |  | 4 |
| RAB20 |  | 4 |
| RAET1L |  | 4 |
| RARRES3 |  | 4 |
| RASL12 |  | 4 |
| RCHY1 |  | 4 |
| RETN |  | 4 |
| RFWD3 |  | 4 |
| RGS7 |  | 4 |
| RILP |  | 4 |
| RNASEH1 |  | 4 |
| RNF10 |  | 4 |
| RNFT1 |  | 4 |
| RNPC3 |  | 4 |
| RPL11 |  | 4 |
| RPL18 |  | 4 |
| RPL3 |  | 4 |
| RPP25 |  | 4 |
| RPS6KA4 |  | 4 |
| RPUSD3 |  | 4 |
| RRAGB |  | 4 |
| RRAGD |  | 4 |
| RS1 |  | 4 |
| RTN3 |  | 4 |
| S1PR4 |  | 4 |
| SCOC |  | 4 |
| SCRN1 |  | 4 |
| SCRN2 |  | 4 |
| SEC61A2 |  | 4 |
| SEMG1 |  | 4 |
| SERINC4 |  | 4 |
| SERPINE2 |  | 4 |
| SETD6 |  | 4 |
| SLC22A2 |  | 4 |
| SLC25A48 |  | 4 |
| SLC30A1 |  | 4 |
| SMAD9 |  | 4 |
| SMNDC1 |  | 4 |
| SMR3A |  | 4 |
| SMYD3 |  | 4 |
| SNRPG |  | 4 |
| SPANXF1 |  | 4 |
| SPATA25 |  | 4 |
| SPATA5L1 |  | 4 |
| SPIN3 |  | 4 |
| SPIRE1 |  | 4 |
| SPRYD4 |  | 4 |
| SRPRB |  | 4 |
| SSX1 |  | 4 |
| SSX2 |  | 4 |
| ST8SIA2 |  | 4 |
| STARD8 |  | 4 |
| STAT3 |  | 4 |
| STEAP1B |  | 4 |
| STIM2 |  | 4 |
| STS |  | 4 |
| SULF2 |  | 4 |
| SURF1 |  | 4 |
| SUV39H1 |  | 4 |
| SYT2 |  | 4 |
| TAF6 |  | 4 |
| TAS2R60 |  | 4 |
| TCHP |  | 4 |
| TMEM121 |  | 4 |
| TMEM135 |  | 4 |
| TMEM185A |  | 4 |
| TMEM231 |  | 4 |
| TMIE |  | 4 |
| TNFAIP2 |  | 4 |
| TNNI3K |  | 4 |
| TPM4 |  | 4 |
| TRIM72 |  | 4 |
| TRPT1 |  | 4 |
| TSPAN8 |  | 4 |
| TSSK4 |  | 4 |
| TTC30A |  | 4 |
| TTLL13 |  | 4 |
| TUBGCP5 |  | 4 |
| UBE2U |  | 4 |
| UQCRQ |  | 4 |
| USP22 |  | 4 |
| VCX3A |  | 4 |
| VPS37D |  | 4 |
| WBSCR28 |  | 4 |
| WDR90 |  | 4 |
| WFDC12 |  | 4 |
| YJEFN3 |  | 4 |
| YWHAB |  | 4 |
| ZIK1 |  | 4 |
| ZNF302 |  | 4 |
| ZNF345 |  | 4 |
| ZNF382 |  | 4 |
| ZNF485 |  | 4 |
| ZNF502 |  | 4 |
| ZNF547 |  | 4 |
| ZNF610 |  | 4 |
| ZNF643 |  | 4 |
| ZNF645 |  | 4 |
| ZNF701 |  | 4 |
| ZNRF4 |  | 4 |
| ZSCAN10 |  | 4 |
| ABCD4 |  | 5 |
| ABHD2 |  | 5 |
| ACER2 |  | 5 |
| ADM2 |  | 5 |
| ADPRHL1 |  | 5 |
| AHDC1 |  | 5 |
| ANKRD29 |  | 5 |
| ANKRD55 |  | 5 |
| ANO4 |  | 5 |
| APOM |  | 5 |
| ARL2 |  | 5 |
| ASIP |  | 5 |
| ATP2C2 |  | 5 |
| ATP5H |  | 5 |
| ATP6V0E1 |  | 5 |
| B3GALT1 |  | 5 |
| BCL7C |  | 5 |
| BIVM |  | 5 |
| BMI1 |  | 5 |
| BMPR2 |  | 5 |
| BRIP1 |  | 5 |
| BTBD6 |  | 5 |
| BTNL2 |  | 5 |
| C16orf46 |  | 5 |
| C5orf34 |  | 5 |
| C6orf70 |  | 5 |
| C9orf153 |  | 5 |
| CACNG8 |  | 5 |
| CALD1 |  | 5 |
| CCDC19 |  | 5 |
| CCDC50 |  | 5 |
| CCDC74A |  | 5 |
| CCNI2 |  | 5 |
| CDH8 |  | 5 |
| CDK2AP1 |  | 5 |
| CDK5R1 |  | 5 |
| CERKL |  | 5 |
| CHGB |  | 5 |
| CNP |  | 5 |
| COX7C |  | 5 |
| CPB2 |  | 5 |
| CPNE4 |  | 5 |
| CPT1C |  | 5 |
| CREG2 |  | 5 |
| CRELD1 |  | 5 |
| CSF3 |  | 5 |
| CST2 |  | 5 |
| CSTL1 |  | 5 |
| CXCR4 |  | 5 |
| CYB5R1 |  | 5 |
| CYS1 |  | 5 |
| DAOA |  | 5 |
| DDX28 |  | 5 |
| DEFB114 |  | 5 |
| DEFB129 |  | 5 |
| DIRC1 |  | 5 |
| DNAJC5 |  | 5 |
| DPAGT1 |  | 5 |
| DPH5 |  | 5 |
| DPYSL2 |  | 5 |
| DUOXA2 |  | 5 |
| EFCAB4B |  | 5 |
| EFEMP1 |  | 5 |
| EFHC1 |  | 5 |
| EIF2D |  | 5 |
| EPB41L4A |  | 5 |
| EPB41L5 |  | 5 |
| EPM2A |  | 5 |
| ERCC8 |  | 5 |
| ERI2 |  | 5 |
| ESCO1 |  | 5 |
| ETNK1 |  | 5 |
| FABP7 |  | 5 |
| FAM71F1 |  | 5 |
| FAM9B |  | 5 |
| FANCE |  | 5 |
| FBXO17 |  | 5 |
| FDCSP |  | 5 |
| FGF17 |  | 5 |
| FGFR1OP2 |  | 5 |
| FGG |  | 5 |
| FLT4 |  | 5 |
| GATC |  | 5 |
| GCNT2 |  | 5 |
| GCSH |  | 5 |
| GKAP1 |  | 5 |
| GMEB1 |  | 5 |
| GNA15 |  | 5 |
| GNPTAB |  | 5 |
| GPR3 |  | 5 |
| GPR89B |  | 5 |
| GRIA1 |  | 5 |
| GTF2A1L |  | 5 |
| HAPLN3 |  | 5 |
| HAUS7 |  | 5 |
| HERPUD2 |  | 5 |
| HIST1H2AJ |  | 5 |
| HIST1H2BF |  | 5 |
| HIST1H2BN |  | 5 |
| HMBOX1 |  | 5 |
| HMGB3 |  | 5 |
| HN1 |  | 5 |
| HOOK3 |  | 5 |
| HOXA6 |  | 5 |
| HRASLS5 |  | 5 |
| HSD17B3 |  | 5 |
| HSD17B8 |  | 5 |
| HSH2D |  | 5 |
| HSPB9 |  | 5 |
| IDH2 |  | 5 |
| IGF1 |  | 5 |
| IHH |  | 5 |
| IL9R |  | 5 |
| IMMP1L |  | 5 |
| ISM2 |  | 5 |
| KCNS3 |  | 5 |
| KIF12 |  | 5 |
| KRTAP5-1 |  | 5 |
| LDLRAP1 |  | 5 |
| LINS |  | 5 |
| LPAR6 |  | 5 |
| LRCH1 |  | 5 |
| LRCH3 |  | 5 |
| LRRC41 |  | 5 |
| LRRTM3 |  | 5 |
| LST1 |  | 5 |
| MAGT1 |  | 5 |
| MED25 |  | 5 |
| MGST3 |  | 5 |
| MILR1 |  | 5 |
| MON1A |  | 5 |
| MPP2 |  | 5 |
| MREG |  | 5 |
| MS4A13 |  | 5 |
| NEK10 |  | 5 |
| NELF |  | 5 |
| NHLRC2 |  | 5 |
| NPIP |  | 5 |
| NSDHL |  | 5 |
| NUAK1 |  | 5 |
| OPN5 |  | 5 |
| OR11A1 |  | 5 |
| OR2A2 |  | 5 |
| OR4F16 |  | 5 |
| OR51B5 |  | 5 |
| OR56B1 |  | 5 |
| OR5M8 |  | 5 |
| P2RY4 |  | 5 |
| PAMR1 |  | 5 |
| PAQR5 |  | 5 |
| PDCD6IP |  | 5 |
| PDGFRB |  | 5 |
| PDIA3 |  | 5 |
| PDK2 |  | 5 |
| PEX11A |  | 5 |
| PIEZO1 |  | 5 |
| PLAG1 |  | 5 |
| PLK1 |  | 5 |
| PNN |  | 5 |
| PODXL |  | 5 |
| POLR2D |  | 5 |
| PRELP |  | 5 |
| RAB11FIP5 |  | 5 |
| RBM43 |  | 5 |
| RBM5 |  | 5 |
| RBMS2 |  | 5 |
| RFC1 |  | 5 |
| RFFL |  | 5 |
| RFT1 |  | 5 |
| RHBDD2 |  | 5 |
| RIPK3 |  | 5 |
| RNASE6 |  | 5 |
| RNF214 |  | 5 |
| RNF32 |  | 5 |
| RNF5 |  | 5 |
| RPS16 |  | 5 |
| SCRG1 |  | 5 |
| SDF2 |  | 5 |
| SEC61A1 |  | 5 |
| SELPLG |  | 5 |
| SERINC2 |  | 5 |
| SERINC3 |  | 5 |
| SERTM1 |  | 5 |
| SH3BGRL |  | 5 |
| SHH |  | 5 |
| SLC1A7 |  | 5 |
| SLC25A51 |  | 5 |
| SLC29A1 |  | 5 |
| SLC2A10 |  | 5 |
| SLC6A20 |  | 5 |
| SLC6A5 |  | 5 |
| SLCO6A1 |  | 5 |
| SPTY2D1 |  | 5 |
| ST3GAL4 |  | 5 |
| STXBP5L |  | 5 |
| SYMPK |  | 5 |
| SYNE2 |  | 5 |
| TBX18 |  | 5 |
| TDO2 |  | 5 |
| TEKT4 |  | 5 |
| TLX3 |  | 5 |
| TMEM155 |  | 5 |
| TMEM182 |  | 5 |
| TMEM200A |  | 5 |
| TMEM30B |  | 5 |
| TMEM41B |  | 5 |
| TMEM63A |  | 5 |
| TRAPPC2P1 |  | 5 |
| TRIM67 |  | 5 |
| TRIM74 |  | 5 |
| TRMT1L |  | 5 |
| TST |  | 5 |
| TULP2 |  | 5 |
| TXNDC2 |  | 5 |
| UCP2 |  | 5 |
| UTP23 |  | 5 |
| VIT |  | 5 |
| VMA21 |  | 5 |
| VPS36 |  | 5 |
| WDR60 |  | 5 |
| WNT2 |  | 5 |
| XCR1 |  | 5 |
| ZBTB24 |  | 5 |
| ZC3H8 |  | 5 |
| ZCCHC9 |  | 5 |
| ZMAT3 |  | 5 |
| ZNF174 |  | 5 |
| ZNF211 |  | 5 |
| ZNF264 |  | 5 |
| ZNF34 |  | 5 |
| ZNF467 |  | 5 |
| ZNF532 |  | 5 |
| ZNF593 |  | 5 |
| ZNF766 |  | 5 |
| ZNF804B |  | 5 |
| ZNHIT1 |  | 5 |
| ZNHIT3 |  | 5 |
| ZSCAN1 |  | 5 |
| ZWINT |  | 5 |
| SH2B3 |  | 7 |
| ACSF3 |  | 7 |
| ADORA2B |  | 7 |
| AKAP2 |  | 7 |
| ALG2 |  | 7 |
| ALG3 |  | 7 |
| AMHR2 |  | 7 |
| ANKRD30A |  | 7 |
| AP4E1 |  | 7 |
| APBA3 |  | 7 |
| APITD1 |  | 7 |
| ARGLU1 |  | 7 |
| ATG3 |  | 7 |
| B3GALT4 |  | 7 |
| BAMBI |  | 7 |
| BANK1 |  | 7 |
| C10orf47 |  | 7 |
| C10orf54 |  | 7 |
| C14orf21 |  | 7 |
| C15orf27 |  | 7 |
| C15orf32 |  | 7 |
| C19orf75 |  | 7 |
| C20orf118 |  | 7 |
| C2orf29 |  | 7 |
| C6orf226 |  | 7 |
| CABP1 |  | 7 |
| CACNG4 |  | 7 |
| CAMK1G |  | 7 |
| CAMKV |  | 7 |
| CAPN9 |  | 7 |
| CAV3 |  | 7 |
| CCBP2 |  | 7 |
| CCDC134 |  | 7 |
| CCDC148 |  | 7 |
| CCDC88C |  | 7 |
| CCNJ |  | 7 |
| CD24 |  | 7 |
| CDC23 |  | 7 |
| CDYL |  | 7 |
| CEACAM7 |  | 7 |
| CELA2B |  | 7 |
| CEP112 |  | 7 |
| CHMP4A |  | 7 |
| CLASP1 |  | 7 |
| CNPY3 |  | 7 |
| COPZ1 |  | 7 |
| CRY1 |  | 7 |
| CYP4Z1 |  | 7 |
| DAPP1 |  | 7 |
| DAXX |  | 7 |
| DCAF6 |  | 7 |
| DDR1 |  | 7 |
| DEF8 |  | 7 |
| DENND2D |  | 7 |
| DIAPH3 |  | 7 |
| DIS3L |  | 7 |
| DNAJB11 |  | 7 |
| DNAJB7 |  | 7 |
| DNALI1 |  | 7 |
| DR1 |  | 7 |
| DSCR4 |  | 7 |
| EHBP1 |  | 7 |
| ENTPD6 |  | 7 |
| FAM176A |  | 7 |
| FAM195B |  | 7 |
| FAM19A4 |  | 7 |
| FBXL18 |  | 7 |
| FRMD1 |  | 7 |
| GAGE10 |  | 7 |
| GDAP1L1 |  | 7 |
| GDNF |  | 7 |
| GEMIN2 |  | 7 |
| GFI1 |  | 7 |
| GIMAP2 |  | 7 |
| GINS1 |  | 7 |
| GNG4 |  | 7 |
| GNPDA2 |  | 7 |
| GPA33 |  | 7 |
| GPR176 |  | 7 |
| GPR39 |  | 7 |
| GPR83 |  | 7 |
| GPR84 |  | 7 |
| GTF2E2 |  | 7 |
| GTF2H1 |  | 7 |
| HDAC1 |  | 7 |
| HHIPL2 |  | 7 |
| HIST4H4 |  | 7 |
| HLA-DOB |  | 7 |
| HNRNPF |  | 7 |
| HOXB1 |  | 7 |
| HS3ST4 |  | 7 |
| HS6ST3 |  | 7 |
| HSD17B10 |  | 7 |
| HTR2B |  | 7 |
| ICOS |  | 7 |
| IFIT2 |  | 7 |
| IFLTD1 |  | 7 |
| IGFBP4 |  | 7 |
| IGSF5 |  | 7 |
| IL26 |  | 7 |
| IL36A |  | 7 |
| IQCF2 |  | 7 |
| ITGA2 |  | 7 |
| KIAA1522 |  | 7 |
| KIR2DL4 |  | 7 |
| KLF7 |  | 7 |
| KLHDC7B |  | 7 |
| KRT78 |  | 7 |
| KXD1 |  | 7 |
| LCOR |  | 7 |
| LCP2 |  | 7 |
| LENG1 |  | 7 |
| LHX3 |  | 7 |
| LIPA |  | 7 |
| LMO2 |  | 7 |
| LPAR1 |  | 7 |
| LYRM4 |  | 7 |
| MAP7D2 |  | 7 |
| MARK4 |  | 7 |
| MCMDC2 |  | 7 |
| MFSD2A |  | 7 |
| MIIP |  | 7 |
| MMP10 |  | 7 |
| MOGAT3 |  | 7 |
| MRPL44 |  | 7 |
| MRPS36 |  | 7 |
| MS4A7 |  | 7 |
| MYH9 |  | 7 |
| MYO5C |  | 7 |
| NAALADL1 |  | 7 |
| NDUFA9 |  | 7 |
| NEBL |  | 7 |
| NEK8 |  | 7 |
| NKIRAS2 |  | 7 |
| NMUR2 |  | 7 |
| NUDT6 |  | 7 |
| NUDT9 |  | 7 |
| ODF2 |  | 7 |
| OMD |  | 7 |
| OPTC |  | 7 |
| OR2D3 |  | 7 |
| OR2L13 |  | 7 |
| OR51B2 |  | 7 |
| OR6A2 |  | 7 |
| PARP3 |  | 7 |
| PAX4 |  | 7 |
| PDGFRA |  | 7 |
| PEX12 |  | 7 |
| PGAM1 |  | 7 |
| PIK3CB |  | 7 |
| PLCL2 |  | 7 |
| PNMA6D |  | 7 |
| POLL |  | 7 |
| PPAPDC1A |  | 7 |
| PRKCSH |  | 7 |
| PSMD9 |  | 7 |
| PSMF1 |  | 7 |
| PSMG1 |  | 7 |
| PTPRA |  | 7 |
| PUS7 |  | 7 |
| RAB11FIP2 |  | 7 |
| RAC1 |  | 7 |
| RBBP5 |  | 7 |
| RBM18 |  | 7 |
| REEP3 |  | 7 |
| RELA |  | 7 |
| RGS9 |  | 7 |
| RNF113A |  | 7 |
| RPAP3 |  | 7 |
| RPL7L1 |  | 7 |
| RPS6KA6 |  | 7 |
| SERPINA10 |  | 7 |
| SH3BP4 |  | 7 |
| SIGLEC5 |  | 7 |
| SLC10A4 |  | 7 |
| SLC10A6 |  | 7 |
| SLC22A11 |  | 7 |
| SLC25A18 |  | 7 |
| SLC25A36 |  | 7 |
| SLC25A42 |  | 7 |
| SLC26A9 |  | 7 |
| SLC27A6 |  | 7 |
| SLC2A5 |  | 7 |
| SLC46A3 |  | 7 |
| SLC6A11 |  | 7 |
| SLC7A10 |  | 7 |
| SLC9A8 |  | 7 |
| SP100 |  | 7 |
| SQSTM1 |  | 7 |
| ST13 |  | 7 |
| STAP1 |  | 7 |
| STAT4 |  | 7 |
| STK11 |  | 7 |
| STRA6 |  | 7 |
| SUN3 |  | 7 |
| TAS2R43 |  | 7 |
| TBK1 |  | 7 |
| TBX15 |  | 7 |
| TCEANC2 |  | 7 |
| TEX10 |  | 7 |
| TEX2 |  | 7 |
| TFB1M |  | 7 |
| TIAF1 |  | 7 |
| TTYH1 |  | 7 |
| UCN2 |  | 7 |
| UFD1L |  | 7 |
| VIPR1 |  | 7 |
| WDR49 |  | 7 |
| WNT7B |  | 7 |
| XXYLT1 |  | 7 |
| ZBED5 |  | 7 |
| ZBTB47 |  | 7 |
| ZDHHC17 |  | 7 |
| ZFAND4 |  | 7 |
| ZNF121 |  | 7 |
| ZNF146 |  | 7 |
| ZNF26 |  | 7 |
| ZNF295 |  | 7 |
| ZNF320 |  | 7 |
| ZNF519 |  | 7 |
| ZNF713 |  | 7 |
| ZNF776 |  | 7 |
| ZPLD1 |  | 7 |
| ADAM33 |  | 8 |
| AIRE |  | 8 |
| ALOX5AP |  | 8 |
| ASGR2 |  | 8 |
| ASTN2 |  | 8 |
| ATP6V1C2 |  | 8 |
| AUP1 |  | 8 |
| AVIL |  | 8 |
| AVPI1 |  | 8 |
| BFSP2 |  | 8 |
| BHLHA15 |  | 8 |
| C17orf105 |  | 8 |
| C17orf64 |  | 8 |
| C19orf70 |  | 8 |
| C1orf122 |  | 8 |
| C1QC |  | 8 |
| C1QL4 |  | 8 |
| C3orf58 |  | 8 |
| C3orf62 |  | 8 |
| C5AR1 |  | 8 |
| C6orf203 |  | 8 |
| C6orf222 |  | 8 |
| C8orf33 |  | 8 |
| C9orf85 |  | 8 |
| CAPN6 |  | 8 |
| CASD1 |  | 8 |
| CCDC140 |  | 8 |
| CCDC33 |  | 8 |
| CCDC58 |  | 8 |
| CCNYL1 |  | 8 |
| CENPL |  | 8 |
| CIRH1A |  | 8 |
| CMA1 |  | 8 |
| COL10A1 |  | 8 |
| COQ2 |  | 8 |
| COX6A2 |  | 8 |
| CSGALNACT1 |  | 8 |
| CSNK1E |  | 8 |
| CSNK1G3 |  | 8 |
| CTGF |  | 8 |
| CYB5R4 |  | 8 |
| CYP2S1 |  | 8 |
| DDOST |  | 8 |
| DDX55 |  | 8 |
| DDX59 |  | 8 |
| DNAJC3 |  | 8 |
| DNAJC7 |  | 8 |
| DNTTIP2 |  | 8 |
| EP400 |  | 8 |
| EXOC3L2 |  | 8 |
| FAM111B |  | 8 |
| FAM222B |  | 8 |
| FARSB |  | 8 |
| FBXO28 |  | 8 |
| FKBP14 |  | 8 |
| FSHB |  | 8 |
| FTH1 |  | 8 |
| GAL3ST2 |  | 8 |
| GALNT9 |  | 8 |
| GAS2 |  | 8 |
| GAST |  | 8 |
| GBP1 |  | 8 |
| GDPD1 |  | 8 |
| GNAZ |  | 8 |
| GRB2 |  | 8 |
| GUK1 |  | 8 |
| H1FOO |  | 8 |
| HEMGN |  | 8 |
| HEYL |  | 8 |
| HLA-DRB3 |  | 8 |
| HLX |  | 8 |
| HRH1 |  | 8 |
| HTR1D |  | 8 |
| ID3 |  | 8 |
| IFT57 |  | 8 |
| IL20RA |  | 8 |
| INHBA |  | 8 |
| INPP5J |  | 8 |
| KCNA5 |  | 8 |
| KCNAB1 |  | 8 |
| KIF23 |  | 8 |
| KLK1 |  | 8 |
| KLK8 |  | 8 |
| LCE3C |  | 8 |
| LENEP |  | 8 |
| LHX6 |  | 8 |
| LRRCC1 |  | 8 |
| MANF |  | 8 |
| MAPK1 |  | 8 |
| MCCD1 |  | 8 |
| MCM9 |  | 8 |
| METTL6 |  | 8 |
| MOCS3 |  | 8 |
| MOG |  | 8 |
| MPP5 |  | 8 |
| MPPE1 |  | 8 |
| MTERFD1 |  | 8 |
| NDUFS3 |  | 8 |
| NEDD1 |  | 8 |
| NOL9 |  | 8 |
| NPBWR2 |  | 8 |
| NPM1 |  | 8 |
| NPS |  | 8 |
| NUMB |  | 8 |
| O3FAR1 |  | 8 |
| OPN4 |  | 8 |
| OR14J1 |  | 8 |
| OR4D6 |  | 8 |
| OSBPL7 |  | 8 |
| OTOP2 |  | 8 |
| PCDHB7 |  | 8 |
| PDCL3 |  | 8 |
| PFKL |  | 8 |
| PHF8 |  | 8 |
| PIK3CD |  | 8 |
| PLA2G2E |  | 8 |
| PLCG2 |  | 8 |
| POP7 |  | 8 |
| POPDC3 |  | 8 |
| POTEG |  | 8 |
| PPYR1 |  | 8 |
| PTH2 |  | 8 |
| RAB4A |  | 8 |
| RASEF |  | 8 |
| RCVRN |  | 8 |
| RNF4 |  | 8 |
| RNMTL1 |  | 8 |
| RPAIN |  | 8 |
| RPL10 |  | 8 |
| SARS2 |  | 8 |
| SCG5 |  | 8 |
| SERPINF2 |  | 8 |
| SGPL1 |  | 8 |
| SHPK |  | 8 |
| SKOR1 |  | 8 |
| SLAIN2 |  | 8 |
| SLAMF1 |  | 8 |
| SLC17A2 |  | 8 |
| SLC25A21 |  | 8 |
| SLC25A22 |  | 8 |
| SLC25A32 |  | 8 |
| SLC39A5 |  | 8 |
| SLC5A8 |  | 8 |
| SLC9A5 |  | 8 |
| SLFN5 |  | 8 |
| SMARCAL1 |  | 8 |
| SPARC |  | 8 |
| SRMS |  | 8 |
| SULT1C3 |  | 8 |
| SYPL2 |  | 8 |
| SYTL3 |  | 8 |
| TBCK |  | 8 |
| TCTN1 |  | 8 |
| TEKT2 |  | 8 |
| TIMM50 |  | 8 |
| TMEM110 |  | 8 |
| TMEM14A |  | 8 |
| TMEM204 |  | 8 |
| TMEM9 |  | 8 |
| TMEM98 |  | 8 |
| TMUB1 |  | 8 |
| TNPO1 |  | 8 |
| TNRC6A |  | 8 |
| TPD52L3 |  | 8 |
| TPH2 |  | 8 |
| TRHR |  | 8 |
| TRIP4 |  | 8 |
| TRMT1 |  | 8 |
| TSEN2 |  | 8 |
| TSSC4 |  | 8 |
| UMPS |  | 8 |
| UPK2 |  | 8 |
| VARS |  | 8 |
| WDR72 |  | 8 |
| WIPI1 |  | 8 |
| XAGE3 |  | 8 |
| ZDHHC16 |  | 8 |
| ZFAND2A |  | 8 |
| ZFP64 |  | 8 |
| ZNF134 |  | 8 |
| ZNF253 |  | 8 |
| ZNF256 |  | 8 |
| ZNF484 |  | 8 |
| ZNF512 |  | 8 |
| ZNF571 |  | 8 |
| ZNF705D |  | 8 |
| ZNF85 |  | 8 |
| ABI3BP |  | 9 |
| ALG10B |  | 9 |
| ARHGEF10 |  | 9 |
| ARNT2 |  | 9 |
| BCAM |  | 9 |
| BECN1 |  | 9 |
| C10orf116 |  | 9 |
| C10orf67 |  | 9 |
| C12orf32 |  | 9 |
| C19orf66 |  | 9 |
| C1orf131 |  | 9 |
| C20orf111 |  | 9 |
| C9orf116 |  | 9 |
| C9orf78 |  | 9 |
| CAPN8 |  | 9 |
| CCDC114 |  | 9 |
| CCDC27 |  | 9 |
| CCR10 |  | 9 |
| CEP41 |  | 9 |
| CHRM4 |  | 9 |
| CLIC1 |  | 9 |
| CNKSR3 |  | 9 |
| CST6 |  | 9 |
| CTPS2 |  | 9 |
| DBR1 |  | 9 |
| DCTN5 |  | 9 |
| DEFB105B |  | 9 |
| DNAJC25 |  | 9 |
| DPEP1 |  | 9 |
| ERO1L |  | 9 |
| EXOSC7 |  | 9 |
| FAM113B |  | 9 |
| FAM167A |  | 9 |
| FAM170A |  | 9 |
| FAM187B |  | 9 |
| FAM60A |  | 9 |
| FAM82A2 |  | 9 |
| FERD3L |  | 9 |
| GMPR |  | 9 |
| GPR173 |  | 9 |
| GPR18 |  | 9 |
| GPR63 |  | 9 |
| HDAC8 |  | 9 |
| HOXA7 |  | 9 |
| IGFBPL1 |  | 9 |
| IKZF2 |  | 9 |
| IL1RAP |  | 9 |
| JAK2 |  | 9 |
| KRT34 |  | 9 |
| LALBA |  | 9 |
| LIMD2 |  | 9 |
| M6PR |  | 9 |
| MAPK1IP1L |  | 9 |
| MEIS2 |  | 9 |
| MMP14 |  | 9 |
| MOB3B |  | 9 |
| MORC1 |  | 9 |
| MRPL11 |  | 9 |
| MRPS7 |  | 9 |
| MS4A12 |  | 9 |
| N6AMT2 |  | 9 |
| NR1D2 |  | 9 |
| OLFM4 |  | 9 |
| OPA1 |  | 9 |
| OR51V1 |  | 9 |
| OR56B4 |  | 9 |
| PAEP |  | 9 |
| PARM1 |  | 9 |
| PCDHA10 |  | 9 |
| PCDHAC2 |  | 9 |
| PIAS1 |  | 9 |
| PIP4K2B |  | 9 |
| PITPNM3 |  | 9 |
| PKN3 |  | 9 |
| PNPLA3 |  | 9 |
| PPAN |  | 9 |
| PPP1R16B |  | 9 |
| PROK1 |  | 9 |
| PSD3 |  | 9 |
| PSMA7 |  | 9 |
| PSMD2 |  | 9 |
| RBM11 |  | 9 |
| RCN3 |  | 9 |
| RIMS2 |  | 9 |
| RNMT |  | 9 |
| RTDR1 |  | 9 |
| RUSC1 |  | 9 |
| SCGB1D1 |  | 9 |
| SEMA4A |  | 9 |
| SEMG2 |  | 9 |
| SERPINB8 |  | 9 |
| SHISA2 |  | 9 |
| SKAP1 |  | 9 |
| SLC13A1 |  | 9 |
| SLC22A16 |  | 9 |
| SMCR7 |  | 9 |
| SNAP23 |  | 9 |
| SNAP29 |  | 9 |
| SPC24 |  | 9 |
| SPZ1 |  | 9 |
| SRGAP3 |  | 9 |
| SRPK1 |  | 9 |
| TALDO1 |  | 9 |
| TBX20 |  | 9 |
| TCAP |  | 9 |
| TDP1 |  | 9 |
| TEX261 |  | 9 |
| TMEM115 |  | 9 |
| TMEM35 |  | 9 |
| TMEM5 |  | 9 |
| TNFSF12 |  | 9 |
| TOMM34 |  | 9 |
| TPM1 |  | 9 |
| TRIM2 |  | 9 |
| TRMT61A |  | 9 |
| TSHZ1 |  | 9 |
| TSPAN6 |  | 9 |
| TTC16 |  | 9 |
| WDR53 |  | 9 |
| ZFHX2 |  | 9 |
| ZNF238 |  | 9 |
| ZNF281 |  | 9 |
| ZNF550 |  | 9 |
| ZRSR2 |  | 9 |
| ACP2 |  | 11 |
| ACPT |  | 11 |
| ADORA2A |  | 11 |
| ADRA2B |  | 11 |
| AGBL5 |  | 11 |
| AGPAT6 |  | 11 |
| AKD1 |  | 11 |
| AKNAD1 |  | 11 |
| AMBRA1 |  | 11 |
| AMY2A |  | 11 |
| ANKRD32 |  | 11 |
| AP1S2 |  | 11 |
| APOC4 |  | 11 |
| ASCL3 |  | 11 |
| ATP1A1 |  | 11 |
| ATP6V1G2 |  | 11 |
| B4GALT6 |  | 11 |
| BAG6 |  | 11 |
| BTF3 |  | 11 |
| C14orf105 |  | 11 |
| C1orf123 |  | 11 |
| C1orf65 |  | 11 |
| C20orf141 |  | 11 |
| C20orf173 |  | 11 |
| C21orf77 |  | 11 |
| CAMP |  | 11 |
| CCDC74B |  | 11 |
| CCND2 |  | 11 |
| CDY2A |  | 11 |
| CLDN8 |  | 11 |
| CPLX4 |  | 11 |
| CXADR |  | 11 |
| CYP2A7 |  | 11 |
| DEFB127 |  | 11 |
| DST |  | 11 |
| EBP |  | 11 |
| EBPL |  | 11 |
| EEF1A2 |  | 11 |
| EFCAB4A |  | 11 |
| EIF2B2 |  | 11 |
| EIF3M |  | 11 |
| EMB |  | 11 |
| ERO1LB |  | 11 |
| EXOSC5 |  | 11 |
| EYA3 |  | 11 |
| FFAR3 |  | 11 |
| FGF6 |  | 11 |
| FKRP |  | 11 |
| FMN1 |  | 11 |
| GABRQ |  | 11 |
| GBP6 |  | 11 |
| GMPPB |  | 11 |
| GPR183 |  | 11 |
| HCFC2 |  | 11 |
| HFE2 |  | 11 |
| HIST1H2BH |  | 11 |
| HRH2 |  | 11 |
| IGFL1 |  | 11 |
| IGSF8 |  | 11 |
| IRAK2 |  | 11 |
| KAT5 |  | 11 |
| KIAA0895 |  | 11 |
| KIF6 |  | 11 |
| KLHL14 |  | 11 |
| LHFP |  | 11 |
| LRRC19 |  | 11 |
| LY6H |  | 11 |
| METTL21C |  | 11 |
| MFAP5 |  | 11 |
| MMP25 |  | 11 |
| MUSTN1 |  | 11 |
| NXF3 |  | 11 |
| OR2W1 |  | 11 |
| OR8A1 |  | 11 |
| OR8D4 |  | 11 |
| OR8I2 |  | 11 |
| PBX3 |  | 11 |
| PCGF6 |  | 11 |
| PID1 |  | 11 |
| PPM1A |  | 11 |
| PPP1R18 |  | 11 |
| PQLC2 |  | 11 |
| PRR5 |  | 11 |
| PTPN22 |  | 11 |
| PYCR2 |  | 11 |
| RABGGTA |  | 11 |
| RAPGEF4 |  | 11 |
| REEP1 |  | 11 |
| RIBC2 |  | 11 |
| RIMKLB |  | 11 |
| RPL37 |  | 11 |
| RPS27L |  | 11 |
| RPS6KB1 |  | 11 |
| S100A4 |  | 11 |
| SDC4 |  | 11 |
| SHISA4 |  | 11 |
| SLC18A2 |  | 11 |
| SLC43A3 |  | 11 |
| SPANXB2 |  | 11 |
| SPRED2 |  | 11 |
| SRCIN1 |  | 11 |
| SRSF7 |  | 11 |
| TEX30 |  | 11 |
| TGM7 |  | 11 |
| TM2D2 |  | 11 |
| TMEM72 |  | 11 |
| TMEM9B |  | 11 |
| TPST2 |  | 11 |
| TRIM4 |  | 11 |
| TRPC1 |  | 11 |
| TSPYL1 |  | 11 |
| UBL3 |  | 11 |
| USE1 |  | 11 |
| VSIG2 |  | 11 |
| WFDC1 |  | 11 |
| WWC3 |  | 11 |
| WWP2 |  | 11 |
| YIF1B |  | 11 |
| ZCCHC24 |  | 11 |
| ZNF202 |  | 11 |
| ZNF37A |  | 11 |
| ZNF397 |  | 11 |
| ZNF620 |  | 11 |
| ZNF660 |  | 11 |
| ZNF830 |  | 11 |
| ZSCAN29 |  | 11 |
| ABAT |  | 12 |
| AFF4 |  | 12 |
| AGBL3 |  | 12 |
| AMACR |  | 12 |
| ANKAR |  | 12 |
| APOBEC3B |  | 12 |
| ARRDC1 |  | 12 |
| AVP |  | 12 |
| BOLA3 |  | 12 |
| C16orf58 |  | 12 |
| C1orf150 |  | 12 |
| C2orf44 |  | 12 |
| C7 |  | 12 |
| CCDC132 |  | 12 |
| CD300E |  | 12 |
| CDKL3 |  | 12 |
| CDRT1 |  | 12 |
| CFLAR |  | 12 |
| CISD1 |  | 12 |
| CLCA1 |  | 12 |
| CLEC1B |  | 12 |
| CUEDC1 |  | 12 |
| DCD |  | 12 |
| DLL1 |  | 12 |
| EGFL8 |  | 12 |
| EMC3 |  | 12 |
| FAM103A1 |  | 12 |
| FAM117B |  | 12 |
| FAM124A |  | 12 |
| FANCD2 |  | 12 |
| FASTKD2 |  | 12 |
| FBXO4 |  | 12 |
| FGA |  | 12 |
| FRA10AC1 |  | 12 |
| GALNTL1 |  | 12 |
| GAS2L2 |  | 12 |
| GPBP1 |  | 12 |
| GPS1 |  | 12 |
| HELB |  | 12 |
| HMGCLL1 |  | 12 |
| HRH4 |  | 12 |
| IFI35 |  | 12 |
| IL37 |  | 12 |
| JAM3 |  | 12 |
| KLHL34 |  | 12 |
| KRT6B |  | 12 |
| KRTAP19-4 |  | 12 |
| LARP1 |  | 12 |
| LCE3B |  | 12 |
| LCE4A |  | 12 |
| LRRC3B |  | 12 |
| MINK1 |  | 12 |
| MKL2 |  | 12 |
| MYLK3 |  | 12 |
| NANOS2 |  | 12 |
| NENF |  | 12 |
| NOSIP |  | 12 |
| NOXO1 |  | 12 |
| ODF1 |  | 12 |
| OR51F2 |  | 12 |
| OR8G5 |  | 12 |
| OSTM1 |  | 12 |
| PCDHGA7 |  | 12 |
| PCDHGB4 |  | 12 |
| PLA2G16 |  | 12 |
| PLD6 |  | 12 |
| PLEKHO2 |  | 12 |
| POLH |  | 12 |
| RAB1A |  | 12 |
| RABGEF1 |  | 12 |
| RHPN1 |  | 12 |
| RNF151 |  | 12 |
| RNF217 |  | 12 |
| RPL39 |  | 12 |
| RSL1D1 |  | 12 |
| SLC22A23 |  | 12 |
| SLC25A43 |  | 12 |
| SLC37A2 |  | 12 |
| SPATA17 |  | 12 |
| SPRR3 |  | 12 |
| ST8SIA4 |  | 12 |
| SYNPR |  | 12 |
| TGFA |  | 12 |
| TMC4 |  | 12 |
| TMEM170A |  | 12 |
| TPST1 |  | 12 |
| TTC1 |  | 12 |
| VOPP1 |  | 12 |
| WNT9A |  | 12 |
| ZC3H12D |  | 12 |
| ZFYVE26 |  | 12 |
| ZNF184 |  | 12 |
| ZP3 |  | 12 |
| ACTR3B |  | 13 |
| ADM |  | 13 |
| AGRP |  | 13 |
| AP5M1 |  | 13 |
| BEND7 |  | 13 |
| C11orf42 |  | 13 |
| C12orf44 |  | 13 |
| C14orf118 |  | 13 |
| C17orf79 |  | 13 |
| C1orf135 |  | 13 |
| CA4 |  | 13 |
| CCDC141 |  | 13 |
| CCNJL |  | 13 |
| CCR2 |  | 13 |
| CEACAM8 |  | 13 |
| CHCHD7 |  | 13 |
| CTCF |  | 13 |
| CTLA4 |  | 13 |
| CXCL16 |  | 13 |
| DTL |  | 13 |
| FAM110D |  | 13 |
| FAM13B |  | 13 |
| FASN |  | 13 |
| FIS1 |  | 13 |
| FKBP9 |  | 13 |
| GFOD1 |  | 13 |
| GUCY2D |  | 13 |
| HAUS1 |  | 13 |
| HHAT |  | 13 |
| HM13 |  | 13 |
| HNRNPU |  | 13 |
| HOXA5 |  | 13 |
| ISCA1 |  | 13 |
| KSR1 |  | 13 |
| LMBR1L |  | 13 |
| MARK2 |  | 13 |
| ME3 |  | 13 |
| MRFAP1 |  | 13 |
| MS4A6E |  | 13 |
| MSS51 |  | 13 |
| NPPB |  | 13 |
| PITX2 |  | 13 |
| PLEKHA3 |  | 13 |
| PODXL2 |  | 13 |
| QRSL1 |  | 13 |
| RCL1 |  | 13 |
| RNF149 |  | 13 |
| RRAD |  | 13 |
| SEC11A |  | 13 |
| SEC23B |  | 13 |
| SERF2 |  | 13 |
| SFR1 |  | 13 |
| SIVA1 |  | 13 |
| SLC19A2 |  | 13 |
| SLC26A2 |  | 13 |
| SLC30A9 |  | 13 |
| SPANXN2 |  | 13 |
| STAT2 |  | 13 |
| STMN1 |  | 13 |
| SUDS3 |  | 13 |
| SYT8 |  | 13 |
| TAB1 |  | 13 |
| TFPI |  | 13 |
| TGM2 |  | 13 |
| TINAGL1 |  | 13 |
| TNFRSF1B |  | 13 |
| TRIM35 |  | 13 |
| TUSC3 |  | 13 |
| ZNF263 |  | 13 |
| ABTB1 |  | 15 |
| ACAD9 |  | 15 |
| ACTR1A |  | 15 |
| ARHGAP15 |  | 15 |
| BAIAP2 |  | 15 |
| C2orf27A |  | 15 |
| C4orf17 |  | 15 |
| C8orf46 |  | 15 |
| CATSPERD |  | 15 |
| CBX7 |  | 15 |
| CCDC69 |  | 15 |
| CCDC85C |  | 15 |
| CD3G |  | 15 |
| CD79A |  | 15 |
| CEP70 |  | 15 |
| CGNL1 |  | 15 |
| CLDN7 |  | 15 |
| CSRNP2 |  | 15 |
| CYP1A2 |  | 15 |
| DCLRE1B |  | 15 |
| DTX3 |  | 15 |
| FAM161B |  | 15 |
| FAM69A |  | 15 |
| FAM82A1 |  | 15 |
| GABBR2 |  | 15 |
| HECTD3 |  | 15 |
| IFI16 |  | 15 |
| INTS9 |  | 15 |
| KLK15 |  | 15 |
| KLK2 |  | 15 |
| LAT |  | 15 |
| LDHAL6B |  | 15 |
| LPCAT3 |  | 15 |
| LYRM5 |  | 15 |
| MARK3 |  | 15 |
| NACA2 |  | 15 |
| OR12D3 |  | 15 |
| OR2T35 |  | 15 |
| OR4D10 |  | 15 |
| OR5H6 |  | 15 |
| OR5T1 |  | 15 |
| OTUB2 |  | 15 |
| PDGFA |  | 15 |
| PDZD4 |  | 15 |
| PGAP3 |  | 15 |
| PIGM |  | 15 |
| PIK3R1 |  | 15 |
| POGZ |  | 15 |
| POLB |  | 15 |
| POU6F2 |  | 15 |
| PRKD2 |  | 15 |
| PRPSAP2 |  | 15 |
| RPS9 |  | 15 |
| SESTD1 |  | 15 |
| SLC35E2 |  | 15 |
| SSTR1 |  | 15 |
| TAS2R38 |  | 15 |
| THADA |  | 15 |
| TMEM43 |  | 15 |
| UBE2R2 |  | 15 |
| UBTD2 |  | 15 |
| ZNF248 |  | 15 |
| ZNF266 |  | 15 |
| ZNF331 |  | 15 |
| ZNF597 |  | 15 |
| ARSE |  | 16 |
| ASCL1 |  | 16 |
| ATP1B4 |  | 16 |
| ATP6V1G1 |  | 16 |
| AVPR1A |  | 16 |
| C15orf26 |  | 16 |
| C16orf88 |  | 16 |
| C9orf24 |  | 16 |
| CCDC22 |  | 16 |
| CDK6 |  | 16 |
| CHAD |  | 16 |
| CHRAC1 |  | 16 |
| CUL4B |  | 16 |
| CYLC1 |  | 16 |
| DLK2 |  | 16 |
| DUX1 |  | 16 |
| ENTPD4 |  | 16 |
| FAM21A |  | 16 |
| FANCG |  | 16 |
| FIBIN |  | 16 |
| GGA1 |  | 16 |
| GLTSCR2 |  | 16 |
| GPR55 |  | 16 |
| HEXA |  | 16 |
| HIST1H4J |  | 16 |
| HIST3H3 |  | 16 |
| IYD |  | 16 |
| KCNQ3 |  | 16 |
| KIF18A |  | 16 |
| MEOX1 |  | 16 |
| MRPL41 |  | 16 |
| MSH2 |  | 16 |
| OIP5 |  | 16 |
| PATZ1 |  | 16 |
| PI3 |  | 16 |
| PVRIG |  | 16 |
| QPRT |  | 16 |
| RGL1 |  | 16 |
| RIC3 |  | 16 |
| SDCBP |  | 16 |
| SLCO2A1 |  | 16 |
| TAL2 |  | 16 |
| TMED5 |  | 16 |
| TNFAIP8L2 |  | 16 |
| UBE3D |  | 16 |
| VLDLR |  | 16 |
| WBP1 |  | 16 |
| ZNF219 |  | 16 |
| ACVRL1 |  | 17 |
| BGLAP |  | 17 |
| C11orf20 |  | 17 |
| CCDC150 |  | 17 |
| CLPB |  | 17 |
| COL4A6 |  | 17 |
| CSPG5 |  | 17 |
| DOM3Z |  | 17 |
| FAM189B |  | 17 |
| FANK1 |  | 17 |
| GRM4 |  | 17 |
| HERPUD1 |  | 17 |
| HMGN1 |  | 17 |
| KCNG3 |  | 17 |
| KCNV2 |  | 17 |
| KIAA0319L |  | 17 |
| KRTCAP2 |  | 17 |
| MRPL30 |  | 17 |
| MYOG |  | 17 |
| NDUFB5 |  | 17 |
| NUFIP1 |  | 17 |
| PDXDC1 |  | 17 |
| PEX11G |  | 17 |
| PLEKHM3 |  | 17 |
| PMCH |  | 17 |
| RAD21 |  | 17 |
| RNF130 |  | 17 |
| SLC6A1 |  | 17 |
| SRSF9 |  | 17 |
| TGFB1 |  | 17 |
| TRIAP1 |  | 17 |
| WASH1 |  | 17 |
| XCL2 |  | 17 |
| ZFYVE19 |  | 17 |
| ZNF426 |  | 17 |
| AARSD1 |  | 19 |
| ARHGAP22 |  | 19 |
| ARHGAP44 |  | 19 |
| ATP5I |  | 19 |
| BSPRY |  | 19 |
| C17orf104 |  | 19 |
| C1orf174 |  | 19 |
| CSNK1D |  | 19 |
| DEFB136 |  | 19 |
| DHRS12 |  | 19 |
| GIF |  | 19 |
| KBTBD7 |  | 19 |
| KLK3 |  | 19 |
| LARP4 |  | 19 |
| LCE1C |  | 19 |
| LUM |  | 19 |
| MAZ |  | 19 |
| MESDC1 |  | 19 |
| MMGT1 |  | 19 |
| MRPS26 |  | 19 |
| MTA2 |  | 19 |
| PPAPDC1B |  | 19 |
| RASGEF1B |  | 19 |
| SETDB1 |  | 19 |
| SLC25A35 |  | 19 |
| SLC5A11 |  | 19 |
| SNAI1 |  | 19 |
| SPANXD |  | 19 |
| THAP1 |  | 19 |
| TMOD2 |  | 19 |
| TNFRSF13B |  | 19 |
| TNNT3 |  | 19 |
| TPCN2 |  | 19 |
| TXK |  | 19 |
| UGT3A2 |  | 19 |
| USP51 |  | 19 |
| VPREB3 |  | 19 |
| WDR83 |  | 19 |
| ABHD15 |  | 20 |
| BAG1 |  | 20 |
| C1orf87 |  | 20 |
| CASP8 |  | 20 |
| CLDN10 |  | 20 |
| DENND1A |  | 20 |
| DOCK10 |  | 20 |
| FAM163A |  | 20 |
| HAX1 |  | 20 |
| ITPK1 |  | 20 |
| MAPK3 |  | 20 |
| MICALL1 |  | 20 |
| MXD3 |  | 20 |
| OR5M11 |  | 20 |
| PCDHGA9 |  | 20 |
| PDCD1 |  | 20 |
| RAPGEF1 |  | 20 |
| ROBO3 |  | 20 |
| SPATA24 |  | 20 |
| TBXA2R |  | 20 |
| TMEM154 |  | 20 |
| UTY |  | 20 |
| YEATS4 |  | 20 |
| YWHAQ |  | 20 |
| ZNF837 |  | 20 |
| AGER |  | 21 |
| CBFA2T2 |  | 21 |
| HES3 |  | 21 |
| HSF2BP |  | 21 |
| ISY1 |  | 21 |
| LRRC27 |  | 21 |
| OR2A25 |  | 21 |
| SH3KBP1 |  | 21 |
| TAF7L |  | 21 |
| ZDHHC19 |  | 21 |
| ZNF24 |  | 21 |
| ADAM30 |  | 23 |
| C14orf182 |  | 23 |
| DLX4 |  | 23 |
| DOLPP1 |  | 23 |
| EAF1 |  | 23 |
| HTR1E |  | 23 |
| LCE1E |  | 23 |
| OLIG1 |  | 23 |
| PLD5 |  | 23 |
| PRKAG2 |  | 23 |
| SLC18A1 |  | 23 |
| TTPAL |  | 23 |
| ZNF544 |  | 23 |
| ANGPTL7 |  | 24 |
| ARMCX3 |  | 24 |
| C8orf44 |  | 24 |
| CCDC94 |  | 24 |
| CLEC4G |  | 24 |
| CSHL1 |  | 24 |
| GCG |  | 24 |
| KDELR3 |  | 24 |
| LTC4S |  | 24 |
| OR6B1 |  | 24 |
| SMARCB1 |  | 24 |
| SYTL4 |  | 24 |
| TRMT11 |  | 24 |
| TWIST1 |  | 24 |
| ZNF250 |  | 24 |
| ZNF668 |  | 24 |
| ASAP3 |  | 25 |
| C6orf201 |  | 25 |
| CDCA3 |  | 25 |
| CDKN1A |  | 25 |
| CECR1 |  | 25 |
| DNAJB14 |  | 25 |
| DYNLL2 |  | 25 |
| LATS2 |  | 25 |
| LEFTY1 |  | 25 |
| MRAS |  | 25 |
| MYBPH |  | 25 |
| NGF |  | 25 |
| PALLD |  | 25 |
| PRPF31 |  | 25 |
| RRS1 |  | 25 |
| SH3BP5L |  | 25 |
| SNRK |  | 25 |
| TBC1D23 |  | 25 |
| TRIB2 |  | 25 |
| UBE2Q2 |  | 25 |
| VASN |  | 25 |
| ZFP1 |  | 25 |
| ZNF286A |  | 25 |
| C17orf78 |  | 27 |
| CDX4 |  | 27 |
| DCDC1 |  | 27 |
| DPYSL3 |  | 27 |
| EBAG9 |  | 27 |
| FOXM1 |  | 27 |
| MSX2 |  | 27 |
| PHF19 |  | 27 |
| PSMA1 |  | 27 |
| PTRHD1 |  | 27 |
| REEP5 |  | 27 |
| ZNF398 |  | 27 |
| HIST1H4C |  | 28 |
| TCP10L |  | 28 |
| TLR1 |  | 28 |
| TPM3 |  | 28 |
| USH1G |  | 28 |
| XAGE2B |  | 28 |
| ZNF2 |  | 28 |
| ZNF581 |  | 28 |
| BZW2 |  | 29 |
| CCDC36 |  | 29 |
| CLN8 |  | 29 |
| DLX1 |  | 29 |
| FAM122C |  | 29 |
| FAM125B |  | 29 |
| MAN1A1 |  | 29 |
| NIPAL1 |  | 29 |
| SPANXB1 |  | 29 |
| IWS1 |  | 31 |
| RDBP |  | 31 |
| RPL22L1 |  | 31 |
| SUV420H1 |  | 31 |
| PAX6 |  | 32 |
| ZSCAN23 |  | 32 |
| OTX2 |  | 33 |
| TERF1 |  | 35 |
| HOXA10 |  | 36 |
| PBXIP1 |  | 36 |
| EIF3D |  | 37 |
| NEXN |  | 37 |
| ZNF792 |  | 37 |
| ASF1A |  | 39 |
| CDC14B |  | 39 |
| GLI4 |  | 39 |
| TRNAU1AP |  | 39 |
| HOXC4 |  | 40 |
| CCDC66 |  | 41 |
| LHX1 |  | 43 |
| NAT2 |  | 45 |
| VWC2 |  | 45 |
| YAF2 |  | 47 |
| ZNF232 |  | 48 |
| REXO1L1 |  | 49 |
| MEOX2 |  | 51 |
| ZBTB39 |  | 51 |
| FGF16 |  | 53 |
| VAX2 |  | 55 |
| ABT1 |  | 56 |
| ZNF76 |  | 57 |
| KCNA2 |  | 59 |
| KLC1 |  | 61 |
| ZNF71 |  | 63 |
| KPNA1 |  | 68 |
| ALX3 |  | 71 |
| KPNA6 |  | 72 |
| ZNF329 |  | 73 |
| PHOX2A |  | 85 |
