# Supplementary material for: The cargo adapter protein CLINT1 is phosphorylated by the Numb-associated kinase BIKE and mediates dengue virus infection
Source: J Biol Chem. 2022 Apr 20;298(6):101956. doi: 10.1016/j.jbc.2022.101956 (PMC9133654; doi:10.1016/j.jbc.2022.101956)
Supplement: Supplementary Table S2 [file mmc3.docx]

| Gene | NLR |
| --- | --- |
| DDIT3 | 3.72 +/- 1.05 |
| CDKN1A | 3.01 +/- .33 |
| CUL2 | 1.04 +/- .12 |
| SCO1 | 1/08 +/- .04 |
| GPR87 | 1.67 +/- .21 |
| CKLF | .95 +/- .54 |
| TGIF | 1.18 +/- .32 |
