# Supplementary material for: The cargo adapter protein CLINT1 is phosphorylated by the Numb-associated kinase BIKE and mediates dengue virus infection
Source: J Biol Chem. 2022 Apr 20;298(6):101956. doi: 10.1016/j.jbc.2022.101956 (PMC9133654; doi:10.1016/j.jbc.2022.101956)
Supplement: Supplementary Table S3 [file mmc4.docx]

| **Plasmids** | **Description** | **Source** |
| --- | --- | --- |
| pACYC NGC FL | Renilla luciferase reporter DENV2 infectious clone | [Xie et al., 2013; Zou et al., 2011](https://paperpile.com/c/Qkzmor/uHTh+YvAz) |
| pD2IC-30P-NBX | DENV2 16681 infectious clone | [Huang et al., 2010](https://paperpile.com/c/Qkzmor/U3fU) |
| pCMV-DV2Rep | DENV2 16681 DNA launched replicon | Andrew Yueh |
| AAK1 | HIS tagged AAK1 kinase domain for protein purification | [Sorrell et al., 2016](https://paperpile.com/c/Qkzmor/bZtC) |
| BIKR-c502 | HIS tagged BIKE kinase domain for protein purification | [Sorrell et al., 2016](https://paperpile.com/c/Qkzmor/bZtC) |
| GAK | HIS tagged GAK kinase domain for protein purification | [Sorrell et al., 2016](https://paperpile.com/c/Qkzmor/bZtC) |
| pGEX-CLINT1-1-165 | GST-tagged CLINT1 from amino acid 1 to 165 | [Hirst et al., 2003](https://paperpile.com/c/Qkzmor/ZHR6) |
| pGEX-CLINT1-165-625 | GST-tagged CLINT1 from amino acid 166 to 625 | [Hirst et al., 2003](https://paperpile.com/c/Qkzmor/ZHR6) |
| TC-DENV2 | Tetracysteine tagged DENV2 luciferase virus infectious clone | This study |
| TET-ON |  | Andrew Yueh |
| pTIGHT-DENV2 | DNA lauched DENV2-16681 infectious clone | Pu et al. 2014 |
| TET-OFF |  | Andrew Yueh |
| FLAG-CLINT1 | Flag-tagged wild-type CLINT1 protein for ectopic overexpression | This study |
| FLAG-CLINT1-T294A | Flag-tagged T294A CLINT1 protein for ectopic overexpression | This study |
| GLuc1-CLINT1 | GLuc(1)-tagged wild-type CLINT1 protein | This study |
| GLuc2-BIKE | GLuc(2)-tagged BIKE protein | This study |
| FLAG-RABL2A | Flag-tagged wild-type RABL2A protein for ectopic overexpression | This study |
| FLAG-STAMBP | Flag-tagged wild-type STAMBP protein for ectopic overexpression | This study |
| FLAG-ACAA1 | Flag-tagged wild-type ACAA1 protein for ectopic overexpression | This study |
| FLAG-DAZAP1 | Flag-tagged wild-type DAZAP1 protein for ectopic overexpression | This study |
| FLAG-MYLIP | Flag-tagged wild-type MYLIP protein for ectopic overexpression | This study |
| FLAG-RUNDC3A | Flag-tagged wild-type RUNDC3A protein for ectopic overexpression | This study |
| FLAG-FAM120A | Flag-tagged wild-type FAM120A protein for ectopic overexpression | This study |
| FLAG-MRM1 | Flag-tagged wild-type MRM1 protein for ectopic overexpression | This study |
| GLuc2-PrM | GLUC(2)-tagged DENV PrM protein | This study |
| GLuc2-E | GLUC(2)-tagged DENV envelope protein | This study |
| GLuc2-Capsid | GLUC(2)-tagged DENV capsid protein | This study |
| GLuc2-NS2A | GLUC(2)-tagged DENV NS2A protein | This study |
| GLuc2-NS3 | GLUC(2)-tagged DENV NS3 protein | This study |
| GLuc2-NS4A | GLUC(2)-tagged DENV NS4A protein | This study |
| GLuc2-NS4B | GLUC(2)-tagged DENV NS4B protein | This study |
| GLuc2-NS5 | GLUC(2)-tagged DENV NS5 protein | This study |
| FLAG-NS2B | FLAG-tagged DENV NS2B | This study |
| ACAA1 | Human protein in pDONR223 Gateway Donor Vector | Human ORFeome |
| ADAM17 | Human protein in pDONR223 Gateway Donor Vector | Human ORFeome |
| AP2M1 | Human protein in pDONR223 Gateway Donor Vector | Human ORFeome |
| AP2S1 | Human protein in pDONR223 Gateway Donor Vector | Human ORFeome |
| BAAT | Human protein in pDONR223 Gateway Donor Vector | Human ORFeome |
| BMP3 | Human protein in pDONR223 Gateway Donor Vector | Human ORFeome |
| C1orf63 | Human protein in pDONR223 Gateway Donor Vector | Human ORFeome |
| CHAF-1B | Human protein in pDONR223 Gateway Donor Vector | Human ORFeome |
| CLINT1 | Human protein in pDONR223 Gateway Donor Vector | Human ORFeome |
| CSAD | Human protein in pDONR223 Gateway Donor Vector | Human ORFeome |
| DAZAP1 | Human protein in pDONR223 Gateway Donor Vector | Human ORFeome |
| DGCR6 | Human protein in pDONR223 Gateway Donor Vector | Human ORFeome |
| DLD | Human protein in pDONR223 Gateway Donor Vector | Human ORFeome |
| DNAL4 | Human protein in pDONR223 Gateway Donor Vector | Human ORFeome |
| DYNLT3 | Human protein in pDONR223 Gateway Donor Vector | Human ORFeome |
| EIF4E3 | Human protein in pDONR223 Gateway Donor Vector | Human ORFeome |
| FAM120A | Human protein in pDONR223 Gateway Donor Vector | Human ORFeome |
| FBX034 | Human protein in pDONR223 Gateway Donor Vector | Human ORFeome |
| FBXL3 | Human protein in pDONR223 Gateway Donor Vector | Human ORFeome |
| GJD3 | Human protein in pDONR223 Gateway Donor Vector | Human ORFeome |
| HBB | Human protein in pDONR223 Gateway Donor Vector | Human ORFeome |
| ING2 | Human protein in pDONR223 Gateway Donor Vector | Human ORFeome |
| INTS4 | Human protein in pDONR223 Gateway Donor Vector | Human ORFeome |
| LIMS1 | Human protein in pDONR223 Gateway Donor Vector | Human ORFeome |
| LYSMD3 | Human protein in pDONR223 Gateway Donor Vector | Human ORFeome |
| LYZL4 | Human protein in pDONR223 Gateway Donor Vector | Human ORFeome |
| MRM1 | Human protein in pDONR223 Gateway Donor Vector | Human ORFeome |
| MTA1 | Human protein in pDONR223 Gateway Donor Vector | Human ORFeome |
| MYLIP | Human protein in pDONR223 Gateway Donor Vector | Human ORFeome |
| NAA15 | Human protein in pDONR223 Gateway Donor Vector | Human ORFeome |
| NECAB1 | Human protein in pDONR223 Gateway Donor Vector | Human ORFeome |
| NFKBIL | Human protein in pDONR223 Gateway Donor Vector | Human ORFeome |
| NRG2 | Human protein in pDONR223 Gateway Donor Vector | Human ORFeome |
| PCNP | Human protein in pDONR223 Gateway Donor Vector | Human ORFeome |
| PDCD5 | Human protein in pDONR223 Gateway Donor Vector | Human ORFeome |
| PICALM | Human protein in pDONR223 Gateway Donor Vector | Human ORFeome |
| PKNOX2 | Human protein in pDONR223 Gateway Donor Vector | Human ORFeome |
| RABL2A | Human protein in pDONR223 Gateway Donor Vector | Human ORFeome |
| RALBP1 | Human protein in pDONR223 Gateway Donor Vector | Human ORFeome |
| REPS1 | Human protein in pDONR223 Gateway Donor Vector | Human ORFeome |
| RUNDC3A | Human protein in pDONR223 Gateway Donor Vector | Human ORFeome |
| SCGB1C1 | Human protein in pDONR223 Gateway Donor Vector | Human ORFeome |
| SH2D4A | Human protein in pDONR223 Gateway Donor Vector | Human ORFeome |
| SHISA3 | Human protein in pDONR223 Gateway Donor Vector | Human ORFeome |
| SIGLEC7 | Human protein in pDONR223 Gateway Donor Vector | Human ORFeome |
| SIRT2 | Human protein in pDONR223 Gateway Donor Vector | Human ORFeome |
| SLC25A41 | Human protein in pDONR223 Gateway Donor Vector | Human ORFeome |
| STAMBP | Human protein in pDONR223 Gateway Donor Vector | Human ORFeome |
| STAMBPL1 | Human protein in pDONR223 Gateway Donor Vector | Human ORFeome |
| STGGAL1 | Human protein in pDONR223 Gateway Donor Vector | Human ORFeome |
| STXBP6 | Human protein in pDONR223 Gateway Donor Vector | Human ORFeome |
| TACSTD2 | Human protein in pDONR223 Gateway Donor Vector | Human ORFeome |
| TRAPPC-6B | Human protein in pDONR223 Gateway Donor Vector | Human ORFeome |
| TRIM56 | Human protein in pDONR223 Gateway Donor Vector | Human ORFeome |
| ZDHHC22 | Human protein in pDONR223 Gateway Donor Vector | Human ORFeome |
| ZNF468 | Human protein in pDONR223 Gateway Donor Vector | Human ORFeome |
| pX458 gRNA | Addgene plasmid # 48138 | Gift from Dr. Feng Zhang |
