# Supplementary material for: The cargo adapter protein CLINT1 is phosphorylated by the Numb-associated kinase BIKE and mediates dengue virus infection
Source: J Biol Chem. 2022 Apr 20;298(6):101956. doi: 10.1016/j.jbc.2022.101956 (PMC9133654; doi:10.1016/j.jbc.2022.101956)
Supplement: Supplementary Table S4 [file mmc5.docx]

| **siRNA** | **Description** | **Source** | **Catalog number** | **Sequence** |
| --- | --- | --- | --- | --- |
| BIKE | siGENOME Human BMP2K (55589) siRNA SMART Pool | Dharmacon | M-005071-01-0010 | GAACAUAGACCUGAUAUAU GGACUGUGCUGUUAAUUCA GGAACUAUGUACUUUGUGA CGAUGUGCAUUGAAGCGAA |
| Non-targeting | siGENOME Non-Targeting siRNA Pool #1 | Dharmacon | D-001206-13-05 | UAGCGACUAAACACAUCAA UAAGGCUAUGAAGAGAUAC AUGUAUUGGCCUGUAUUAG AUGAACGUGAAUUGCUCAA |
| CLINT1 | CLINT1 (EpsinR) siRNA (ONtarget siRNA) | Dharmacon | QTE-2568052G | AAUACAGAUAUGGUCCAGAAA |
| Non-targeting | ONtarget NT siRNA | Dharmacon | D-001810-10-05 | AAUACAGAUAUGGUCCAGAAA UGGUUUACAUGUUGUGUGA UGGUUUACAUGUUUUCUGA UGGUUUACAUGUUUUCCUA |

| **Antibodies** | **Source** | **Catalog Number** |
| --- | --- | --- |
| anti-FLAG | Sigma-Aldrich | F7425 |
| anti-CLINT1 | Bethyl | A301-926A |
| anti-BIKE | Santa Cruz Biotechnology | sc-134284 |
| anti-GLuc | New England BioLabs | E8023S |
| anti–β-actin | Sigma-Aldrich | A3854 |
| anti-STK16 | Santa Cruz Biotechnology | sc-374356 |
| anti-DENV Capsid | GeneTex | GTX633632 |
| anti-DENV NS1 | GeneTex | GTX124280 |
| Anti-mouse IgG, HRP-linked | Cell Signaling | 7076S |
| Anti-Rabbit IgG HRP-linked | Cell Signaling | 7074s |
| Anti-Flag Affinity Gel | Bimake | B23101 |
| Mouse IgG−Agarose | Sigma-Aldrich | A0919 |

| **Primer** | **Sequence** | **Note** |
| --- | --- | --- |
| PY-D2-1/F | AGTTGTTAGTCTACGTGGACCGACAAAGAC | Primer for tetracysteine tagged virus |
| PY+FLN-TC_163/R | AGGTTCCATACAACAGCCAGGACAACAATTGAGAAAGCGGTTTCTCTCGCGTTTC | Primer for tetracysteine tagged virus |
| PY+FLN-TC_198/F | TTTCTCAATTGTTGTCCTGGCTGTTGTATGGAACCTCTTGGAATGCTGCAGGGAC | Primer for tetracysteine tagged virus |
| PY_LucDen2/SphI/R | CTCTGTGGTGTTATTTTG | Primer for tetracysteine tagged virus |
| pTight/F | CGAGGTAGGCGTGTACGG | Alanine scanning over lapping PCR forward primer |
| 16681/1434/R | TGGTGTTATTTTGATTTC | Alanine scanning over lapping PCR reverse primer |
| 1.FWD | AAACGCGAGAGAAACCGCGCAGCTGCGGTGCAACAGCTGACAAAG | Mutagenesis primer for clone 1 of alanine scanning mutagenesis |
| 1.REV | CTTTGTCAGCTGTTGCACCGCAGCTGCGCGGTTTCTCTCGCGTTT | Mutagenesis primer for clone 1 of alanine scanning mutagenesis |
| 2.FWD | AGAAACCGCGTGTCGACTGCAGCTGCGCTGACAAAGAGATTCTCA | Mutagenesis primer for clone 2 of alanine scanning mutagenesis |
| 2.REV | TGAGAATCTCTTTGTCAGCGCAGCTGCAGTCGACACGCGGTTTCT | Mutagenesis primer for clone 2 of alanine scanning mutagenesis |
| 3.FWD | GTGTCGACTGTGCAACAGGCAGCTGCGAGATTCTCACTTGGAATG | Mutagenesis primer for clone 3 of alanine scanning mutagenesis |
| 3.REV | CATTCCAAGTGAGAATCTCGCAGCTGCCTGTTGCACAGTCGACAC | Mutagenesis primer for clone 3 of alanine scanning mutagenesis |
| 4.FWD | GTGCAACAGCTGACAAAGGCAGCTGCGCTTGGAATGCTGCAGGG | Mutagenesis primer for clone 4 of alanine scanning mutagenesis |
| 4.REV | CCCTGCAGCATTCCAAGCGCAGCTGCCTTTGTCAGCTGTTGCAC | Mutagenesis primer for clone 4 of alanine scanning mutagenesis |
| 5.FWD | CTGACAAAGAGATTCTCAGCAGCTGCGCTGCAGGGACGAGGACC | Mutagenesis primer for clone 5 of alanine scanning mutagenesis |
| 5.REV | GGTCCTCGTCCCTGCAGCGCAGCTGCTGAGAATCTCTTTGTCAG | Mutagenesis primer for clone 5 of alanine scanning mutagenesis |
| 6.FWD | AGATTCTCACTTGGAATGGCAGCTGCGCGAGGACCATTAAAACTG | Mutagenesis primer for clone 6 of alanine scanning mutagenesis |
| 6.REV | CAGTTTTAATGGTCCTCGCGCAGCTGCCATTCCAAGTGAGAATCT | Mutagenesis primer for clone 6 of alanine scanning mutagenesis |
| 7.FWD | CTTGGAATGCTGCAGGGAGCAGCTGCGTTAAAACTGTTCATGGCC | Mutagenesis primer for clone 7 of alanine scanning mutagenesis |
| 7.REV | GGCCATGAACAGTTTTAACGCAGCTGCTCCCTGCAGCATTCCAAG | Mutagenesis primer for clone 7 of alanine scanning mutagenesis |
| 8.FWD | CTGCAGGGACGAGGACCAGCAGCTGCGTTCATGGCCCTGGTGGCG | Mutagenesis primer for clone 8 of alanine scanning mutagenesis |
| 8.REV | CGCCACCAGGGCCATGAACGCAGCTGCTGGTCCTCGTCCCTGCAG | Mutagenesis primer for clone 8 of alanine scanning mutagenesis |
| 9.FWD | CGAGGACCATTAAAACTGGCAGCTGCGCTGGTGGCGTTCCTTCG | Mutagenesis primer for clone 9 of alanine scanning mutagenesis |
| 9.REV | CGAAGGAACGCCACCAGCGCAGCTGCCAGTTTTAATGGTCCTCG | Mutagenesis primer for clone 9 of alanine scanning mutagenesis |
| 10.FWD | TTAAAACTGTTCATGGCCGCAGCTGCGTTCCTTCGTTTCCTAACAATC | Mutagenesis primer for clone 10 of alanine scanning mutagenesis |
| 10.REV | GATTGTTAGGAAACGAAGGAACGCAGCTGCGGCCATGAACAGTTTTAA | Mutagenesis primer for clone 10 of alanine scanning mutagenesis |
| 11.FWD | GTTCATGGCCCTGGTGGCGGCAGCTGCGTTCCTAACAATCCCACC | Mutagenesis primer for clone 11 of alanine scanning mutagenesis |
| 11.REV | GGTGGGATTGTTAGGAACGCAGCTGCCGCCACCAGGGCCATGAAC | Mutagenesis primer for clone 11 of alanine scanning mutagenesis |
| 12.FWD | CTGGTGGCGTTCCTTCGTGCAGCTGCGATCCCACCAACAGCAGGG | Mutagenesis primer for clone 12 of alanine scanning mutagenesis |
| 12.REV | CCCTGCTGTTGGTGGGATCGCAGCTGCACGAAGGAACGCCACCAG | Mutagenesis primer for clone 12 of alanine scanning mutagenesis |
| 13.FWD | GTTCCTTCGTTTCCTAACAGCAGCTGCGACAGCAGGGATATTGAAG | Mutagenesis primer for clone 13 of alanine scanning mutagenesis |
| 13.REV | CTTCAATATCCCTGCTGTCGCAGCTGCTGTTAGGAAACGAAGGAAC | Mutagenesis primer for clone 13 of alanine scanning mutagenesis |
| 14.FWD | GTTTCCTAACAATCCCACCAGCAGCTGCGATATTGAAGAGATGGGGAAC | Mutagenesis primer for clone 14 of alanine scanning mutagenesis |
| 14.REV | GTTCCCCATCTCTTCAATATCGCAGCTGCTGGTGGGATTGTTAGGAAAC | Mutagenesis primer for clone 14 of alanine scanning mutagenesis |
| 15.FWD | CAATCCCACCAACAGCAGGGGCAGCTGCGAGATGGGGAACAATTAAA | Mutagenesis primer for clone 15 of alanine scanning mutagenesis |
| 15.REV | TTTAATTGTTCCCCATCTCGCAGCTGCCCCTGCTGTTGGTGGGATTG | Mutagenesis primer for clone 15 of alanine scanning mutagenesis |
| 16.FWD | CAGCAGGGATATTGAAGGCAGCTGCGACAATTAAAAAATCAAAAG | Mutagenesis primer for clone 16 of alanine scanning mutagenesis |
| 16.REV | CTTTTGATTTTTTAATTGTCGCAGCTGCCTTCAATATCCCTGCTG | Mutagenesis primer for clone 16 of alanine scanning mutagenesis |
| 17.FWD | CAATTAAAAAATCAAAAGCAGCTGCGGTTTTGAGAGGGTTCAGG | Mutagenesis primer for clone 17 of alanine scanning mutagenesis |
| 17.REV | CCTGAACCCTCTCAAAACCGCAGCTGCTTTTGATTTTTTAATTG | Mutagenesis primer for clone 17 of alanine scanning mutagenesis |
| 18.FWD | AAATCAAAAGCTATTAATGCAGCTGCGGGGTTCAGGAAAGAGATTG | Mutagenesis primer for clone 18 of alanine scanning mutagenesis |
| 18.REV | CAATCTCTTTCCTGAACCCCGCAGCTGCATTAATAGCTTTTGATTT | Mutagenesis primer for clone 18 of alanine scanning mutagenesis |
| 19.FWD | GCTATTAATGTTTTGAGAGCAGCTGCGAAAGAGATTGGAAGGATG | Mutagenesis primer for clone 19 of alanine scanning mutagenesis |
| 19.REV | CATCCTTCCAATCTCTTTCGCAGCTGCTCTCAAAACATTAATAGC | Mutagenesis primer for clone 19 of alanine scanning mutagenesis |
| 20.FWD | GTTTTGAGAGGGTTCAGGGCAGCTGCGGGAAGGATGCTGAACATC | Mutagenesis primer for clone 20 of alanine scanning mutagenesis |
| 20.REV | GATGTTCAGCATCCTTCCCGCAGCTGCCCTGAACCCTCTCAAAAC | Mutagenesis primer for clone 20 of alanine scanning mutagenesis |
| 21.FWD | GGGTTCAGGAAAGAGATTGCAGCTGCGCTGAACATCTTGAATAGG | Mutagenesis primer for clone 21 of alanine scanning mutagenesis |
| 21.REV | CCTATTCAAGATGTTCAGCGCAGCTGCAATCTCTTTCCTGAACCC | Mutagenesis primer for clone 21 of alanine scanning mutagenesis |
| 22.FWD | GAAAGAGATTGGAAGGATGGCAGCTGCGTTGAATAGGAGACGCAG | Mutagenesis primer for clone 22 of alanine scanning mutagenesis |
| 22.REV | CTGCGTCTCCTATTCAACGCAGCTGCCATCCTTCCAATCTCTTTC | Mutagenesis primer for clone 22 of alanine scanning mutagenesis |
| 23.FWD | GGAAGGATGCTGAACATCGCAGCTGCGAGACGCAGATCTGCCGGC | Mutagenesis primer for clone 23 of alanine scanning mutagenesis |
| 23.REV | GCCGGCAGATCTGCGTCTCGCAGCTGCGATGTTCAGCATCCTTCC | Mutagenesis primer for clone 23 of alanine scanning mutagenesis |
| 24.FWD | CTGAACATCTTGAATAGGGCAGCTGCGTCTGCCGGCATGATCATT | Mutagenesis primer for clone 24 of alanine scanning mutagenesis |
| 24.REV | AATGATCATGCCGGCAGACGCAGCTGCCCTATTCAAGATGTTCAG | Mutagenesis primer for clone 24 of alanine scanning mutagenesis |
