# Supplementary figures and images for: The cargo adapter protein CLINT1 is phosphorylated by the Numb-associated kinase BIKE and mediates dengue virus infection
Source: J Biol Chem. 2022 Apr 20;298(6):101956. doi: 10.1016/j.jbc.2022.101956 (PMC9133654; doi:10.1016/j.jbc.2022.101956)

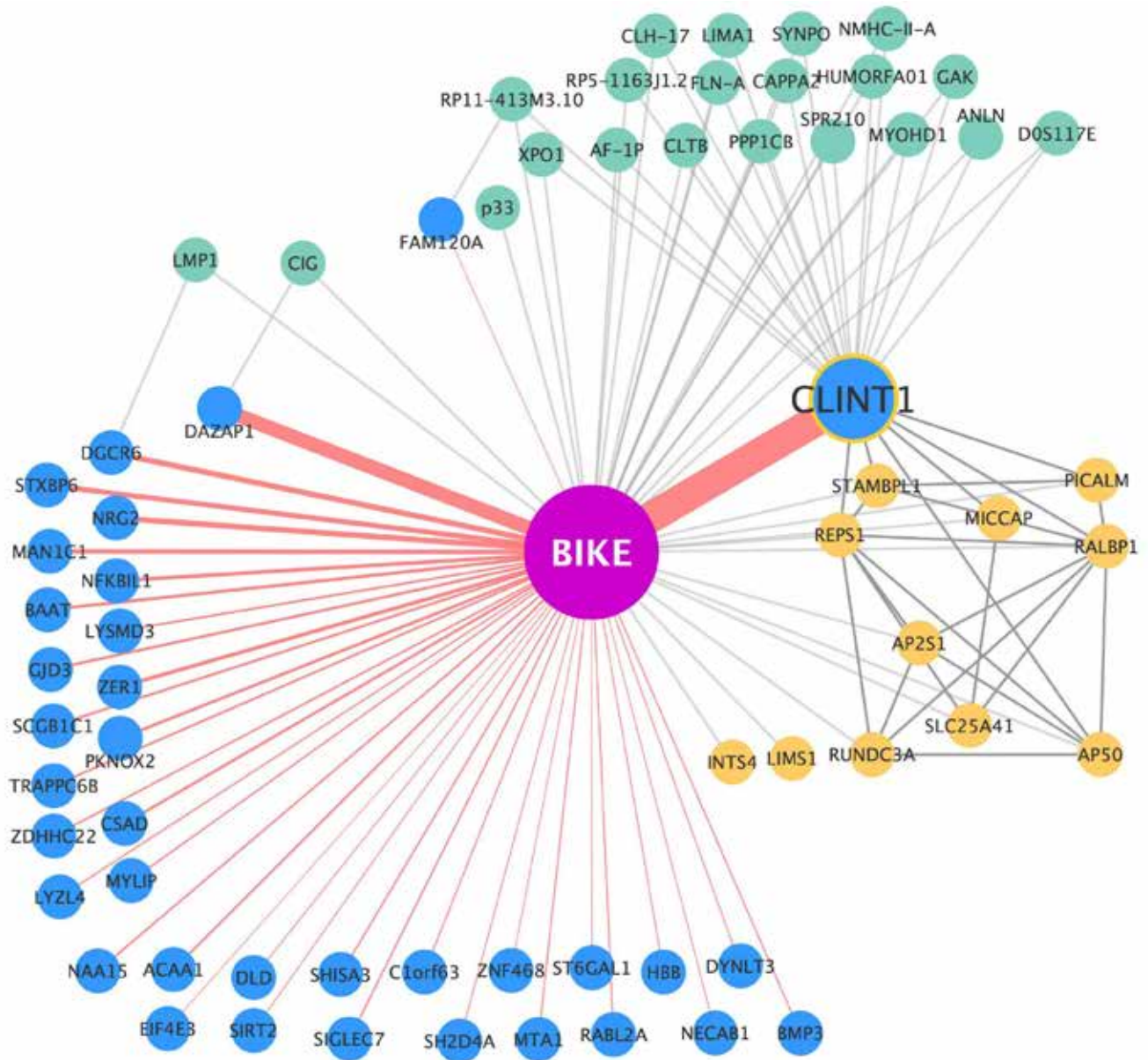

Supplement: Supplementary Figure S1 [file mmc7.pdf]

**Fig S2**

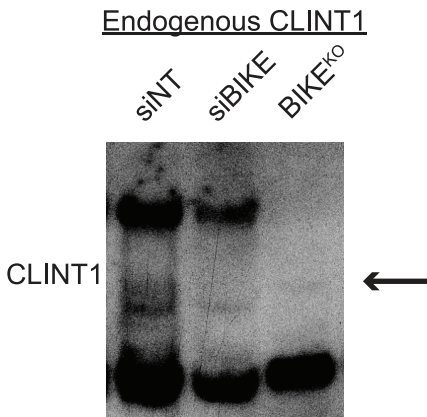

Supplement: Supplementary Figure S2 [file mmc8.pdf]

**Fig S4**

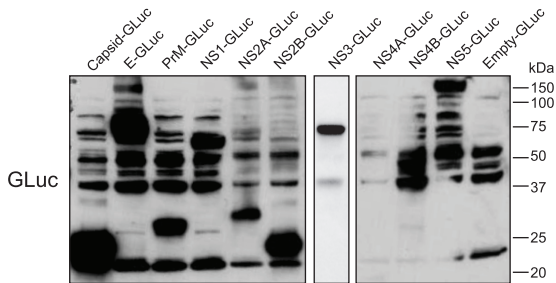

Supplement: Supplementary Figure S3 [file mmc9.pdf]

**Fig S6**

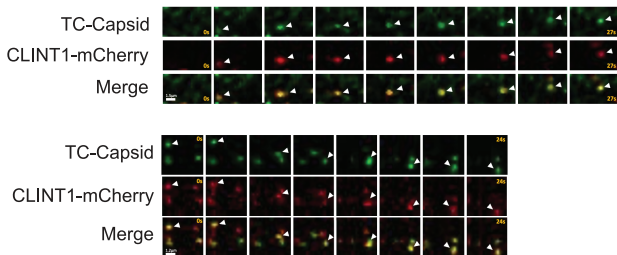

Supplement: Supplementary Figure S5 [file mmc11.pdf]

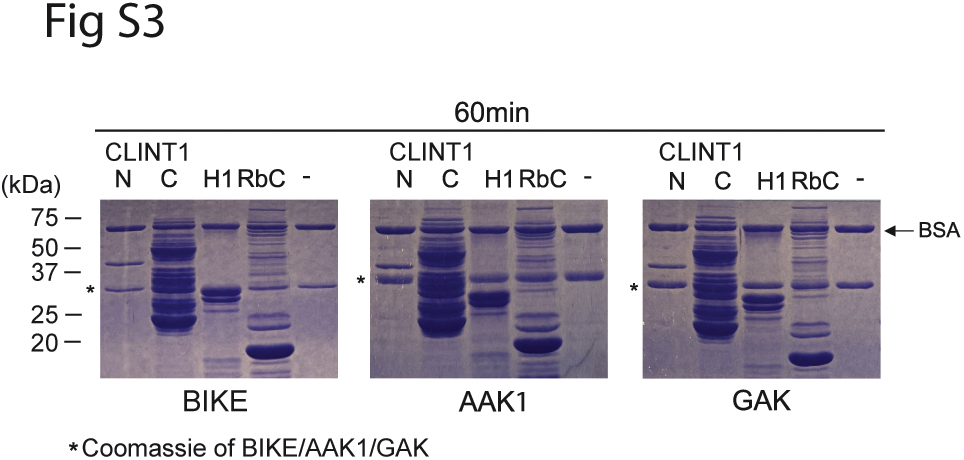

Supplement: Supplementary Figure S6 [file figs1.jpg]
