# Supplementary material for: The cargo adapter protein CLINT1 is phosphorylated by the Numb-associated kinase BIKE and mediates dengue virus infection
Source: J Biol Chem. 2022 Apr 20;298(6):101956. doi: 10.1016/j.jbc.2022.101956 (PMC9133654; doi:10.1016/j.jbc.2022.101956)
Supplement: Supplementary Figure S4 [file mmc10.pdf]

Fig S5

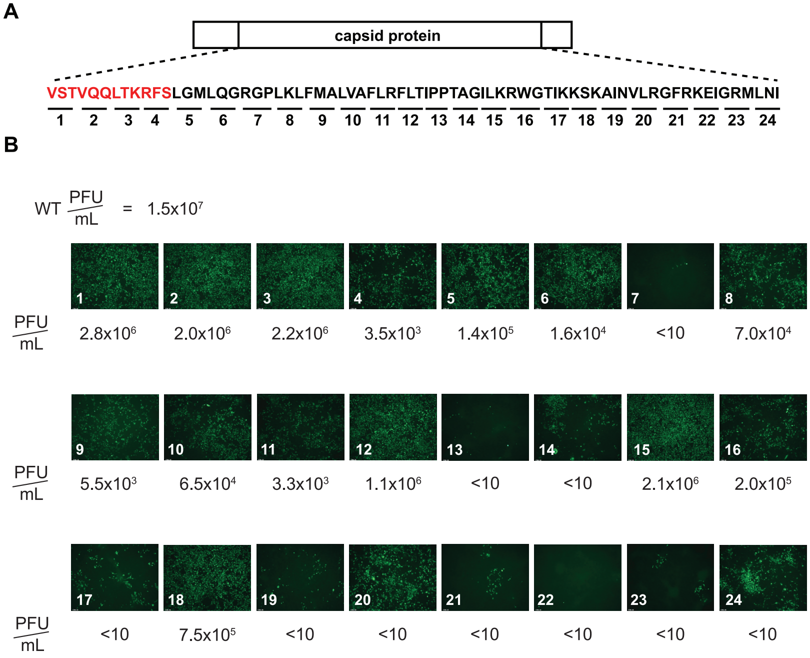
A
capsid protein
VSTVQQLTKRFSLGMLQGRGPLKLFMALVAFLRFLTIPPTAGILKRWGTIKKSKAINVLRGFRKEIGRMLNI
1 2 3 4 5 6 7 8 9 10 11 12 13 14 15 16 17 18 19 20 21 22 23 24
B
WT PFU/mL = 1.5x10^7
1 2 3 4 5 6 7 8
PFU/mL 2.8x10^6 2.0x10^6 2.2x10^6 3.5x10^3 1.4x10^5 1.6x10^4 <10 7.0x10^4
9 10 11 12 13 14 15 16
PFU/mL 5.5x10^3 6.5x10^4 3.3x10^3 1.1x10^6 <10 <10 2.1x10^6 2.0x10^5
17 18 19 20 21 22 23 24
PFU/mL <10 7.5x10^5 <10 <10 <10 <10 <10 <10
